# Evolutionary innovation within conserved gene regulatory networks underlying biomineralized skeletons in Bilateria

**DOI:** 10.1093/molbev/msag019

**Published:** 2026-01-20

**Authors:** Yitian Bai, Yue Min, Shikai Liu, Yiming Hu, Shulei Jin, Hong Yu, Lingfeng Kong, Daniel J Macqueen, Shaojun Du, Qi Li

**Affiliations:** Key Laboratory of Mariculture, Ministry of Education, Ocean University of China, Qingdao 266003, China; Key Laboratory of Mariculture, Ministry of Education, Ocean University of China, Qingdao 266003, China; Key Laboratory of Mariculture, Ministry of Education, Ocean University of China, Qingdao 266003, China; Key Laboratory of Mariculture, Ministry of Education, Ocean University of China, Qingdao 266003, China; Key Laboratory of Mariculture, Ministry of Education, Ocean University of China, Qingdao 266003, China; Key Laboratory of Mariculture, Ministry of Education, Ocean University of China, Qingdao 266003, China; Key Laboratory of Mariculture, Ministry of Education, Ocean University of China, Qingdao 266003, China; The Roslin Institute and Royal (Dick) School of Veterinary Studies, The University of Edinburgh, Midlothian, UK; Institute of Marine and Environmental Technology, Department of Biochemistry and Molecular Biology, University of Maryland School of Medicine, Baltimore, MD, USA; Key Laboratory of Mariculture, Ministry of Education, Ocean University of China, Qingdao 266003, China; Laboratory for Marine Fisheries Science and Food Production Processes, Qingdao Marine Science and Technology Center, Qingdao 266237, China

**Keywords:** biomineralization, evolution of gene regulatory networks, chromatin dynamics, transcription factor, shell formation

## Abstract

Biomineralized skeletons have evolved convergently across animals and exhibit remarkable diversity in structure and development. However, the evolutionary origins of gene regulatory networks underlying biomineralized skeletons remain elusive. Here, we report comprehensive developmental profiling of transcriptomic and chromatin dynamics in a bivalve mollusc, *Crassostrea nippona*. We provide evidence for a biphasic regulatory program orchestrating larval and adult shell formation, involving the coordinated activity of ancient transcription factors and dynamic chromatin remodeling. Comparative analyses suggest a conserved developmental toolkit was co-opted for larval exoskeleton formation in the common lophotrochozoan ancestor. In contrast, limited regulatory conservation was observed between lophotrochozoans and echinoderms with regard to the formation of biomineralized skeletons, despite both relying on a heterochronic activation of ancestral regulators. Together, our findings support a hierarchical model in which dynamic chromatin decouples rapidly evolving effectors from deeply conserved regulators, allowing modular innovations within conserved gene regulatory networks. This study highlights how epigenetic dynamics bridge evolutionary conservation and novelty, offering a framework for understanding the independent evolution of biomineralization across Bilateria through combinatorial regulatory evolution.

## Introduction

**Figure 1:**
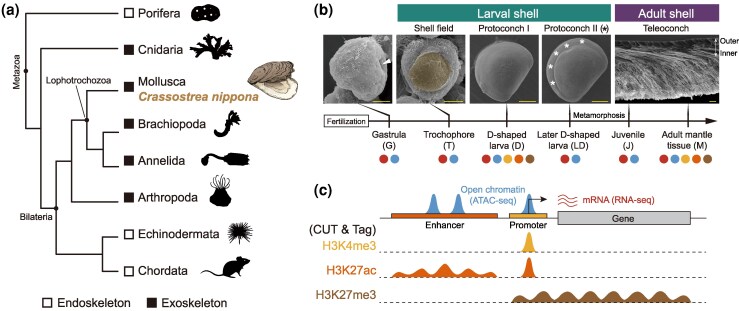
Diversity of biomineralized skeletons across metazoan lineages and developmental stages. a) Schematic phylogeny illustrates the distribution of biomineralized skeletons among representative metazoan lineages. The silhouette images of animals in this figure, except for the oyster, were modified from PhyloPic (https://www.phylopic.org/), an open database of free silhouette images available for reuse under Creative Commons licenses. b) Experimental design. Top: scanning electron micrographs (SEMs) of *C. nippona* shells at representative developmental stages. The blastopore (arrow) formed at the gastrula stage (8 hours post fertilization, hpf). At the trochophore stage (14 hpf), the shell field (yellow) covers the dorsal region. The uniform calcified shell surface of protoconch I was observed at the D-shaped stage (22 hpf). Growth striations (asterisks) of protoconch II appeared on the shell surface at 2 days post fertilization (dpf). The adult shell comprises three shell layers: outer prismatic layer, and inner foliated and chalky layers. Scale bar = 20 μm. Detailed observations of larval and adult shells are shown in Figure S1. Bottom: samples for RNA-seq (red), ATAC-seq (blue), and each histone modification (yellow for H3K4me3; orange for H3K27ac; brown for H3K27me3) are represented by color-coded dots. c) A schematic illustrating representative peak signals of accessible chromatin and three histone modifications. The orange and yellow boxes indicate enhancer and promoter regions, respectively, while the grey box indicates the gene body.

Many animals produce biomineralized skeletons for protection and physical support. This process represents a classic example of functional convergence, with distinct metazoan phyla independently evolving biomineralized skeletons multiple times since the early Cambrian (Gilbert et al. 2022). Despite their divergent origins, biomineralized systems share strikingly similar strategies for utilizing organic matrices and amorphous precursors to construct diverse skeletal architectures (Marin et al. 2016; Gilbert et al. 2019; Murdock 2020), including exoskeletons (e.g. coral skeletons, molluscan and brachiopod shells) and endoskeletons (e.g. sponge and echinoderm spicules, vertebrate bones) (Fig. 1a). Such convergence is supposedly derived from a conserved “biomineralization toolkit”, comprising a set of ancestral genes and regulatory elements co-opted across lineages (Murdock 2020). However, the origins and homology of these toolkits and their associated gene regulatory networks (GRNs), both across and within phyla, remain poorly understood. Resolving these questions has implications for understanding the molecular mechanisms underlying the evolution of biological complexity and diversity.

Skeletal formation commonly initiates during early development (embryonic or larval stages) and exhibits ontogenetic plasticity, transitioning from transient larval frameworks to complex adult biomineralized architectures (Weiss et al. 2002; Uriz et al. 2003; Akiva et al. 2018; McDougall and Degnan 2018; Nanglu et al. 2023). This dynamic remodeling reflects adaptive responses to ecological pressures and physiological demands throughout the life cycle (Gilbert et al. 2022). Most studies have focused on echinoderms and chordates (Rafiq et al. 2014; Jandzik et al. 2015; Davidson et al. 2022; To et al. 2024), revealing the independent co-option of ancestral developmental GRNs for skeletogenesis across different deuterostome clades (Dylus et al. 2018; Erkenbrack and Thompson 2019; Morgulis et al. 2019; Nanglu et al. 2023). Skeletogenic programs of deuterostomes appear to have evolved through the activation of phylum-specific regulatory and differentiation genes from modifications and rewiring of ancestral GRNs (Dylus et al. 2018; Ben-Tabou de-Leon 2022; Meyer et al. 2023). Yet, biomineralization GRNs remain largely underrepresented across non-deuterostome lineages and their life stages.

Mollusca is a large and diverse phylum, and its extraordinary morphological diversity is largely attributed to the remarkable variety of biomineralized shells (Clark et al. 2020). A large number of rapidly evolving and co-opted genes, such as shell matrix proteins (SMPs), biomineralization enzymes, and transporters, have been identified as downstream effectors involved in shaping the diversity of adult shell structures (Kocot et al. 2016; Aguilera et al. 2017; Yarra et al. 2021; Li et al. 2024). Larval shells are more conserved than adult shells in terms of morphology, crystal polymorph, and microstructure across lineages (Weiss et al. 2002; McDougall and Degnan 2018; Cavallo et al. 2022). This phenotypic conservation likely results from deeply conserved upstream GRNs governing early shell development (Sleight 2023). Supporting this hypothesis, recent work demonstrated an ancestral co-option of Hox genes in larval shell development programs across molluscan clades (Huan et al. 2020). Intriguingly, proteomic comparisons revealed almost entirely distinct SMPs repertoires between larval and adult stages (Zhao et al. 2018; Carini et al. 2019; Cavallo et al. 2022), indicating that distinct sets of downstream effectors are deployed at different life stages, even within the same species. Nonetheless, it remains plausible that shared upstream GRNs, typically transcription factors (TFs), orchestrate skeletal formation across ontogeny (Soares et al. 2013; Dylus et al. 2018; Erkenbrack and Thompson 2019), with stage-specific regulatory modifications directing divergent downstream targets. Even though there have been efforts to predict GRNs for adult shell formation in molluscs such as *Laternula elliptica* (Sleight et al. 2020), the current lack of (1) epigenomic profiling of cis-regulatory elements and (2) shell formation GRNs spanning both larval and adult stages, limits understanding of the dynamics underlying molluscan biomineralization strategies across ontogeny and evolution.

To address these questions comprehensively, we profiled transcriptomic and genome-wide chromatin dynamics across multiple developmental stages of a bivalve mollusc, *Crassostrea* (also called *Magallana*) *nippona*, with a focus on early larval and adult shell formation (Fig. 1b and c). We discovered a novel biphasic regulatory program wherein stage-specific chromatin landscape transitions enable conserved upstream TFs to regulate distinct sets of downstream biomineralization genes in larvae and adults. By integrating transcriptomic, chromatin accessibility, and TF footprinting analyses, we identify stage-specific GRNs, revealing an overlapping group of TFs that orchestrate this biphasic regulatory program. These TFs likely constitute a conserved molecular toolkit for exoskeleton formation in lophotrochozoan larvae, with some independently co-opted for early skeletogenesis in echinoderms. Despite both systems relying on the heterochronic activation of ancestral regulators, the formation of biomineralized skeleton in lophotrochozoans and echinoderms shows limited regulatory conservation.

## Results

### Transcriptomic dynamics during larval and adult shell formation

**Figure 2:**
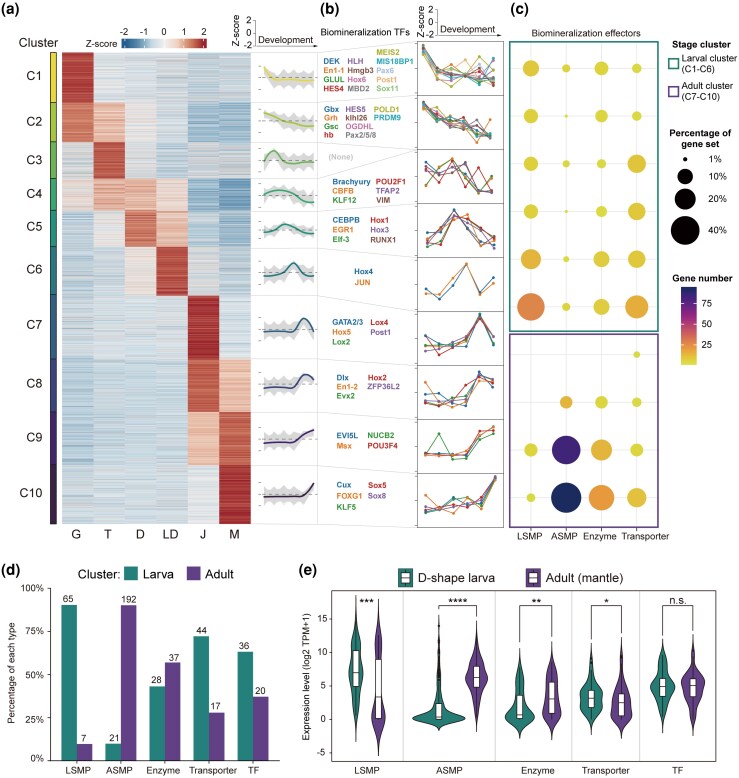
Transcriptomic atlas of biomineralization across *C. nippona* ontogeny. a) K-means clustering of stage-specific highly expressed genes. On the right of heatmap, gene-wise expression dynamics (grey lines) and locally estimated scatterplot smoothing (colored lines) are shown for each cluster. G, gastrula; T, trochophore; D, D-shaped larva; LD, later D-shaped larva; J, juvenile; M, adult mantle. b) Expression patterns (right) of previously reported putative biomineralization TF genes involved in molluscan shell formation (left) in each cluster. *Y* axis represents Z-score normalized gene expression. c) Distribution of highly expressed downstream biomineralization effector genes across clusters. Dot size represents the percentage of downstream effector genes relative to the total number of genes in each effector category. Color indicates the gene number. Green and purple boxes highlight larval (clusters C1–C6) and adult clusters (clusters C7–C10), respectively. Detailed information is shown in Table S5. LSMP, larval shell matrix protein; ASMP, adult shell matrix protein. d) Number and percentage of each category of biomineralization effector genes within larval and adult clusters. The numbers on each bar indicate gene count. Abbreviations are same as (**a)**. e) Comparison of expression levels for each effector gene category between D-shape larva and adult stages. Boxplots include the median with quartiles and outliers above the top whisker. A two-sided Wilcoxon rank-sum test was used to assess significance (*P < 0.05; **P < 0.01; ***P < 0.001; ****P < 0.0001; n.s., not significant). Abbreviations are same as (**a)**.

The oyster goes through several morphologically distinct developmental stages, with shell formation exhibiting major phenotypic differences: the shell field and protoconch I, II during early development (collectively referred to as larval shells in this study), and teleoconch after metamorphosis (referred to as the adult shell) (Fig. 1b and Figure S1). To obtain a reliable gene annotation across oyster development, we generated a new full-length transcriptome from mixed-stage mRNA samples covering six representative shell-forming stages in *C. nippona* using Iso-seq, resulting in the identification of 29,577 gene models (Figures S2 and S3, Table S1, and Note S1). To investigate the global dynamics of gene expression during larval and adult shell formation, we performed RNA-seq at these stages (Fig. 1b and c, Figure S4a and b, Table S2). We identified 25,465 genes expressed during at least one stage (transcripts per million, TPM, > 1), which were divided into ten clusters using *K*-means clustering (Fig. 2a). Gene ontology (GO) enrichment analysis of these clusters highlighted major developmental processes associated with shell formation (Figure S5, Table S3, and Note S2). We found GO terms related to calcium ion transport and the extracellular matrix during the D-shape larva and adult stages (clusters C5, C6, C7, and C9), as well as peptidase inhibitor activity at the adult stage (cluster C9), suggesting the functional importance of these stages in shell formation (Wang et al. 2020; Yarra et al. 2021).

To understand the transcriptional programs governing the expression of biomineralization genes at larval and adult stages, we identified 56 putative biomineralization TF genes implicated in molluscan shell formation (Table S4) and 382 biomineralization effector genes (Table S5), including larval shell matrix proteins (LSMPs), adult shell matrix proteins (ASMPs), biomineralization enzymes, and transporters. Biomineralization TF genes generally exhibited ubiquitous expression across stages (Fig. 2b and Figure S4c). In contrast, effector genes showed stage-specific expression during larval and adult shell formation (Fig. 2c and d, Figure S6 and S7, Table S5). Most LSMP genes (90.3%) were enriched in larval clusters and highly expressed during larval shell formation (Fig. 2c and d, Figure S6a), while ASMP genes (90.1%) were predominantly grouped into adult clusters and showed high expression levels during adult shell formation (Fig. 2c and d, Figure S6b). Similarly, genes encoding biomineralization enzymes and transporters showed distinct expression between larval and adult stages (Fig. 2c and d, Figure S7a). Thus, transcriptional programs regulating biomineralization effector genes involved in shell formation are temporally regulated at distinct life stages (Zhao et al. 2018; Cavallo et al. 2022), resulting in the morphological divergence between larval and adult shells. Strikingly, despite the significant stage-specific expression differences of effector genes, particularly between the D-shape larva and adult stages, TF genes maintain relatively stable expression levels with no significant variation across developmental stages (Fig. 2e, Figure S7b and c, Table S6). Therefore, we hypothesized that these TFs play a foundational role in controlling two major transcriptional programs that separately drive larval and adult shell formation.

### A biphasic regulatory program for larval and adult shell formation

To delve into the transcriptional regulation of larval and adult shell formation, we applied the assay for transposase-accessible chromatin sequencing (ATAC-seq) to the six stages used for RNA-seq (Fig. 1b and c, Table S7). Larval and adult stages showed a distinct genome-wide chromatin accessibility landscape (Figure S8a and b). 165,946 open chromatin regions were detected across all sampled stages, with on average 12.9%, 51.5% and 29.7% overlapping promoter (−2 kb + 0.5 kb transcription start site; TSS), genic (+ 0.5 kb TSS to transcription end site; TES) and intergenic regions, respectively (Figure S8c), consistent with the previous study in the oyster (Xu et al. 2023). There was, however, an increase in promoter peaks in adults compared to larvae (Figure S8d). Moreover, we observed that adult mantles exhibited more differentially accessible regions relative to the D-shape larvae than to other larval stages, suggesting the existence of distinct cis-regulation programs between these two stages (Figure S8e).

Based on these observations, we selected D-shape larvae and adult mantles, respectively, as representative samples for larval and adult shell formation, and performed cleavage under targets and tagmentation (CUT&Tag) profiling for three types of histone modification H3K4me3, H3K27ac, and H3K27me3 (Fig. 1b and c, Table S8). As expected, H3K4me3 peaks were highly enriched near gene TSSs and most abundant within promoter regions, whereas H3K27ac and H3K27me3 marks were more broadly located across genes, with enrichment towards the TSS (Figure S9a and b). We also found that genes with relatively high expression levels were, on average, marked by more H3K27ac and H3K4me3, and less H3K27me3, except for H3K27me3 in D-shape larvae (Figure S9c and d). The positive correlation between H3K27me3 and gene expression in larvae may reflect high cellular heterogeneity or unresolved long-range regulatory interactions in the larval samples. Differential gene expression between D-shape larvae and adult mantles was significantly and positively correlated with changes in H3K4me3 and H3K27ac levels (Pearson correlation coefficient R = 0.724 and 0.422, respectively; *P* < 2.2e−16) (Figure S9e-g).

**Figure 3:**
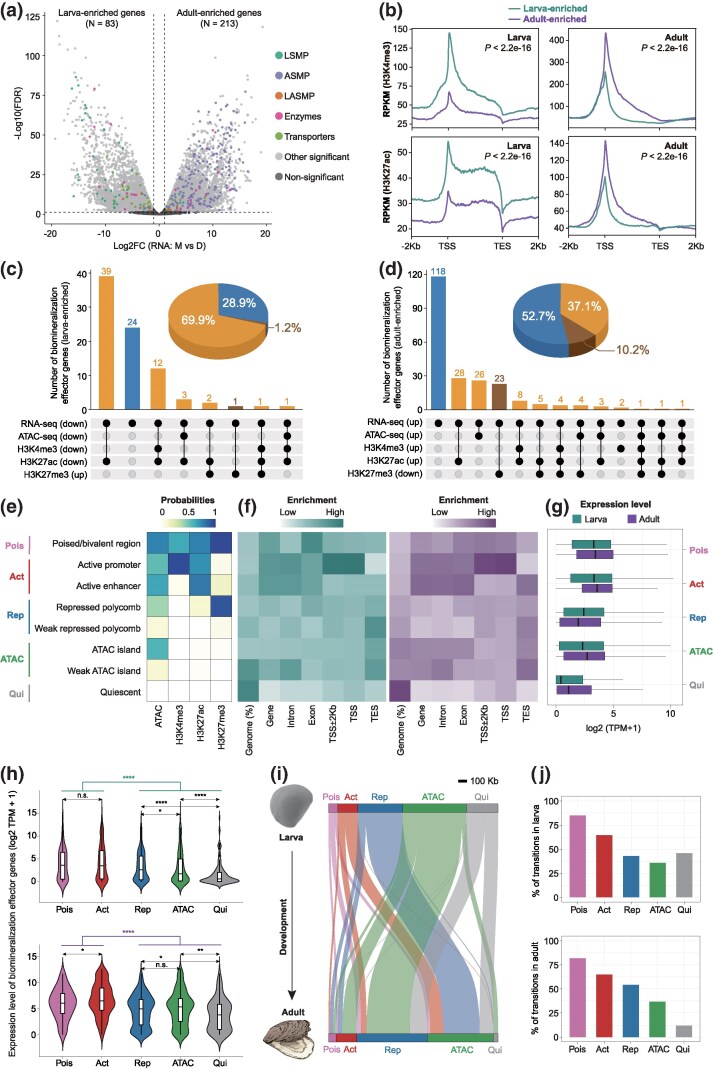
Dynamic remodeling of chromatin states orchestrates the regulation of biomineralization effector genes during larval and adult shell formation. a) Differential gene expression between D-shape larvae and adult mantles, with biomineralization effectors highlighted dots in color. Genes significantly up-regulated in adult mantles were defined as adult-enriched, while those down-regulated were considered as larva-enriched. Detailed information is shown in Table S6. b) H3K4me3 and H3K27ac levels in genomic regions containing larva-enriched or adult-enriched genes, shown for larval (left) and adult (right) stages. Statistical significance of histone modification levels around larva-enriched or adult-enriched genes (from 5 kb upstream to the TES) was assessed using a two-sided Wilcoxon rank-sum test. c) Upset plots showing the overlap between larval-enriched biomineralization effector genes and corresponding chromatin features (from 5 kb upstream to the TES), characterized by significantly increased chromatin accessibility and active histone modifications (H3K4me3 and H3K27ac) (yellow) in the adult, compared to larvae, accompanied by a decrease in the repressive mark H3K27me3 (brown). Pie charts depict the proportion of larva-enriched genes with differential chromatin features versus those without (blue). Detailed information is shown in Table S9. d) Upset plots showing the overlap between adult-enriched biomineralization effector genes and corresponding chromatin features (from 5 kb upstream to the TES) that exhibit significantly decreased chromatin accessibility and active histone modifications (H3K4me3 and H3K27ac) (yellow) in the adult, relative to those of larvae, along with repressive mark H3K27me3 increasing (brown). Pie charts show the proportion of adult-enriched genes with differential chromatin features compared to those without (blue). Detailed information is shown in Table S9. e) ChromHMM eight chromatin state models grouped into five categories: poised (Pois), active (Act), repressed (Rep), accessible chromatin (ATAC), and quiescent (Qui) states, based on ATAC-seq and histone modification profiles. f) Genomic feature enrichments for each chromatin state at larval and adult stages. g) Expression levels of genes with each chromatin state across both stages. Error bars represent the median with quartiles. h) Gene expression levels of biomineralization effectors with each chromatin state at larval (top) and adult (bottom) stages. Error bars represent the median with quartiles. Two-sided Wilcoxon tests were used for comparisons between states (*P < 0.05; **P < 0.01; ****P < 0.0001; n.s., no significance). i) Sankey diagram showing chromatin state transitions of biomineralization effector genes from larva (top) to adult (bottom) stages. j) Top: percentage of chromatin states linked to biomineralization effector genes in larvae that undergo transitions to other chromatin states in adults. Bottom: percentage of chromatin states linked to biomineralization effector genes in adults that undergo transitions from other chromatin states in larvae.

We next identified adult-enriched (highly expressed in adult mantles) and larva-enriched (highly expressed in D-shape larvae) genes based on differential expression analysis (|log_2_foldchange| > 1, false discovery rate corrected *P*-value (FDR) < 0.05) (Fig. 3a). Both larva- and adult-enriched genes followed similar trends of H3K4me3 and H3K27ac enrichment at TSS, consistent with their roles as canonical markers of transcriptional activation. Notably, these modifications showed stage-specific patterns, with larva- and adult-enriched genes associated with high levels of both H3K4me3 and H3K27ac during the larval and adult stages, respectively (*P* < 2.2e−16) (Fig. 3b). In total, 83 and 224 biomineralization effector genes were identified as larva and adult-enriched genes, respectively (Fig. 3a). Among them, 59 (71.1%) larva-enriched and 106 (47.3%) adult-enriched biomineralization effector genes correlated with significant changes in either chromatin accessibility or at least one histone mark (Fig. 3c and d, and Table S9). Importantly, the majority of these genes exhibited regulatory changes linked to open chromatin regions, H3K4me3 or H3K27ac, accounting for 69.9% of larva-enriched genes and 37.1% of adult-enriched genes. These findings imply H3K4me3 and H3K27ac as critical histone marks for transcriptional activation during larval and adult shell formation.

The chromatin states of larvae and adults were systematically characterized using ChromHMM (Ernst and Kellis 2017), which integrated combinatorial patterns of multiple epigenomic marks (Figure S10a). For simplicity, the eight identified chromatin states were consolidated into five major categories used throughout this study (Fig. 3e), according to previous studies (Zhou et al. 2011; Ali et al. 2024). Genomic regions marked by accessible chromatin, H3K4me3, and H3K27ac were classified as active promoters, typically showing biased distribution around the TSS (Fig. 3f), whereas those enriched for open chromatin and H3K27ac, but lacking H3K4me3, were categorized as active enhancers. Together, these regions were defined as activate elements (Act). Regions characterized by high H3K27me3 levels and low levels of chromatin accessibility were classified as repressed elements (Rep), while those marked by both H3K4me3 and H3K27me3 features were defined as poised/bivalent states (Pois). Regions marked exclusively by open chromatin were classified as accessible islands (ATAC), and those with no epigenomic signals were quiescent states (Qui). As expected, poised and active elements (Pois and Act) showed high gene expression levels (Fig. 3g). In contrast, repressed (Rep) and the low signal regions (Qui) had relatively low gene expression levels (Fig. 3g). Furthermore, biomineralization effector genes linked to poised and active states (Pois and Act) exhibited significantly higher expression levels compared to those remaining in other states (Rep, ATAC and Qui) in both larval and adult stages (Fig. 3h). Effector genes associated with active promoters and enhancers (Act) showed the most significant differences (*P* = 2.7e−16) in expression levels between larvae and adults, indicating a regulatory role of these elements in larval and adult shell formation. We next investigated epigenomic transitions from larval to adult stages and found widespread switching among chromatin states across the genome (Figure S10b and c). Focusing on regulatory elements associated with biomineralization effector genes, a large proportion of poised (84.8%) and active (64.4%) regions in larvae transitioned to alternative chromatin states in adults, whereas most poised (81.8%) and active (65.0%) regions observed in adults originated from other chromatin states in larvae (Fig. 3i and j). These results indicated that oyster regulatory programs for shell formation were extensively rewired between life stages, with regulatory elements associated with biomineralization effector genes selectively activated to meet stage-specific requirements of larval or adult shell formation. Such dynamic remodeling establishes novel chromatin landscapes that enable the activation of adult-specific biomineralization programs.

### Functional divergence of biomineralization paralogs for larval and adult shell formation

**Figure 4:**
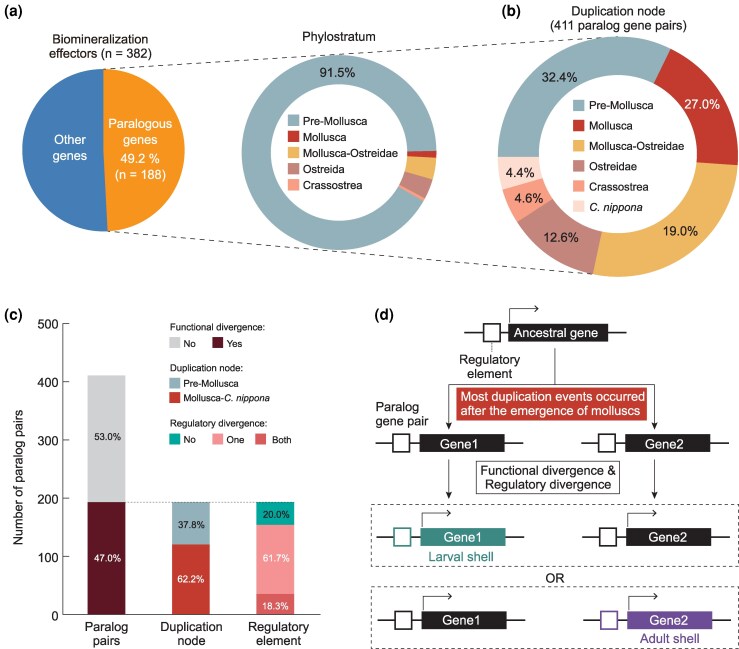
Functional specialization of biomineralization paralogs in larval or adult shell formation. a) Percentage of paralogous genes in biomineralization effector genes, and the distribution of their phylostrata. b) Duplication of nodes of paralog gene pairs. c) Percentage of paralog gene pairs with functional divergence, and the distribution of duplication nodes and regulatory element divergence among them. d) Schematic representation of paralog specialization following gene duplication. After a mollusc-specific duplication from an ancestral gene, one paralog retained the ancestral regulatory program and function, while the other acquired distinct regulatory elements and functions, driving specialization for larval or adult shell formation.

Gene duplication is a major driver of molecular innovation in shell biomineralization, facilitating the emergence of novel or specialized functions among paralogous biomineralization genes in molluscs (Sarashina et al. 2006; Zhao et al. 2020; Bai et al. 2023), often accompanied by changes in cis-regulatory elements (Shimizu et al. 2022). To explore the evolutionary origins and divergence times of the paralogous genes involved in larval and adult shell formation, we identified 188 paralogous genes encoding biomineralization effectors using phylogenetic inference (Fig. 4a and Table S10). Phylostratigraphic analysis (Barrera-Redondo et al. 2023) revealed that most (91.5%) of these genes originated before the emergence of molluscs (pre-molluscan origin), whereas their paralog duplication events occurred predominantly at or after the origin of molluscs, thus representing mollusc-specific gene expansions for shell formation (Fig. 4b, Figure S11a, Table S10). In addition, this phenomenon was also observed in other molluscan species (Figure S11b and Table S11). To investigate the functional specialization of biomineralization effector genes between larval and adult shell formation, we classified these biomineralization effector genes into three groups (Table S10), based on differential expression analysis between larval and adult stages in *C. nippona* (Fig. 3a). Larva-enriched and adult-enriched biomineralization effector genes were respectively involved in larval and adult shell formation, whereas genes without significant differential expression likely function in both stages (Table S10). This stage-specific expression framework provides a valuable basis for dissecting the developmental and regulatory mechanisms underlying shell formation. Interestingly, 193 (47.0%) of the analyzed paralog gene pairs exhibited divergent expression patterns between larval and adult stages, indicating functional divergence. Of these, 62.2% resulted from duplication events specific to molluscan lineages (Fig. 4c and Table S10), suggesting that the functional divergence of biomineralization genes between larval or adult shell formation may represent molecular innovation during mollusc evolution.

To further assess the underlying regulatory basis for this functional divergence, we investigated active chromatin marks (H3K4me3 and H3K27ac) associated with each paralog in larvae and adults. The regulatory divergence among functionally specialized paralog pairs was categorized into three types: no divergence (both genes sharing similar regulatory activity across stages), divergence in one gene of the pair, and divergence in both genes (Table S10 and Note S3). Strikingly, among paralog pairs that had already diverged in expression patterns, the majority (61.7%) displayed regulatory divergence in a single paralog, while few fraction (18.3%) showed divergence in both genes (Fig. 4c). This asymmetric regulatory evolution suggests that, in many cases, one paralog retains the ancestral regulatory program and potentially the ancestral function, while the other acquires novel stage-specific regulatory elements, such as active elements only in larval or adult tissues, leading to functional innovation (Fig. 4d). Such one-sided acquisition of regulatory activity may facilitate rapid neofunctionalization, as the newly specialized paralog can adopt novel roles without compromising the original function preserved in its counterpart. These findings indicate that changes in cis-regulatory architecture, particularly the stage-specific gain of active chromatin regions in a single paralog, are the major mechanism driving the functional specialization of biomineralization effector genes during molluscan evolution.

### Inferred regulatory networks supporting conserved TFs in larval and adult shell formation

**Figure 5:**
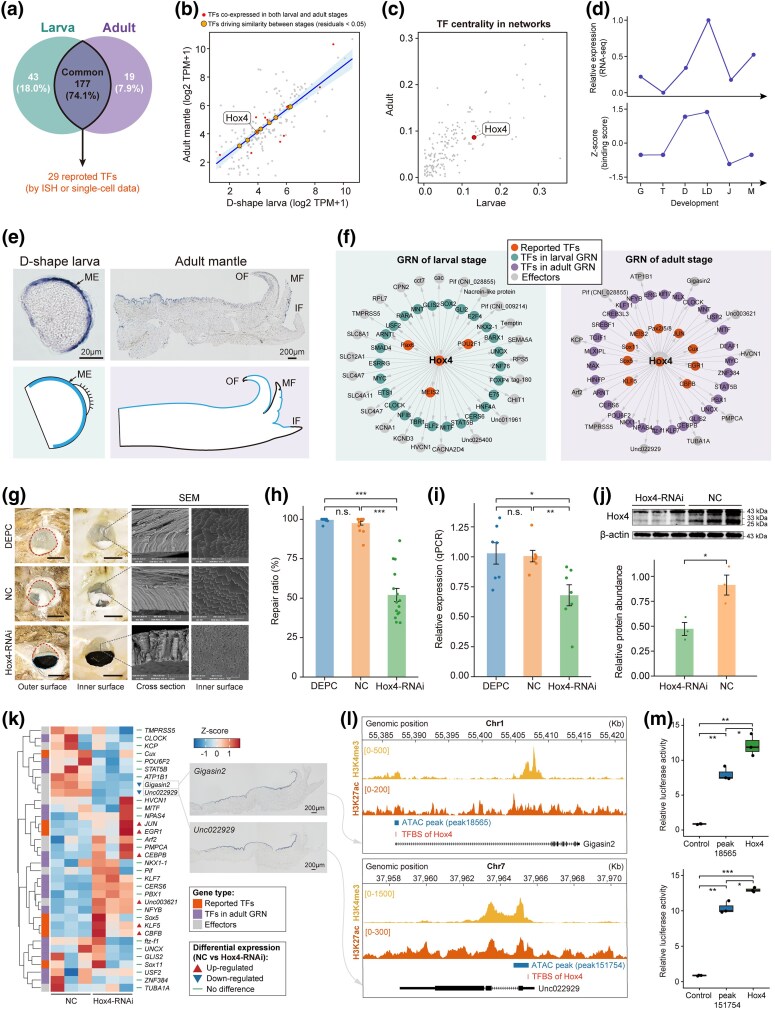
Conserved biomineralization TFs regulating both larval and shell formation in oysters. a) Venn diagram showing putative biomineralization TFs in larval (green) and adult (purple) shell formation GRNs, with 29 reported TFs (orange) conserved across stages. b) Scatter plot of gene expression of putative biomineralization TFs between D-shape larvae and adult mantles. A weighted least squares (WLS) regression model accounts for heteroscedasticity. The blue line indicates the ordinary least squares regression fit. Orange points represent candidate TFs with consistent expression relationships between stages (|residual| < 0.05). Red points highlight co-expressed TFs from Figure S15h. c) TF centrality in larval (*x* axis) and adult (*y* axis) GRNs, with *Hox4* highlighted in red. d) Expression and binding scores of *Hox4* across six stages/tissues. Abbreviations are the same as Fig. 1b. e) ISH (top) and schematic (bottom) showing *Hox4* expression in D-shaped larvae and adult mantle. Negative controls of ISH were shown in Figure S16. ME, mantle edge; OF, outer fold; MF, middle fold; IF, inner fold. f) GRNs showing *Hox4*-regulated biomineralization genes in larvae (left) and adults (right). g) Shell repair after drilling, in control (DEPC and NC) and *Hox4*-RNAi groups at day 6. Left: representative bright-field photographs showing drill holes (red dashed) and unrepaired regions (blue dashed). Scale bar = 2 mm. Right: SEM images showing cross-sections (grey solid lines) through the repaired shell layers and the inner surface of the repaired shells (foliated layer) in the regions surrounding the grey solid lines. Note the irregular and disorganized foliated layer in the *Hox4*-RNAi group compared with the orderly structure in controls. h) Shell repair ratio (repaired/hole area) in each group (n = 15; mean ± SE; two-sided Student's *t*-test: ***P < 0.001; n.s., no significance). Full images are shown in Figure S17. i) *Hox4* expression in mantle tissues following RNAi experiments (n = 7; mean ± SE; two-sided Student's *t*-test: *P < 0.05; **P < 0.01; n.s., no significance). j) Western blot analysis of Hox4 protein abundance in mantle tissues following RNAi experiments (n = 3; mean ± SE; two-sided Student's *t*-test: *P < 0.05). k) Heatmap showing expression of *Hox4*-regulated downstream biomineralization genes in the adult GRN after RNAi (n = 3). Differential expression is indicated beside each gene name: upward red triangles denote significantly up-regulated genes after RNAi (log_2_foldchange > 1 and FDR < 0.05); downward blue triangles denote significantly down-regulated genes (log_2_foldchange < −1 and FDR < 0.05); and green horizontal bars indicate genes without significant changes (|log_2_foldchange| < 1 or FDR ≥ 0.05). Detailed data are shown in Table S14. Spatial expression patterns of two down-regulated genes in adult mantles are shown on the right. Negative controls are shown in Figure S18b to d. l) Regulatory activity around *Gigasin2* (top) and *Unc022929* (bottom), where regulatory elements located at 5' UTRs contain predicted *Hox4* binding sites (TFBS). m) Dual-luciferase assays validating *Hox4*-mediated activation of *Gigasin2* (top) and *Unc022929* (bottom) in human 293T cells (n = 3; two-sided Student's *t*-test: *P < 0.05; **P < 0.01; ***P < 0.001). Control: pGL3-basic; Peak: pGL3 + peak; Hox4: pGL3 + peak + *Hox4*.

The coordination between transcription and active chromatin landscape suggests that cis-motif and trans-factors mediating GRNs could govern shell formation. To bridge TFs and target motifs, we identified 1,123 TFs genome-wide in *C. nippona* and assigned 312 of them to transcription factor binding sites (TFBSs) derived from the JASPAR database (Table S12). By integrating the ATAC-seq footprinting (Bentsen et al. 2020) and analysis algorithm for networks specified by enhancers (ANANSE) (Xu et al. 2021) with predicted cis-motifs, we then constructed the GRNs for larval and adult shell formation (Figure S12a). Two independent networks, one for larvae and one for adults, were generated, consisting of nodes (TFs and effectors) and edges (the interactions between TFs and target genes) (Figure S12b and Table S13). In total, 239 TFs were identified, with 220 and 196 upstream TFs present in the larval and adult networks, respectively (Fig. 5a and Figure S12b). Notably, a total of 177 TFs (74.1% of all putative TFs) were common to both networks, including 29 TFs previously validated by ISH or single-cell RNA-seq (Fig. 5a and Table S4). These shared TFs were expressed during both larval and adult stages, with no significant differences between larvae and adult mantles (Figure S13a and b). By contrast, TFs specific to the larval or adult GRNs exhibited higher expression levels during the corresponding stages (Figure S13c). Together, these results support that a largely conserved set of upstream TFs orchestrates distinct transcriptional programs for larval and adult shell formation in the oyster.

We reasoned that the diversification of larval and adult shells might be attributed to the duplication and functional divergence of paralogous genes during molluscan evolution. However, phylostratigraphic analysis revealed that all upstream putative biomineralization TFs are of pre-molluscan origin, with their paralog duplication events predominantly occurring prior to molluscan emergence (Figure S13d and e). Thus, biomineralization TFs are likely evolutionary ancient components that were co-opted to regulate recently evolved effector genes for molluscan shell formation. Moreover, GO enrichment analysis revealed that TFs in the two GRNs were not only enriched for terms related to skeletal system development and morphogenesis, but also for body plan pattern specification, cell fate determination, and other ancient early developmental processes (Figure S14). To explore the potential co-option of early developmental TFs into shell formation, we investigated the transcriptional similarity of common TFs between the D-shape larval and adult stages (Fig. 5b). Given that these TFs regulated distinct transcriptional programs in larval and adult shell, we also performed weighted correlation network analysis (WGCNA) (Langfelder and Horvath 2008) separately on developmental stages and adult tissues, identifying 24 co-expressed TFs (Figure S15). Notably, we observed that Hox4 was among the set of TFs with conserved expression across both D-shape larva and adult stages (Fig. 5b and Figure S15h), and showed mantle-specific expression among adult tissues (Figure S15g). Hox4 was also one of the most central TFs in larval and adult shell formation GRNs (Fig. 5c). Consistent with its previously reported role in early axial patterning and larval shell formation in molluscs (Huan et al. 2020), *Hox4* was overrepresented and upregulated on the basis of its binding score at the D-shape larva stage (Fig. 5d), and was spatially expressed in the mantle edge of D-shape larvae and epithelial cells of the adult mantle in *C. nippona* (Fig. 5e and Figure S16). In addition, *Hox4* was predicted to regulate distinct sets of downstream effector genes and TFs in larvae and adults, which were involved in the larval and adult shell formation, respectively (Fig. 5f).

To further confirm the function of *Hox4* in shell formation, we performed RNA interference (RNAi) experiments in adult oysters during shell repair. After drilling holes in the left valve followed by shell repair for 6 days, the inner surfaces of foliated layer of the repaired shells showed irregular growth in the *Hox4*-RNAi groups, compared with those of control groups (DEPC and NC groups) (Fig. 5g). Furthermore, the *Hox4*-RNAi oysters exhibited a significantly lower shell repair ratio than the control oysters (*P* < 0.001) (Fig. 5h and Figure S17). Both gene expression levels and protein abundances of *Hox4* were also significantly decreased in the mantles of *Hox4*-RNAi oysters compared with those of controls (*P* < 0.05) (Fig. 5i and j). Transcriptomic analysis of the oyster mantles after RNAi experiments revealed a significant down-regulation of two downstream SMP genes (*Gigasin2* and *Unc022929*) in the *Hox4*-mediated adult GRN (Fig. 5k and Table S14). These genes were expressed in the epithelial cells of the adult mantle in *C. nippona* and potentially associated with shell formation (Fig. 5k and Figure S18). In contrast, most larval GRN effector genes did not exhibit significant expression changes after RNAi (Figure S19 and Table S14), indicating that the larval effector module was largely inactive and unresponsive to *Hox4* knockdown in adults. Finally, dual-luciferase reporter assay further validated that *Hox4* regulates *Gigasin2* and *Unc022929* by binding to their promoter regions (Fig. 5l and m, Table S15). Overall, these results suggest that the ancient *Hox4* gene, with biological functions related to early development (Huan et al. 2020; Hombria et al. 2021; Peraldi and Kmita 2024), has been co-opted for the oyster shell formation by regulating the expression of downstream SMP genes.

### A conserved TF toolkit for larval exoskeleton formation in lophotrochozoans

**Figure 6:**
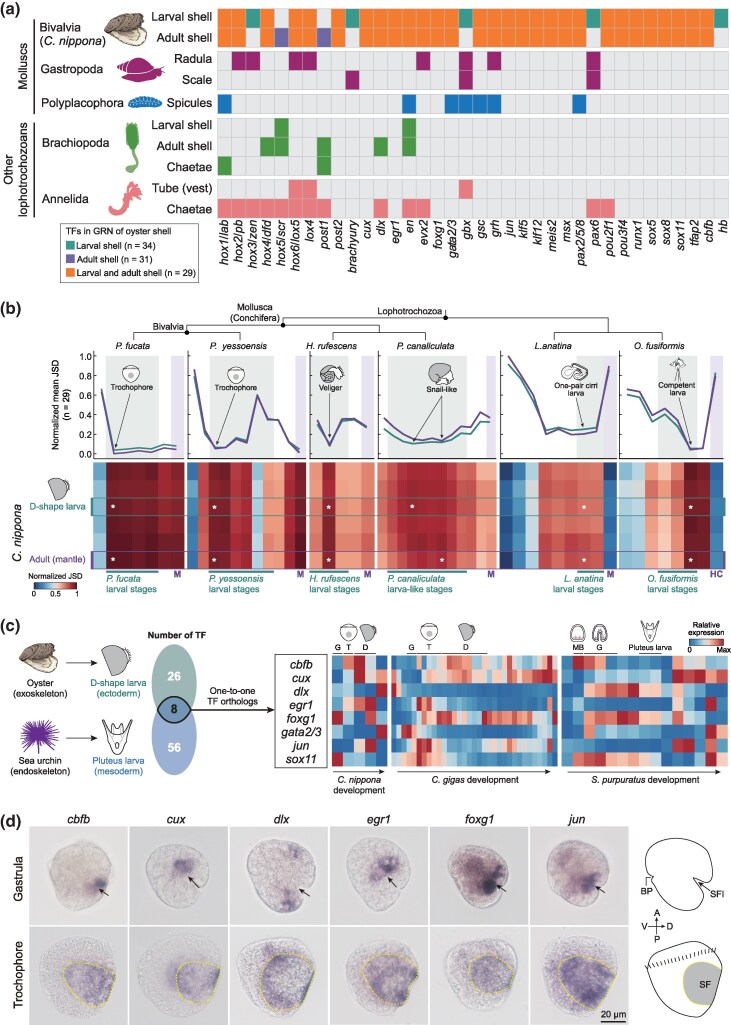
A biomineralization toolkit of TFs in lophotrochozoan larvae, with co-option for endoskeleton development in echinoderms. a) TFs likely involved in biomineralized structure formation across major lophotrochozoan lineages. Colored squares indicate the recruitment of a TF into the GRN for each structure, while grey squares denote absence. TFs associated with only larval shell formation (green), only adult shell formation (purple), or both stages (orange) in Bivalvia (*C. nippona*) are compared to those involved in other molluscan structures, including the radula and scale in gastropods (dark magenta) and the spicules in polyplacophorans (blue), as well as exoskeletons in other lophotrochozoans, namely the larval and adult shells and chaetae of brachiopods (medium green) and the tube (vest) and chaetae of annelids (salmon). Detailed data are shown in Table S16. b) Heatmaps of pairwise normalized JSD values of 29 orthologous TFs between *C. nippona* and other lophotrochozoans (*P. fucata*, *P. yessoensis*, *H. rufescens*, *P. canaliculate*, *L. anatina*, and *O. fusiformis*) across life stages. Lower values indicate higher expression similarity. Asterisks mark stages of minimal divergence of each species to *C. nippona* D-shape larvae (green) and adult mantle (purple); corresponding average relative JSDs are shown above each heatmap. Fully labelled heatmaps are shown in Figure S21a. M, adult mantle; HC, head and chaetae. c) Comparison of TF orthologs (left) between the exoskeleton GRN of D-shape oyster larvae and endoskeleton GRN of pluteus-stage sea urchin larvae. Right: developmental expression patterns of these TFs in *C. nippona* (from gastrula to D-shape larvae and juvenile), *C. gigas* (from rotary movement to juvenile), and *S. purpuratus* (from cleavage to juvenile). d) Spatial expression patterns of six conserved putative biomineralization-related TFs in the gastrula and trochophore of *C. nippona*. Whole-mount ISH results (lateral view) showed expression covering the ectodermal shell field invagination (SFI) during gastrulation and the shell field (SF) in the trochophore. Arrows indicate the SFI. Yellow dashed lines indicate the SF region in the trochophore. Right: schematic diagrams of gastrula (top) and trochophore (bottom) in lateral view. Negative controls and dorsal views are shown in Figure S24. A, anterior; P, posterior; V, ventral; D, dorsal; BP, blastopore.

Among the 239 putative biomineralization TFs in GRNs regulating shell formation of *C. nippona* (Fig. 5a and Figure S12), 36 putative one-to-one orthologs of these TFs were also supported by published experimental data for shell formation in other molluscs (Tables S4 and S16). The 36 TFs were further compared with those known to regulate the formation of other biomineralized or hard structures in lophotrochozoans (Fig. 6a and Table S16). Several orthologs of these TFs contribute to the formation of other molluscan biomineralized products, including gastropod radulae and scales, as well as chiton spicules. Moreover, 14 orthologous TFs reported to date from other lophotrochozoan exoskeletons, such as brachiopod shells and chaetae, and annelid tubes and chaetae, were found to be involved in oyster shell formation, with nine of them for both larval and adult shell formation in *C. nippona*. The widespread recruitment of putative orthologous TFs across diverse biomineralized exoskeletons and other hard structures suggests the potential existence of a conserved biomineralization toolkit derived from the ancestral lophotrochozoan genome (Sun et al. 2020, 2021; Barrera Grijalba et al. 2025).

Next, we investigated whether putative orthologs for the 29 TFs commonly involved in both larval and adult shell formation in *C. nippona* also contribute to the formation of biomineralized exoskeletons across the life cycle of other lophotrochozoans (Fig. 6a). Orthologs of these TFs were identified in seven molluscs and three other lophotrochozoans with representative biomineralized exoskeletons and available developmental or tissue-specific transcriptome data, as well as in the ecdysozoan *Amphibalanus amphitrite* as an outgroup (Table S17). Indeed, the overall expression dynamics of these 29 genes were largely consistent across lophotrochozoans, with high expression levels at early exoskeleton-forming larval stages (trochophore or D-shape larva in bivalves, veliger in gastropods, one-pair cirri larva in the brachiopod *Lingula anatina*, and mitraria larva in the annelid *Owenia fusiformis*) and adult skeletogenic tissues (Figure S20). In contrast, these genes exhibited comparatively lower expression in the tube-forming organs (collar and opisthosoma) of the tubeworm *Paraescarpia echinospica*, consistent with their low expression in the mantle of *A. amphitrite*. To obtain a comparative view of transcriptional dynamics of these TFs in lophotrochozoans, we calculated the pairwise Jensen-Shannon divergence (JSD) index of gene expression similarity between *C. nippona* and other lophotrochozoans, respectively (Fig. 6b, Figures S21 and S22). The early shell-forming larval stages of other lophotrochozoans showed the highest similarity with the D-shape larval stage *C. nippona*, thus appearing as a mid-developmental transition between two phases of higher transcriptional dissimilarity, namely the embryonic stage and the later larval stage (Fig. 6b and Figure S21). These results further support the hypothesis of a shared TF toolkit present in the early larval development of lophotrochozoans, regulating the formation of the biomineralized exoskeleton. Notably, gene expression divergence decreased after metamorphosis of molluscs, particularly in bivalves, but increased in adult skeletogenic organs of non-molluscan lophotrochozoans. Additionally, the adult mantle of *C. nippona* showed higher expression similarity with those of other molluscs, compared to the skeletogenic organs of non-molluscan lophotrochozoans (Figure S22). These findings suggest that the temporal co-option of the 29 TFs into larval and adult shell formation probably represents an evolutionary innovation in molluscs.

### Independent co-option of biomineralization TFs driving the convergence of exoskeletons and endoskeletons in larvae

To assess whether the transcriptional dynamics of biomineralization TFs found in the biomineralized exoskeleton of lophotrochozoan larvae are also present in endoskeletons of other bilaterian larvae, we reconstructed the skeletogenic GRN of early pluteus larvae in the sea urchin *Strongylocentrotus purpuratus* using previously published datasets (Paganos et al. 2021; Arenas-Mena and Akin 2023) (Figure S23a and Tables S18 and S19). A total of 64 putative biomineralization TFs were identified in pluteus larvae (Table S20). Transcriptomic analysis across developmental stages in *S. purpuratus* revealed that these genes are highly expressed during early skeletogenic phases, particularly at gastrula stages, and in adult spines (Figure S23b), suggesting their potential roles in skeletal development and biomineralization. However, the expression of these genes showed no significant differences among adult tissues (Figure S23c), implying that these TFs may play more critical roles during early development of the endoskeleton rather than in adult skeletal maintenance. Moreover, a comparative analysis of transcriptomic similarity throughout the entire development of *S. purpuratus* and another sea urchin species, *Lytechinus variegatus*, demonstrated that the gastrula stage exhibits the highest transcriptional similarity in the life cycles of sea urchins, followed by the pluteus larva stage (Figure S23d and Table S20). Similarly, when comparing *S. purpuratus* with the sea cucumber *Apostichopus japonicus*, another echinoderm that forms biomineralized spicules but lacks a substantial endoskeleton during larval development (Zhang et al. 2017), the blastula is the period of maximal transcriptomic similarity (Figure S23e), corresponding to a skeletogenic mesenchyme in sea cucumbers (McCauley et al. 2012). These findings suggest that larval skeletogenic mesenchyme formation in echinoderms may be regulated by evolutionarily conserved biomineralization TFs, possibly through heterochronic activation of ancestral adult skeletogenic programs (Gao and Davidson 2008; Erkenbrack and Thompson 2019).

We further extended our comparisons of putative biomineralization TFs between D-shape larvae of the oyster *C. nippona* and pluteus larvae of the sea urchin *S. purpuratus*, and identified only eight putative orthologous TFs shared between the two species (Fig. 6c). Although these genes are associated with early skeletal development in both organisms, their temporal expression profiles differed markedly. Most of them were highly expressed during trochophore or D-shape larval stages in oysters, whereas they exhibited high expression predominantly in the mesenchyme blastula and gastrula stages of the sea urchin (Fig. 6c). This result indicates a potential heterochronic shift in the deployment of these biomineralization TFs, reflecting lineage-specific timing in the activation of skeletogenic GRNs. In addition to temporal divergence, the embryonic origin of skeletogenic cells also differs fundamentally between molluscan and echinoderm larvae. The shell field forms within the dorsal ectoderm in oyster larvae and later develops into the mantle for shell formation, whereas sea urchin spicules are produced by mesenchymal skeletogenic cells (Wilt 2005). All eight TFs exhibited spatial expression patterns that covered the shell field of trochophore in oysters, as supported by previous studies (Liu et al. 2015, 2017) and our ISH results (Fig. 6d and Figure S24). These developmental differences underscore that, despite limited conservation of regulatory TFs, the complexity and morphogenetic mechanisms of skeletal formation have diverged substantially between molluscs and echinoderms. Overall, the convergent evolution of larval exoskeletons in molluscs and larval endoskeletons in echinoderms likely reflects the independent co-option of a restricted set of ancestral TFs into distinct embryonic and developmental contexts.

## Discussion

The evolutionary origin of biomineralized exoskeletons in lophotrochozoans is a topic of ongoing debate (Luo et al. 2015; Shimizu et al. 2017; Zhao et al. 2017; Li et al. 2018; Murdock 2020; Shore et al. 2021; Wernstrom et al. 2022; Sleight 2023). Current hypotheses assume that the common ancestor of lophotrochozoans possessed non-mineralized structures that were independently biomineralized in each lineage (Luo et al. 2015; Shimizu et al. 2017; Zhao et al. 2017; Li et al. 2018; Murdock 2020), while some have argued that the biomineralized exoskeleton is an ancestral lophotrochozoan trait (Shore et al. 2021; Wernstrom et al. 2022). The construction of GRNs offers a powerful framework to uncover the evolutionary history and hierarchical logic underlying this complex biological process, whereby upstream regulators coordinate the activation of intermediate modules and downstream effectors. However, to date, systematic molecular characterization of biomineralization GRNs in lophotrochozoans remains scarce, with existing studies mostly focusing on the spatial expression patterns of TFs and effector genes (Luo et al. 2015; Shimizu et al. 2017; Wernstrom et al. 2022). In this study, our comprehensive profiling of multi-stage transcriptomes and epigenomes in *C. nippona* provided a novel perspective on the establishment of shell formation GRNs in molluscs and revealed a biphasic regulatory program orchestrated by a set of conserved TFs that govern distinct processes of molluscan shell formation across life stages. Surprisingly, comparative analyses of transcriptional dynamics suggested a high overall similarity in larval regulatory modules during lophotrochozoan skeletogenesis (Fig. 6a and b), reflecting an ancient and conserved regulatory toolkit for larval exoskeleton formation in lophotrochozoans. Although the exact timing remains uncertain, this conservation indicated that the GRNs of larval exoskeletons probably originated prior to the divergence of molluscs, brachiopods, and annelids, even tracing back to the common ancestor of lophotrochozoans. However, the adult molluscan shell appears to represent a phylum-specific evolutionary innovation. The rapid evolution of downstream effector genes and lineage-specific functional divergence of paralogs can be proposed to have supplied the genetic foundation underlying the remarkable diversity of adult shells, facilitated by dynamic chromatin remodeling and heterochronic deployment of largely conserved upstream TFs shared with the larval regulatory program. This model is not only consistent with previous studies showing the extensive incorporation of novel or evolutionarily young genes into the shell gland cell and mantle transcriptomes of molluscs (Wang et al. 2020; Piovani et al. 2023) but further highlights the pivotal roles of epigenetic reprogramming in modulating GRNs underlying shell biomineralization across life stages (Xu et al. 2023). The conserved TF architecture observed across multiple bivalve and gastropod species indicates that the regulatory logic uncovered here is likely a mollusc-wide feature rather than a bivalve-specific peculiarity. Future comparative developmental and epigenomic analyses across other lineages will be essential to refine the timing, prevalence, and origins of biomineralization GRNs.

Although a recent hypothesis proposed that non-skeletal micro-biominerals, such as statoliths, might have served as precursors of lophotrochozoan skeletons (Sleight 2023), our reconstructed GRNs are tightly linked to exoskeleton-forming tissues and do not directly support this intermediate scenario. While expression data from *Crepidula* indicated that some SMPs are shared between larval shell fields and statocysts (Lopez-Anido et al. 2024), these SMPs likely reflect lineage-specific co-option of effector genes for larval shell and statolith formation within *Crepidula*, rather than an ancestral biomineralization program shared by the two structures. Evaluating the statolith-precursor scenario will require more data and broader comparative analyses across diverse lophotrochozoan lineages.

Epigenetic transitions also appear to play crucial roles in the development and shell formation of oysters and other bivalves. DNA methylation dynamics accompany cleavage and metamorphosis of oysters, which are associated with transcriptional programs at these transitions (Riviere et al. 2013, 2017). In addition, JmjC-domain histone demethylases reshape H3 methylation landscapes and thereby influence larval developmental trajectories and stress resilience in oysters (Fellous et al. 2015). A conserved and functional m⁶A RNA methylation machinery further contributes to early patterning and the maternal-to-zygotic transition of oysters (Le Franc et al. 2021, 2023). Collectively, methylation has been implicated in early development and major life-history transitions where biomineralization GRNs are established and extensively reconfigured. By contrast, genomic and epigenomic analyses in the Antarctic clam (*L. elliptica*) showed that shell damage responses involved major transcriptional reprogramming, but limited involvement of DNA methylation, suggesting other epigenomic mechanisms underlie long-term physiological reprogramming in adult bivalves (Sleight et al. 2025). Our ATAC-seq and histone mark profiles complement existing methylation-based studies by resolving cis-regulatory element accessibility and chromatin state transitions across life stages, thereby linking epigenomic landscapes to the deployment of biomineralization GRNs and offering a foundation for future comparative epigenomic analyses across molluscs.

A critical unresolved challenge in GRN construction is that computationally inferred TF-target links remain hypothetical until functionally validated (Kim et al. 2023). A direct way to test these predicted edges is to perturb the TF and measure the resulting changes in target gene expression (Streit et al. 2013; Datlinger et al. 2017; Feng et al. 2020). In this study, *Hox4* knockdown provided a relatively early and partially penetrant perturbation of adult biomineralization GRN, impairing both the efficiency of shell repair and shell microstructure. Nevertheless, longer-term experiments will be required to fully characterize the final maturation and stability of the repaired shell. Despite *Hox4* emerging as a network hub, its knockdown did not completely abolish shell repair, and a biomineralized repair membrane still formed. This graded phenotype is consistent with partial knockdown rather than a true null, as observed in other systems (Wang et al. 2024). At the transcriptional level, the reduction of *Hox4* expression resulted in the expected down-regulation of a subset of adult effector genes, but also up-regulation of five downstream TFs and one SMP effector within the adult GRN (Fig. 5k). Such responses indicate that *Hox4* likely functions both as an activator and a repressor, and that its depletion de-represses a subset of targets, as reported for other central TFs (Harada and Burton 2020; Voronov et al. 2024). In addition, residual *Hox4* activity and compensation by parallel or cooperating regulators may further buffer the network, preserving a reduced but still measurable repair response, which was also observed in perturbations of multilayered GRNs (Gazdag et al. 2016). Likewise, most larval GRN effectors showed no response in the adult mantle after *Hox4* RNAi, although two genes, including a TF and an SMP, also present in the adult GRN, were affected (Figure S19 and Table S13). This suggests that larval and adult GRNs are not entirely segregated, instead sharing a small effector subset that can be co-opted or indirectly influenced through cross-regulation among adult regulatory modules. Taken together, TF knockdown combined with bulk RNA-seq provides a valuable but preliminary and tissue-averaged functional assay for GRN validation. More powerful and precise functional tools, including more complete and temporally controlled perturbations (e.g. CRISPR-based knockouts or CRISPRi/a), as well as single-cell transcriptomic readouts (Datlinger et al. 2017; Schraivogel et al. 2020; Joung et al. 2023), will be essential for rigorous in vivo validation of predicted regulatory interactions.

**Figure 7:**
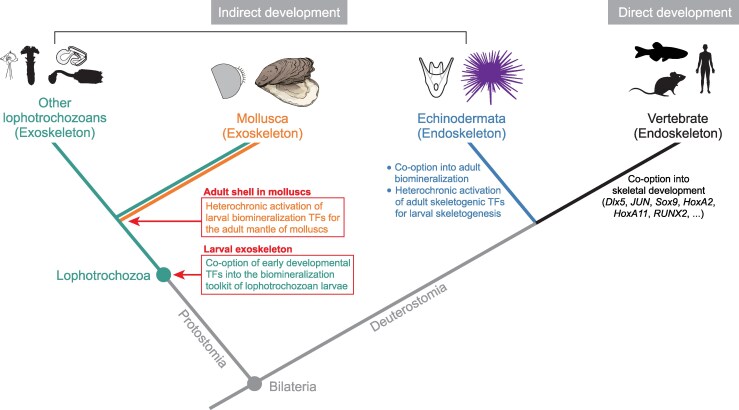
Evolutionary scenario of TF co-option for biomineralized skeletons across bilaterian life cycles. This schematic phylogeny illustrates the convergent and heterochronic co-option of TFs into biomineralization GRNs in major bilaterian clades. In the common ancestor of lophotrochozoans, early developmental TFs were co-opted into larval biomineralization GRNs, establishing a conserved exoskeleton toolkit. In molluscs, these TFs were heterochronically activated in the adult mantle to regulate shell formation. By contrast, adult skeletogenic TFs were repurposed during early development to form the larval endoskeleton in echinoderms, while contributing to adult biomineralization (Erkenbrack and Thompson 2019). Vertebrates, which typically undergo direct development, exhibit recurrent co-option of developmental TFs (e.g. *Dlx5*, *JUN*, *Sox9*, *HoxA2*, *HoxA11*, *RUNX2*) into skeletal development (Salhotra et al. 2020; He et al. 2021; To et al. 2024). These parallel and lineage-specific events highlight the independent evolutionary origins of biomineralized skeletons across Bilateria through the repeated redeployment of ancient regulatory modules.

Regulatory genes underlying bilaterian developmental programs are evolutionarily ancient, predating the origin of animals, and constitute a conserved developmental toolkit that likely facilitated the rapid diversification of metazoans during the Cambrian (Erwin et al. 2011). Emerging evidence, including our findings, suggests that a subset of deeply conserved TFs, originally involved in early embryonic patterning, have been co-opted into the biomineralization toolkit of lophotrochozoans (Huan et al. 2020; Sun et al. 2020, 2021; Alvarez-Campos et al. 2024; Barrera Grijalba et al. 2025). When extending comparisons to deuterostomes, we found eight developmental TFs independently co-opted into the early skeletogenic GRNs in both oyster and sea urchin larvae (Fig. 6c and d). Echinoderm endoskeletons have been considered as a result of reutilization of developmental networks deriving from a single evolutionary origin (Murdock 2020). However, larval skeletogenic cells likely evolved from the heterochronic activation of adult skeletogenic programs (Erkenbrack and Thompson 2019). Although further functional studies are required to test these hypotheses, this is strikingly similar to our proposed model for the evolutionary scenario of adult shell in molluscs, where the heterochronic deployment of larval biomineralization TFs activates the adult shell GRN (Fig. 7). Likewise, our approach also has limitations, as TF binding motif associations were inferred through sequence homology-based transfer from the JASPAR database, specifically built from model metazoan species. The lenient matching strategy we used may limit the accuracy of one-to-one TF-target predictions, such as those identified through ChIP-seq or DAP-seq analyses (Agius et al. 2010; Bartlett et al. 2017). In addition, bulk-level epigenomic profiling, particularly in mixed larval samples, may capture average and noisy signals across heterogeneous cell populations and individuals. The co-occurrence of H3K27ac and H3K27me3 at some genomic loci likely reflects cellular heterogeneity rather than bivalency within single cells, as these modifications cannot coexist on the same histone tail (Fig. 4a). Given the potential cellular heterogeneity within larval and adult biomineralizing tissues, comparative single-cell multi-omic data from molluscs and other biomineralizing lophotrochozoans in future work will be critical for resolving cell-type-specific contributions to the GRNs and transcriptomic similarities we observed. Despite these limitations, our integrative analyses provide valuable insights into the evolutionary strategies of biomineralization GRNs across bilaterians.

Notably, many putative biomineralization TFs in lophotrochozoans and echinoderms belong to gene families also essential for vertebrate skeletogenesis, such as *Dlx5*, *JUN*, *Sox9*, *HoxA2*, *HoxA11*, and *RUNX2* (Salhotra et al. 2020; He et al. 2021; To et al. 2024). Although these TFs are not one-to-one orthologs, their recurrent recruitment into biomineralization programs across diverse bilaterian lineages implies the inherent regulatory potential of these TF families for controlling skeletal development (Fig. 7). This widespread convergence likely reflects a deep evolutionary constraint on upstream regulatory modules, wherein components of ancient developmental toolkits are repeatedly co-opted for similar functional demands during the independent evolution of biomineralized skeletons in bilaterians (Murdock 2020). Beyond mineral deposition, such regulatory conservation may extend to broad physiological roles of skeletal systems. A recent study has revealed that molluscan shells harbor a bone marrow-like hematopoietic stem cell niche, where stem cells contribute to both shell regeneration and systemic blood cell supply, under the regulation of deeply conserved TFs also essential for vertebrate skeletogenesis (Lian et al. 2025). Thus, the molluscan shell highlights how conserved developmental programs can be redeployed through regulatory co-option to generate lineage-specific innovations with ancient physiological roles, such as hematopoiesis, thereby transforming a simple biomineralized secretion into a complex and life-sustaining organ.

In summary, our study provides a novel (epi)genomic and evolutionary framework to understand molluscan shell formation. We highlight that dynamic chromatin landscapes, coupled with conserved regulatory modules co-opted from developmental programs, shape the diversity of biomineralized exoskeletons across life stages. Regardless of the scenario, our data generates novel hypotheses and uncovers a hierarchical decoupling of evolutionary conservation and innovation of biomineralized skeletons, which may represent a general pathway contributing to biological complexity in lophotrochozoans and other bilaterian clades.

## Materials and methods

### Animal culture, sample collection, and shell morphology

*C. nippona* adults were cultured in an oyster farm located in Rushan, China. Embryos and larvae were obtained through artificial fertilization and reared at 26 to 28 ℃. At each key shell-forming stage, including the gastrula (8 hours post fertilization, hpf), trochophore (14 hpf), D-shaped larvae (22 hpf), and later D-shaped larvae (3 days post fertilization, dpf), a single large batch of embryos/larvae was collected and immediately subdivided into multiple cryogenic tubes (each containing >10,000 embryos/larvae), flash-frozen in liquid nitrogen, and stored at −80 °C. These independently stored aliquots were subsequently used as biological replicates for downstream experiments. As embryonic development is not perfectly synchronous, the 8 hpf samples likely include embryos transitioning from morula to early gastrula stages. For scanning electron microscopy (SEM), larvae were fixed in 2.5% glutaraldehyde at 4 °C overnight and processed as described previously (Min et al. 2023).

Whole soft bodies of juvenile oysters (50-day-old, n = 5 per replicate) were pooled, immediately flash-frozen in liquid nitrogen, and stored at −80 °C. To stimulate shell formation, two-year-old adult oysters were shell-damaged by drilling and cultured as previously described (Bai et al. 2023). Once shell repair was completed, mantle tissues surrounding the repaired sites were collected, flash-frozen in liquid nitrogen, and preserved at −80 °C. Repaired shells were carefully separated from the original shells, processed, and imaged under SEM as previously described (Bai et al. 2023).

### Iso-seq and bulk RNA-seq

Total RNA was extracted from samples of all six stages/tissues described above, using TRIzol reagent (Invitrogen, USA). RNA quality assessment was performed using a NanoDrop spectrophotometer and Agilent 5400 Fragment Analyzer system. To obtain a reliable transcript annotation, an Iso-seq library was constructed by pooling equal amounts of RNA from all samples and sequenced on a PacBio Sequel II SMRT cell, generating a total of 64.2 Gb of long reads. In addition, short-read mRNA sequencing was performed on three biological replicates per stage/tissue (Table S1), using the Illumina NovaSeq 6000 platform (PE150), generating ∼ 6 Gb of data per replicate.

### Improved genome annotation of *C. nippona*

Gene prediction was performed using Iso-seq and bulk RNA-seq data generated in this and previous studies (Gong et al. 2021; Bai et al. 2023), following an established pipeline with modifications (Bai et al. 2023). Briefly, short RNA-seq reads were aligned to the *C. nippona* genome using HISAT2 (v.2.2.1) (Kim et al. 2019) and assembled with Trinity (v.2.15.1) (Haas et al. 2013), then integrated with evidence of protein homology and de novo predictions from Augustus (v.3.4.0) (Stanke et al. 2006) with the MAKER pipeline (v.3.01.03) (Cantarel et al. 2008). Iso-seq subreads were processed and aligned to the genome assembly with SMRT Link software (v12.0) (https://www.pacb.com/support/software-downloads/). The TAMA package (Kuo et al. 2020) was used to collapse redundant transcripts and merge full-length transcriptomes. Final gene models were obtained by combining MAKER and Iso-seq annotations using AGAT (v.1.3.0) (https://github.com/NBISweden/AGAT). Completeness was assessed with BUSCO (v.5.4.7) (Manni et al. 2021), and functional annotation was performed using DIAMOND (v2.1.10.164) (Buchfink et al. 2021) against NCBI non-redundant (NR), Uniprot/SwissProt, eggNOG (v.5.0) (Huerta-Cepas et al. 2019), GO categories, and Kyoto Encyclopedia of Genes and Genomes (KEGG) databases, with domain prediction using InterProScan (v.5.64-96.0) (Jones et al. 2014).

### Time-series RNA-seq analysis

Raw RNA-seq reads were processed with fastp (v.0.23.4) (Chen 2023), and gene expression (Transcripts per million, TPM) was quantified using salmon (v.1.10.1) (Patro et al. 2017) with predicted transcript sequences as reference. Trimmed mean of M-values (TMM) normalization was applied by a Trinity utilities script. Differentially expressed genes (DEGs) were determined using edgeR (v.3.40.2) (Robinson et al. 2010) (FDR < 0.05, |log_2_foldchange| > 1).

For clustering analyses, normalized TPM values of biological replicates were averaged to obtain a single gene expression value per gene. This approach was supported by the high concordance among biological replicates within each developmental stage or tissue (Pearson's R = 0.90 to 0.99; Figure S4a), indicating minimal intra-stage variation. *K*-means cluster analysis of expressed genes (normalized TPM > 1 in at least one sample) was performed with *Z*-scaled TPM using the mfuzz package (v.2.62) (Kumar and Futschik 2007). Heatmaps were generated with the ComplexHeatmap package (v.2.18.0) (Gu 2022). GO enrichment for each gene cluster was analyzed using the clusterProfiler package (v.4.10.0) (Xu et al. 2024), against all expressed genes annotated by the eggNOG (v.5.0) database (Huerta-Cepas et al. 2019), with a significance cutoff of Q-value < 0.05.

### Transcription factor (TF) characterization and analysis

We used the AGAT toolkit to generate a *C. nippona* non-redundant proteome with only the longest isoform per gene. Genome-wide identification of TFs was performed by combining homology-based searches against the AnimalTFDB 4.0 database (Shen et al. 2023) using BLASTp (e-value < 1e−10), and de novo domain-based prediction using HMMER (v3.4) and InterPro HMM profiles (Mistry et al. 2021), with the parameter “cut_tc” or a cut-off of 1e−4 for those without trusted cutoffs. The final TF repertoire was defined as the union of results from both methods.

### Shell matrix protein (SMP) extraction

Larval SMPs were extracted from *C. nippona* D-shape larvae (22 hpf) following a modified protocol (Zhao et al. 2018). Five mL of larvae were washed and then centrifuged (1,000 g, 3 min, 4 °C) to remove the supernatant. The remaining shells were immersed in 5% NaOCl for 15 min at 4 °C with gentle shaking, washed with Milli-Q water three times, and then cleaned with a 1-min ultrasound treatment in the Milli-Q water to ensure thorough cleaning (Figure S25). Cleaned larval shells were filtered using a nylon mesh and decalcified in 1 M acetic acid at 4 °C for 12 h. Acid-soluble matrix (ASM) and acid-insoluble matrix (AIM) fractions were separated by centrifugation (4,000 *g*, 30 min, 4 °C), and the AIM fractions were further washed. Both fractions were mixed and concentrated using an Ultracel-10 centrifugal filter unit.

Adult shells (including repaired ones) of three *C. nippona* individuals were treated with 5% NaOCl for 12 h at RT, then washed, fragmented, ultrasonically cleaned for 2 min, and air-dried. After grinding, the shell powder (∼ 40 g) was decalcified in 1 M acetic acid at 4 °C overnight. AIM and ASM were isolated as described above.

### LC-MS/MS analysis and protein identification

AIM and ASM proteins from larvae and adult shells were respectively treated with SDT-lysis buffer (4% SDS, 100 mM DDT, 100 mM Tris-HCl, pH 7.6) in a boiling water bath for 10 min. After cooling to RT, the supernatant was collected by a short centrifugation and mixed with UA buffer (8 M Urea, 150 mM Tris-HCl, pH 8.0). Sample preparation and LC-MS/MS analysis were performed following a previous protocol (Bai et al. 2023). In brief, the mixtures were processed by ultrafiltration, alkylation, enzymatic digestion, and peptide desalting, and then analyzed using a Q-Exactive Plus mass spectrometer coupled with an EASY-nLC 1200 system (Thermo Fisher Scientific).

MS/MS spectra from adult samples and previous datasets (Bai et al. 2023) were searched against the *C. nippona* predicted proteome using MaxQuant (v.2.0.10) (Tyanova et al. 2016) (FDR < 0.01, unique peptides ≥ 2). For larval SMPs, raw spectra were also analyzed as described above similarly. To comprehensively identify the SMPs of the oyster larvae, we ran BLASTp best-reciprocal hits between the *C. nippona* proteome and a larval shell proteome of a closely related oyster species *C. gigas* (Zhao et al. 2018) (e-value < 1e−20, sequence identity > 80%) to retrieve one-to-one orthologues. All proteins identified by both methods were considered as larval SMPs in *C. nippona*. Finally, a total of 101 larval and 289 adult SMPs were identified in *C. nippona* (Table S21). Signal peptides of proteins were predicted with SignalP-6.0 (Teufel et al. 2022).

### Biomineralization gene characterization and analysis

Putative biomineralization TFs in *C. nippona* were identified by BLASTp best-reciprocal hits (e-value < 1e−10) between TFs expressed in *C. gigas* trochophore shell field cells (Piovani et al. 2023) and the *C. nippona* TF repertoire, as well as from known molluscan shell-related TFs based on previous ISH data (Table S4). As this approach relies on sequence similarity and prior expression information from a preliminary scRNA-seq dataset rather than direct functional validation, these genes are conservatively referred to as putative biomineralization TFs. Similarly, putative biomineralization TFs in *S. purpuratus* pluteus larvae were defined as those expressed in skeleton-related cell clusters from published single-cell transcriptome data (Paganos et al. 2021).

In addition to SMPs, we identified other biomineralization effector genes involved in shell formation, including biomineralization enzymes and transmembrane ion transporters (Clark et al. 2020; Sleight 2023; Li et al. 2024), by searching the *C. nippona* proteome against InterPro HMM profiles (Mistry et al. 2021) using HMMER (v3.4) with the “cut_tc” parameter (Table S22). RNA-seq data of adductor muscle, gills, digestive gland, and hemolymph from our previous study (Bai et al. 2023) were also processed following the same pipeline described above and were normalized together with newly generated RNA-seq data. Gene co-expression analysis was performed using the WGCNA package (Langfelder and Horvath 2008) across multiple developmental stages and adult tissues, and identified 11 gene modules (MEs) significantly correlated with shell-forming stages and mantle tissue (Figure S26). Effectors within these modules were considered involved in *C. nippona* shell formation.

Biomineralization genes in other molluscs (*C. gigas*, *Pinctada fucata*, *Patinopecten yessoensis*, *Haliotis rufescens*, *Pomacea canaliculata*) were identified using similar approaches. SMP data for *P. fucata* larvae, *P. canaliculata* adult, and *C. gigas* larvae and adult were obtained from previous studies (Zhao et al. 2018; Liu et al. 2023). MS/MS datasets for *P. fucata* adult shells (PXD006786 and PXD010130) were downloaded from the PRoteomics IDEntifications Database (PRIDE), and protein identification was performed using MaxQuant (v.2.0.10) (Tyanova et al. 2016). For species lacking SMP data, only biomineralization enzymes and transmembrane ion transporters were considered. WGCNA analysis was performed to further identify candidate biomineralization effector genes in these molluscs, which were highly expressed in larval or adult shell- related modules (Figure S27).

### ATAC-seq and CUT&Tag experiments

We performed two replicates of ATAC-seq on five developmental stages and adult mantle tissue used for RNA-seq of *C. nippona*, using ∼ 50,000 cells per sample. After tissue dissociation and filtration, cell numbers were estimated by mixing 10 µL of cell suspension with 10 µL of trypan blue, loading 10 µL of the mixture onto a counting chamber, and measuring cell concentration and viability using an automated cell counter. Cell suspensions were then adjusted to the desired concentration before proceeding with the Omni-ATAC protocol (Corces et al. 2017). After nuclei isolation and tagmentation at 37 °C for 30 min, libraries were purified, PCR-amplified (12-cycle) (TruePrep® DNA Library Prep Kit V2 for Illumina; primers from TruePrep® Index Kit V2 for Illumina), and then size-selected (0.55× and 1.5× ratios) using VAHTSTM DNA Clean Beads as indicated by the supplier. Final libraries were quantified (> 10 ng/μL) using a Qubit 4 fluorometer and quality checked for fragment distribution on a Qsep-400 system (Bioptic), ensuring a clear ladder pattern with a major peak around 200 bp, no smearing or adapter contamination. Sequencing was performed on an Illumina NovaSeq 6000 platform in PE150 mode.

CUT&Tag assays were conducted with two replicates of D-shaped larva and adult mantle samples (∼ 100,000 nuclei per sample) used for RNA-seq and ATAC-seq of *C. nippona*, following a previous protocol with minor modifications (Kaya-Okur et al. 2019). Nuclei were bound to activated concanavalin A-coated magnetic beads, resuspended in Dig-Wash Buffer (0.05% digitonin and 2 mM EDTA). Primary antibodies, including H3K4me3 (Active motif, #61379), H3K27ac (Active motif, #39133) and H3K27me3 (Abcam, #AB6002), were added at a 1:50 dilution and incubated at room temperature for 2 h, followed by a 1:50 dilution of secondary antibody (IgG, Proteintech, #B900210) for 1 h. After washing, nuclei were treated with pG-Tn5 adapter complex (1:200 in Dig-300 Buffer) for 1 h, followed by tagmentation in 10 mM MgCl₂ at 37 ℃ for 1 h. The reaction was terminated with the addition of 0.5 M EDTA, 10% SDS, and Proteinase K, and samples were incubated at 55 °C for 1 h. DNA was then purified using phenol-chloroform and ethanol precipitation. Libraries were PCR-amplified (16 cycles), purified with AMPure XP beads (Beckman Coulter), and sequenced on an Illumina NovaSeq 6000 system (PE150).

### ATAC-seq and CUT&Tag data analysis

Raw reads were quality filtered with fastp (v.0.23.4) (Chen 2023) and mapped to the *C. nippona* genome using Bowtie2 (v.2.5.2) (Langmead and Salzberg 2012). PCR duplicates, unmapped reads, multi-mapped reads, low-quality reads (MAPQ < 20), and reads mapped to the mitochondrial genome were removed using the alignmentSieve tool from deepTools (v.3.5.4) (Ramírez et al. 2016). For ATAC-seq data, the mapped reads were shifted to +4/−5 bp depending on the strand of the reads to reflect Tn5 cleavage sites. Peak calling was performed using MACS3 (v.3.0.0b3) (Zhang et al. 2008) (ATAC-seq: –nomodel –shift –75 –extsize 150 –qvalue 0.05; H3K4me3 and H3K27ac: –qvalue 0.05; H3K27me3: –broad –broad-cutoff 0.05). Reproducible peaks across biological replicates were identified using the irreproducible discovery rate method (IDR < 0.05) (v.2.0.4). For data visualization, merged BAM files of two biological replicates were converted to RPKM (Reads Per Kilobase per Million mapped reads) normalized bigwig files with 10 bp bin size using deepTools (v.3.5.4) (Ramírez et al. 2016). Peak tracks and gene structures were visualized with pyGenomeTracks (v.3.9) (Lopez-Delisle et al. 2021). Consensus peaks were generated with DiffBind (v.3.12.0) (Ross-Innes et al. 2012) and annotated to genomic regions using UROPA (Kondili et al. 2017). The whole genome was divided into four regions: promoter (from −2,000 bp to +500 bp of TSS), genic (from +500 bp of TSS to TES), distal (other region within 100 Kb of the nearest gene), and no annotation (>100 Kb away from the nearest gene). Reads count were quantified using featureCounts (Liao et al. 2014) and TMM-normalized by the Trinity utilities script. Differential peaks were identified using edgeR (v.3.40.2) (Robinson et al. 2010) (FDR < 0.05, |log_2_foldchange| > 0).

### Chromatin state analysis

We used ChromHMM (v.1.26) (Ernst and Kellis 2017) with the default parameter to train the chromatin state prediction model by integrating CUT&Tag (H3K4me3, H3K27ac, and H3K27me3) and ATAC-seq data merged from two biological replicates of D-shape larvae and adult mantles. Models ranging from 4 to 16 states were built, and an eight-state model on strong correlations among states, ensuring a balance between depth and clarity in the results (Figure S7a). Binarization was performed at a resolution of 200 bp. The annotation of each state was achieved by the “OverlapEnrichment” and “NeighborhoodEnrichment” functions provided by ChromHMM.

### GRNs construction

GRNs for larval and adult shell formation were constructed by integrating established methods (Lowe et al. 2022; Marletaz et al. 2023; Pérez-Posada et al. 2024) with slight modifications (Figure S10a). Genes with TPM > 1 and TF binding motifs in active promoter or genic regions were used to build cis-regulation networks. Motif information was obtained from the JASPAR non-redundant CORE metazoan database, and *C. nippona* TF motifs were inferred by reciprocal BLASTp best-hits (e-value < 1e−10, identity > 30%) against JASPAR TFs (Table S11). For ANANSE (Xu et al. 2021), ATAC-seq IDR peaks with at least 50% overlap with poised or active regions were extracted and re-centered their coordinates on the summits of chromatin accessibility peaks. ANANSE binding was run with ATAC-seq, CUT&Tag (H3K27ac) BAM files, and the summit-centered peaks to generate genome-wide TF binding profiles for D-shape larvae and adult mantles, respectively. The ANANSE network was then used to construct GRNs, respectively for larvae and adults, based on the predicted TF binding profiles and RNA-seq data. The top 25% of regulatory edges (probability score > 75th quantile) were retained as the final ANANSE networks. For TF footprinting analysis, ATAC-seq BAM files of two biological replicates were merged using samtools (v.1.13) (Danecek et al. 2021) and analyzed with TOBIAS (v.0.16.1) (Bentsen et al. 2020) to identify bound TF binding sites (footprint score above BINDetect threshold). Footprint-based networks were constructed for larval (trochophore and D-shaped stages) and adult (mantle tissue) phases. Final GRNs were generated by combining ANANSE and footprint networks, defining biomineralization genes and TFs as targets. Networks were visualized with Cytoscape (v.3.10.1) (https://cytoscape.org/), and TF centrality (outdegree index) was calculated using the igraph R package. The same pipeline was applied to establish GRNs for skeleton formation in *S. purpuratus* larvae, using ATAC-seq peaks and gene expression data, with positive marker genes from skeleton-related clusters in the published single-cell transcriptome (Paganos et al. 2021) as targets.

### Phylostratigraphy analysis

Gene age was estimated for both species using the GenERA tool (v.1.4.2) (Barrera-Redondo et al. 2023), which performs genomic phylostratigraphy by searching for homologs in the NCBI NR database (Release 2024_01_01) and predicts gene origins based on NCBI Taxonomy. Biomineralization effector genes were classified as pre-Mollusca if they originated at or before the Cellular organisms-Lophotrochozoa ancestor, while genes originating from between Mollusca and Mollusca-specific families/species were considered Mollusca-family/species origin genes (Figure S9a and Table S23). Paralog identification and dating in *C. nippona* and five other molluscs followed a previously described protocol (Singh et al. 2024). Paralogs and their duplication timing across 40 metazoan species (Table S24) were inferred using OrthoFinder (v2.5.2) (Emms and Kelly 2019). The phylostratum of each duplication node was classified as described above (Figure S9a).

### Classification of regulatory divergence among functionally diverged paralogs

To define the regulatory divergence of biomineralization gene paralogs, we compared their expression levels and enrichment of active histone modifications (H3K4me3 and H3K27ac) between D-shaped larvae and adult mantle tissue. A gene was considered regulatory, specialized for larval shell formation, if it showed significantly higher expression and stronger enrichment of associated histone marks in larvae. In contrast, a gene was considered regulatory, specialized for adult shell formation if it exhibited higher expression and histone signals in the adult mantle. Gene pairs with no significant differences in both expression and regulatory marks between stages were defined as shared regulators for both larval and adult shell formation. Within this framework, regulatory divergence among paralogous pairs could be classified into three patterns: no divergence, where both genes show similar regulatory profiles across stages, with no significant differences in H3K4me3 or H3K27ac levels between larval and adult stages; one-sided divergence, where only one paralog exhibits stage-specific enrichment of histone modifications, suggesting specialization driven by divergence in a single gene; and both-sided divergence, where the two genes display distinct, reciprocal regulatory profiles, with one specialized in larvae and the other in adults, reflecting coordinated acquisition of stage-specific cis-regulatory landscapes. This classification allowed us to systematically link expression specialization with regulatory evolution and support the role of epigenetic remodeling in the divergence of biomineralization gene function across oyster life stages.

### Comparative analyses using TFs

We identified putative one-to-one orthologs of 29 putative biomineralization TFs between *C. nippona* and ten other lophotrochozoans, as well as *A. amphitrite*, by performing reciprocal best-hit BLASTp searches with an e-value threshold of 1e−3 (Table S17). The results were consistent with those obtained using OrthoFinder (v2.5.2) (Emms and Kelly 2019). Publicly available RNA-seq datasets of developmental time courses and adult tissues for these species were downloaded from the NCBI SRA database (Table S25) and processed using the same pipeline described above for *C. nippona*. Following established methods from a previous study (Martin-Zamora et al. 2023), we then performed a quantile transformation to TPM values and calculated the JSD comparing the expression profiles of the 29 single-copy orthologous TFs between each species and *C. nippona*. To ensure statistical robustness, 1,000 bootstrap replicates were performed to estimate mean JSD values. Raw mean JSD values were adjusted by the number of orthologs and normalized using the global minimum and maximum of all adjusted values across comparisons. Relative JSD values were similarly normalized within each pairwise comparison. Transcriptomic divergence (JSD values) of putative biomineralization TFs between *S. purpuratus* and two other echinoderms was calculated using the same method (Tables S19 and S25), yielding mean and standard deviation values.

### ISH experiments

Gene-specific primers containing SP6/T7 adaptor sequences were designed using Primer Premier (v.6.0) (Premier Biosoft International, Palo Alto, CA) to amplify cDNA fragments for each gene (Table S26). Digoxigenin-labelled RNA probes (antisense and sense) were synthesized using purified PCR products and DIG RNA labeling Kit (SP6/T7) (Roche, Switzerland). The synthesized probes were purified using the MEGAclear™ Transcription Clean-Up Kit (Thermo Fisher Scientific, USA) and stored at −80 °C until use.

ISH in oyster larvae and adult mantles was performed as previously described (Bai et al. 2023; Min et al. 2023). Larvae and adult mantles were fixed in 4% paraformaldehyde (PFA) overnight at 4 °C, dehydrated with 100% methanol, and stored at −30 °C. For ISH of larvae, samples were rehydrated, digested with proteinase K (with D-shape larvae pre-treated with 0.5 M EDTA), post-fixed in 4% PFA, pre-hybridized, and hybridized overnight at 65 °C with 500 ng/mL RNA probe at 65 °C. After washing, samples were blocked and then incubated with anti-DIG AP antibody (1:5000) (Roche, Switzerland) overnight at 4 °C. Finally, samples were washed and incubated with NBT/BCIP substrate (Roche, Switzerland) for signal detection. For ISH of adult mantles, fixed mantles were cleared in xylene, embedded in paraffin, and sectioned at 5 μm. After deparaffinization, rehydration, proteinase K digestion, prehybridization, hybridization (with RNA probes at 1 ng/mL), followed by antibody incubation (1:3000) and NBT/BCIP staining at 4 °C. Negative controls were done for all genes using sense probes, as well as by including all hybridization ingredients except probes. Images were acquired using an Olympus BX53 microscope.

### Luciferase reporter assay

Dual-luciferase reporter assays were performed to evaluate the transcriptional activities of two predicted promoters (peak18565 for *Gigasin2*, and peak151754 for *Unc022929*) following our previously published protocol (Min et al. 2024). Genomic DNA from the mantle tissue of three *C. nippona* adults was extracted using the phenol-chloroform method. The promoter sequences and coding sequence of *Hox4* were amplified and cloned into pGL3-basic and pcDNA3.1(+) plasmids, respectively (Table S26). HEK293T cells were seeded in 24-well plates (Corning, USA) and cultured in DMEM (Hyclone, USA) with 1% 1 × penicillin-streptomycin antibiotics and 10% fetal bovine serum (FBS) (Hyclone, USA) at 37 °C with 5% CO_2_. Once cells reached 80% confluence, they were transfected with serum- and antibiotic-free medium using Lipofectamine 3000 (Invitrogen, USA) at a 1:1 ratio of promoter to TF plasmids. DNA-lipofectamine complexes were added dropwise to the medium. After 48 h, luciferase activity was measured using the Dual-Luciferase Reporter Assay System (Promega, USA) according to the manufacturer's instructions. Firefly and Renilla luciferase activities were quantified using the Synergy™ H1 Multimode Reader (BioTek, USA). Relative luciferase activity was assessed by calculating the ratio of firefly luciferase activity to Renilla luciferase activity. Each targeted fragment was tested in triplicate.

### Knockdown of *Hox4* using RNAi

A shell repair assay was performed on two-year-old *C. nippona* adults (n = 15 per group). A hole (∼2 mm in diameter) was drilled into the left valve of each oyster. Oysters were divided into three groups: *Hox4* siRNA (*Hox4*-RNAi), negative control siRNA (NC), and DEPC water control. *Hox4*-RNAi and NC were synthesized by GenePharma (Shanghai, China) (Table S24). The selected siRNA was optimized in a pilot experiment (Figure S28 and Table S26). Based on the results, we selected the most effective siRNA “*Hox4*-437”. The siRNAs were diluted to 1 µg/µL in DEPC-treated water containing phenol red (1:20 dilution) for visual tracking during injection. A small gap was created near the adductor muscle on the right shell using forceps, allowing access for injection. In the experimental group, each oyster was injected with 20 µL of *Hox4*-RNAi solution into the pericardial cavity using a microsyringe. The control groups were treated with an equal volume of NC solution and DEPC water, respectively. Injections were performed every 48 h for a total of three times. During the experiment, oysters were cultured at 18 to 20 °C and fed daily with *Chlorella* sp. No oyster died during the experiments. Drilled holes were fully covered by the newly repaired shell in the majority of control oysters by day 6 after drilling, and mantle tissues surrounding the shell holes were collected for mRNA and protein quantification. Newly repaired shells were gently rinsed with filtered seawater to remove surface debris without applying mechanical force. After rinsing, the shells were subjected to a brief (1 min) gentle ultrasonic cleaning step and then observed to confirm the completeness of repaired shells. Repaired shells were subsequently photographed and analyzed using ImageJ (https://imagej.net/ij/) to assess the areas of newly repaired shells and calculate shell repair rates (Figure S17). For SEM analyses, shell pieces centered on the repaired region were excised and then fractured to expose the cross-sections through the repaired shell layers and the inner surface of the repaired shell. Based on SEM observation, the semi-translucent repaired region corresponds to the newly repaired prismatic and foliated layers, whereas the opaque, milky-white region represents the chalky layer; all of these structures are fully mineralized.

### Real-time quantitative polymerase chain reaction (RT-qPCR) analysis

Total RNA was extracted from the mantle tissues of seven randomly selected individuals from each group in the RNAi experiment using TRIzol reagent (Invitrogen, USA), following the manufacturer's protocol. Total RNA (1 µg) was reverse transcribed into cDNA using the Evo M-MLV RT Mix Kit (Accurate Biotechnology, China) according to the manufacturer's instructions. Primers for the *Hox4* gene and internal control genes *EF1α* and *GAPDH* were designed with Primer Premier (v.6.0) (Table S26). All primer pairs were validated by melting curve analysis. RT-qPCR was performed using the SYBR Green Premix Pro Taq HS qPCR Kit (Accurate Biotechnology, China) on a LightCycler 480 real-time PCR system (Roche, Switzerland). Relative gene expression levels were calculated using the 2^−ΔΔCT^ method (Livak and Schmittgen 2001).

### Western blot analysis

Mantle tissues from three individuals, also used for RT-qPCR, each from the NC and *Hox4*-RNAi groups, were homogenized in PBS and centrifuged to collect the supernatant. Protein concentrations were quantified using a BCA Protein Assay Kit (Beyotime, China). Samples were diluted to equal concentrations with SDS-PAGE loading buffer (Solarbio, China), denatured at 95 °C for 10 min, and 40 µg of protein per sample was separated on a 12% SDS-PAGE gel. The separated proteins were transferred onto 0.4 µm polyvinylidene fluoride (PVDF) membranes (Beyotime, China). Membranes were blocked at room temperature for 2 h with 5% non-fat milk in TBST buffer and then incubated overnight at 4 °C with primary antibodies, including Hox4 (Thermo Fisher Scientific, USA, #H00003221-M02) and β-actin (Beyotime, China, #AF0003). After three washes with TBST, membranes were incubated with HRP-conjugated goat anti-rabbit secondary antibody (1:1000, Beyotime, China) at RT for 2 h. Finally, the blots were measured with SuperPico ECL chemiluminescence kit (Vazyme, China) and visualized using the GE ImageQuant LAS4000mini system (GE, USA). The experiments were repeated twice in independent experiments using the same protocol, and yielded consistent results (Figure S29 and Table S27).

### RNA-seq after RNAi experiment

Total RNA from the mantle tissues of three individuals for Western blot analysis was used for short-read mRNA sequencing, with three biological replicates. Sequencing was performed using the Illumina NovaSeq X Plus platform (PE150), generating ∼ 6 Gb of data per replicate. The analysis workflow of the RNA-seq data was conducted as described in the previous section.

## Supplementary Material

msag019_Supplementary_Data

## Data Availability

The raw sequence data have been deposited on NCBI under the BioProject accession numbers PRJNA1267763 and PRJNA1268133. The shell proteomic data are available through ProteomeXchange under accession PXD064366. The improved genome annotation of *C. nippona* generated in this study has been deposited in MolluscDB (http://mgbase.qnlm.ac) and is also accessible on Figshare (https://doi.org/10.6084/m9.figshare.29204657.v1). The scripts and codes for this study can be found on GitHub: https://github.com/StevenBai97/OysterChromatinDynamics.
