# Supplementary material for: Evolutionary innovation within conserved gene regulatory networks underlying biomineralized skeletons in Bilateria
Source: Mol Biol Evol. 2026 Jan 20;43(2):msag019. doi: 10.1093/molbev/msag019 (PMC12862220; doi:10.1093/molbev/msag019)
Supplement: msag019_Supplementary_Data [file msag019_supplementary_data.zip › Supplementary figures.pdf]

SUPPLEMENTARY FIGURES

Evolutionary innovation within conserved gene regulatory networks underlying biomineralized skeletons in Bilateria

Yitian Bai[1 †], Yue Min[1 †], Shikai Liu[1 †], Yiming Hu[1], Shulei Jin[1], Hong Yu[1], Lingfeng Kong[1], Daniel J. Macqueen[3], Shaojun Du[4] and Qi Li[1,2*]

[1]Key Laboratory of Mariculture, Ministry of Education, Ocean University of China, Qingdao 266003, China

[2]Laboratory for Marine Fisheries Science and Food Production Processes, Qingdao Marine Science and Technology Center, Qingdao 266237, China

[3]The Roslin Institute and Royal (Dick) School of Veterinary Studies, The University of Edinburgh, Midlothian, UK

[4]Institute of Marine and Environmental Technology, Department of Biochemistry and Molecular Biology, University of Maryland School of Medicine, Baltimore, MD, USA

[†] These authors contributed equally.

*Correspondence: qili66@ouc.edu.cn

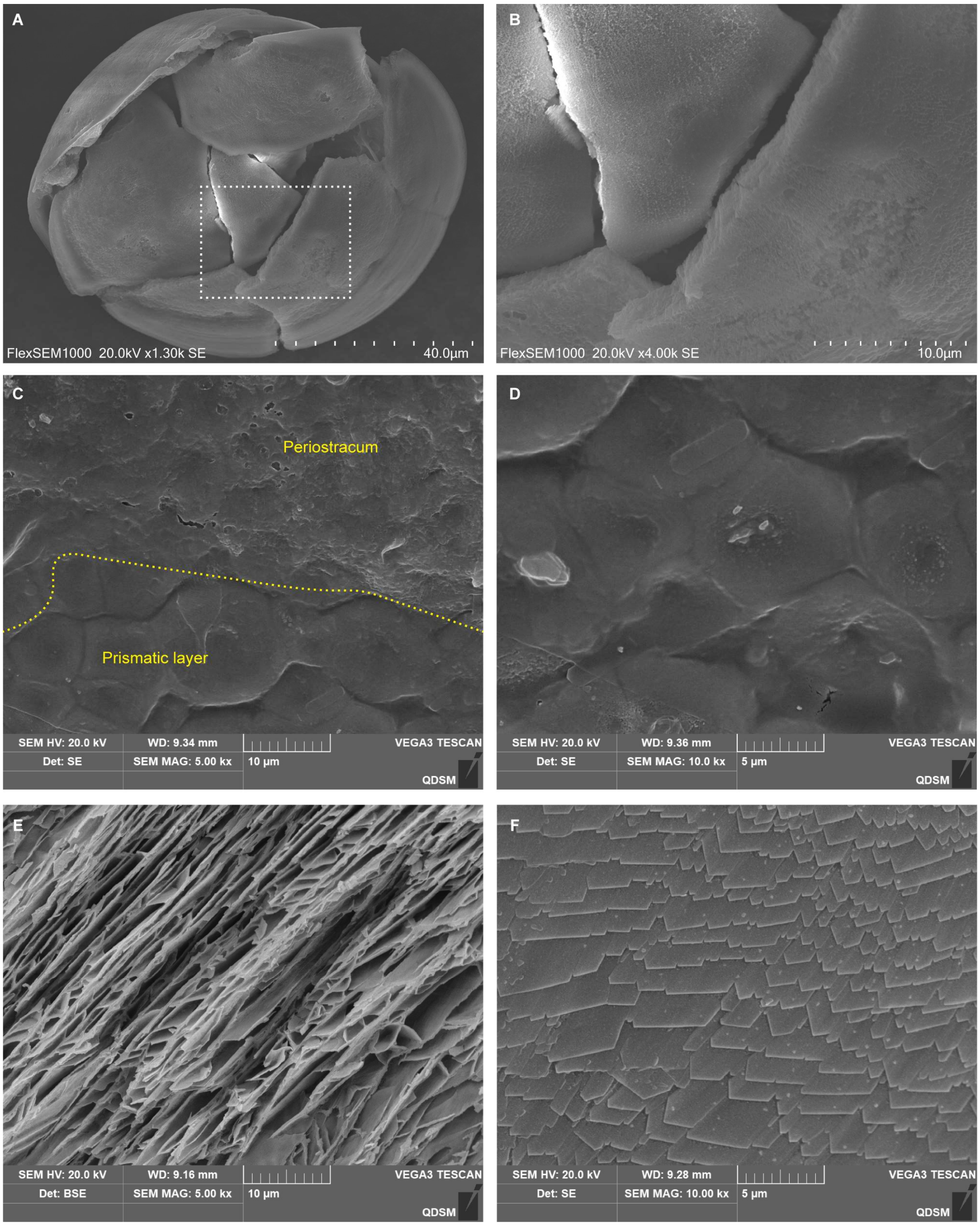

Fig. S1. Morphology and structure of larval and adult shells in *C. nippona* showed by scanning electron microscopy (SEM). **(a)** Aragonitic shell of the D-stage larva at 72 hours post fertilization. **(b)** Magnified view of the region outlined by the white dashed box in **(c)**. **(c)** Outer surface of the adult calcitic shell. The area above the yellow dashed line corresponds to the periostracum, while the area below corresponds to the prismatic

layer. **(d)** Outer surface of the prismatic layer in the adult calcitic shell. **(e)** Inner surface of the chalky layer in the adult calcitic shell. **(f)** Inner surface of the foliated layer in the adult calcitic shell.

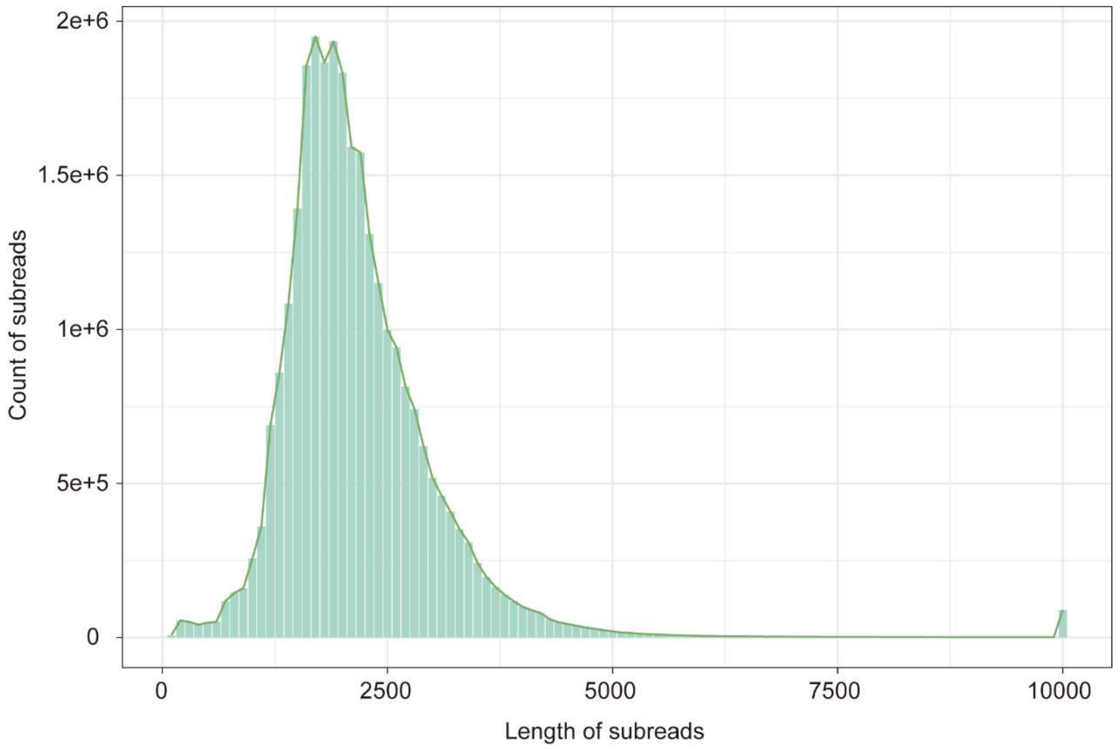

Fig. S2. Statistics of length distributions of the raw subreads from Iso-seq.

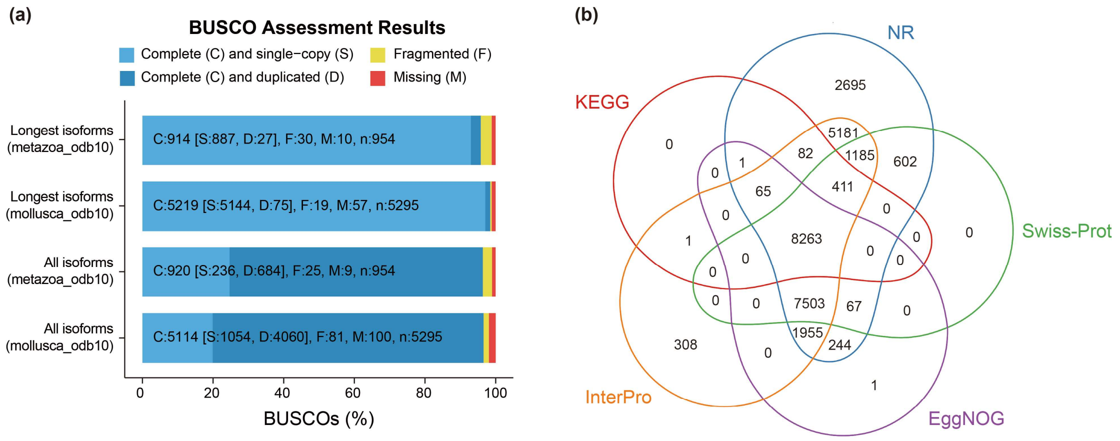

Fig. S3. Quantify assessment of improved gene predictions in *C. nippona*. **(a)** BUSCO analysis of the predicted protein sequences using both the longest isoforms and all isoforms, based on the metazoa_odb10 and mollusca_odb10 databases. **(b)** Venn diagram showing the overlap of functionally annotated protein-coding genes across five databases: NR, Swiss-Prot, KEGG, InterPro, and eggNOG. The numbers represent the counts of genes uniquely or commonly annotated in each database.

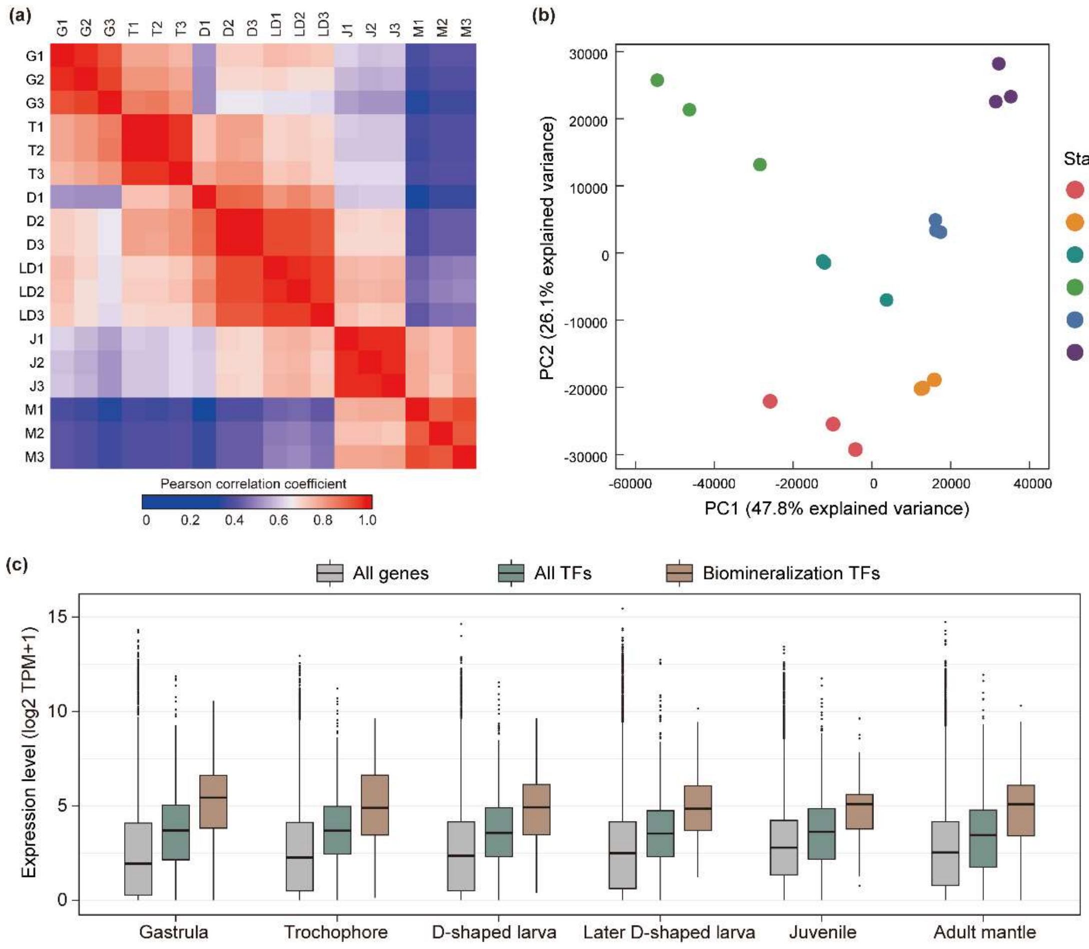

Fig. S4. Gene expression dynamics across six developmental stages in *C. nippona*. **(a)** Correlation matrix based on gene expression profiles, indicating strong within-stage consistency and distinct stage-specific transcriptomic signatures. **(b)** Principal component analysis (PCA) of gene expression, showing stage-wise separation along PC1 (47.8% variance explained) and PC2 (26.1% variance explained). **(c)** Expression levels of all genes, all transcription factors (TFs), and biomineralization TFs across six developmental stages. Biomineralization TFs show consistently higher expression levels than other TFs or all genes, particularly during shell-forming stages. G: gastrula, T: trochophore, D: D-shaped larva, LD: later D-shaped larva, J: juvenile, M: adult mantle.

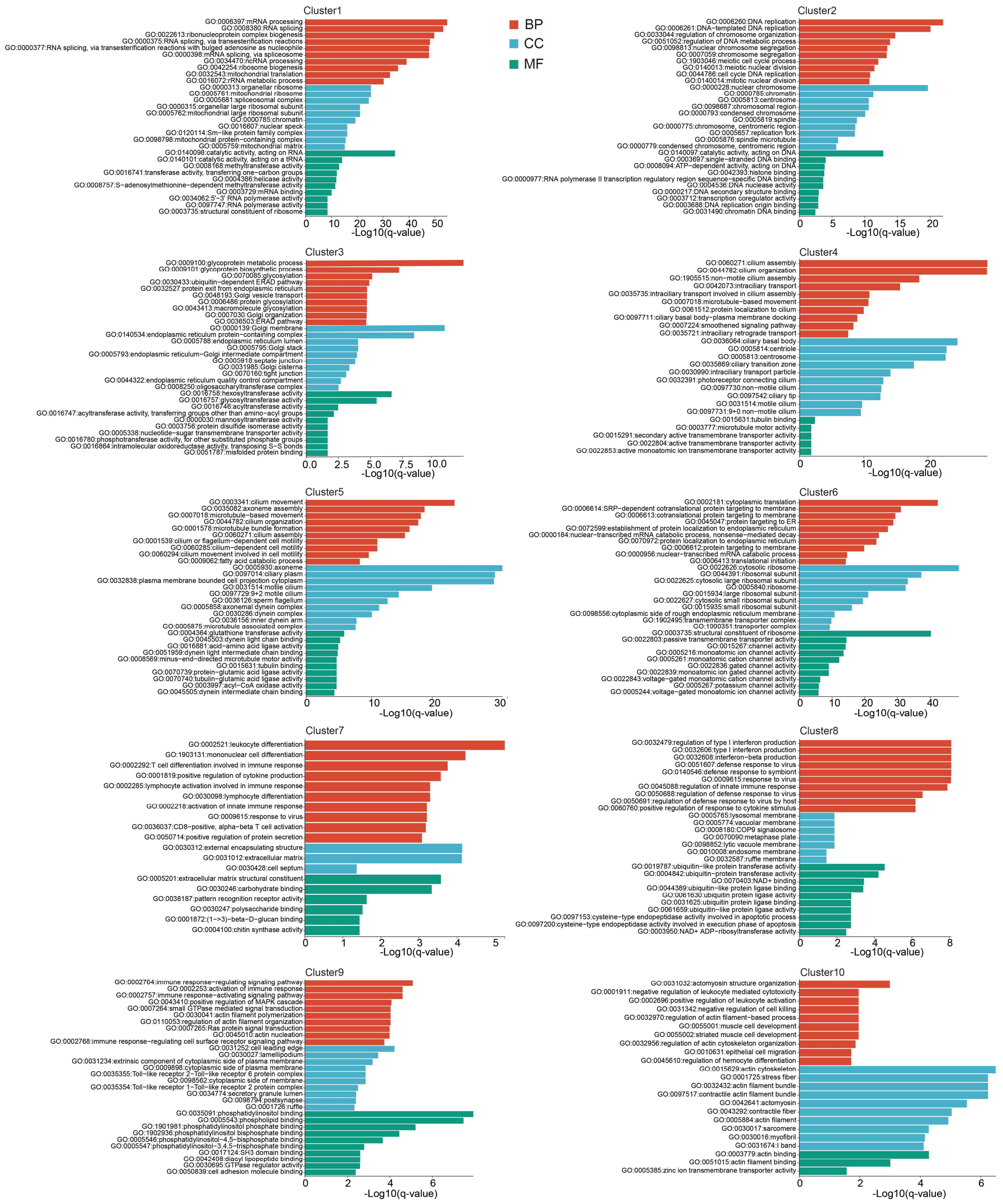

Fig. S5. GO terms enrichment of RNA-seq cluster of *C. nippona*. Bar plots depicting adjusted *P*-values (q-value) of the top 10 GO terms for biological process (BP), cell component (CC), and molecular function (ME).

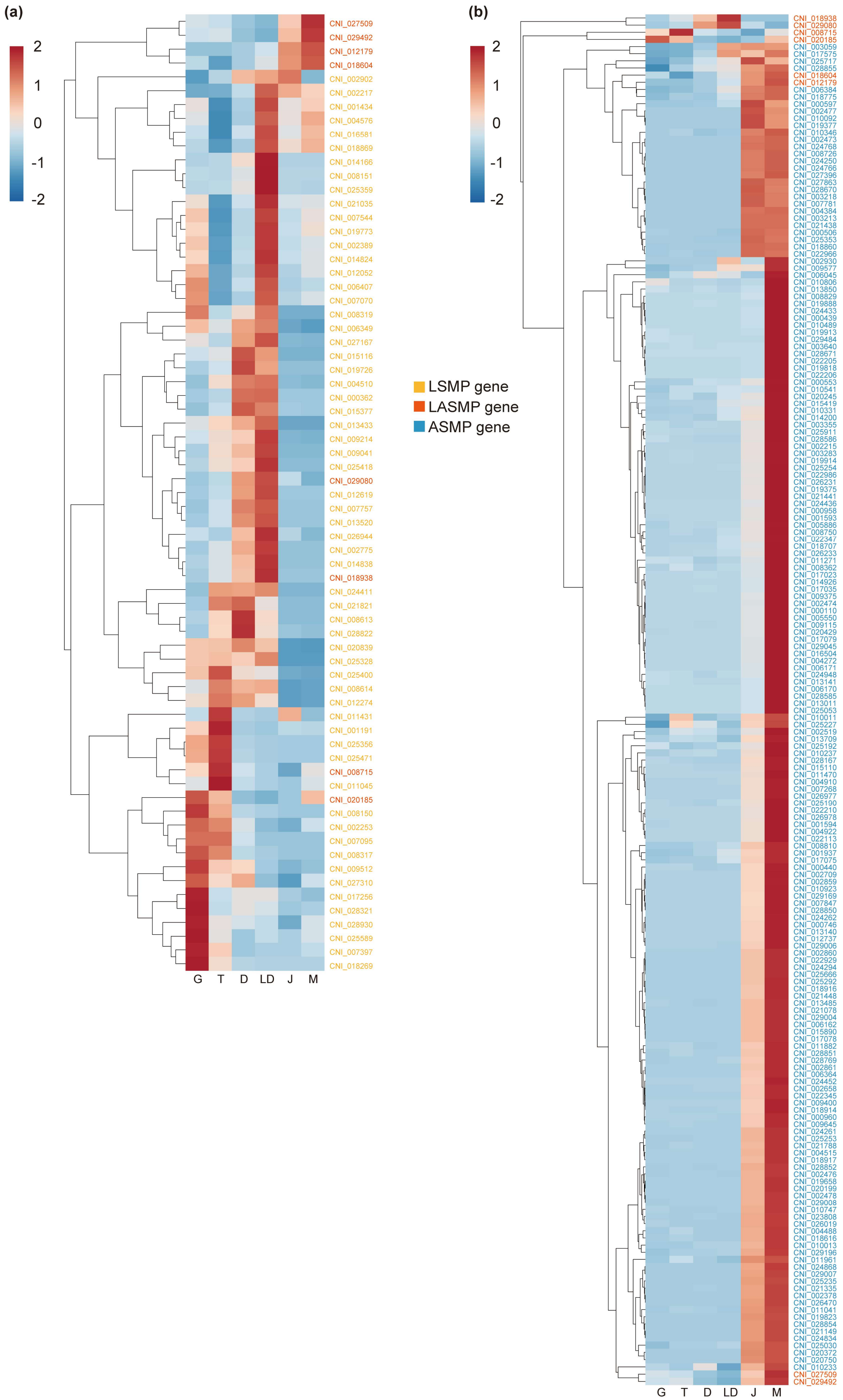
(a)
2
1
0
-1
-2
CNI_027509
CNI_029492
CNI_012179
CNI_018604
CNI_002902
CNI_002217
CNI_001434
CNI_004576
CNI_016581
CNI_018869
CNI_014166
CNI_008151
CNI_025359
CNI_021035
CNI_007544
CNI_019773
CNI_002389
CNI_014824
CNI_012052
CNI_006407
CNI_007070
CNI_008319
CNI_006349
CNI_027167
CNI_015116
CNI_019726
CNI_004510
CNI_000362
CNI_015377
CNI_013433
CNI_009214
CNI_009041
CNI_025418
CNI_029080
CNI_012619
CNI_007757
CNI_013520
CNI_026944
CNI_002775
CNI_014838
CNI_018938
CNI_024411
CNI_021821
CNI_008613
CNI_028822
CNI_020839
CNI_025328
CNI_025400
CNI_008614
CNI_012274
CNI_011431
CNI_001191
CNI_025356
CNI_025471
CNI_008715
CNI_011045
CNI_020185
CNI_008150
CNI_002253
CNI_007095
CNI_008317
CNI_009512
CNI_027310
CNI_017256
CNI_028321
CNI_028930
CNI_025589
CNI_007397
CNI_018269
G T D LD J M
LSMP gene
LASMP gene
ASMP gene
(b)
2
1
0
-1
-2
G T D LD J M

Fig. S6. Gene expression patterns of larval **(a)** and adult **(b)** shell matrix proteins across six developmental stages in *C. nippona*. G: gastrula, T: trochophore, D: D-shaped larva, LD: later D-shaped larva, J: juvenile, M: adult mantle, LSMP: larval shell matrix protein, LASMP: larval and adult shell matrix protein, ASMP: adult shell matrix protein.

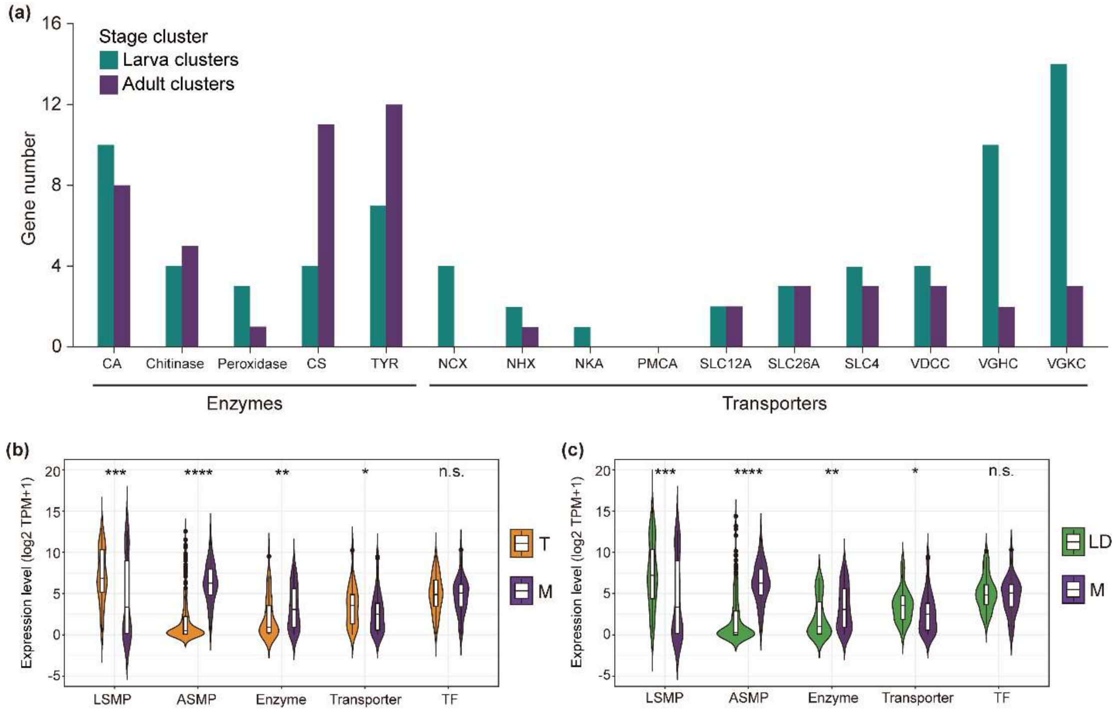

Fig. S7. Expression patterns of biomineralization effector genes in *C. nippona*. **(a)** Number of genes in each biomineralization effector family identified in larval and adult expression clusters. LSMP, larval shell matrix protein; ASMP, adult shell matrix protein; CA, carbonic anhydrase; CS, chitin synthase; TYR, tyrosinase; NCX, Na^+/Ca^{2+}-exchangers; NHX, Na^+/H^+ exchangers; NKA, Na^+/K^+-ATPase; PMCA, Ca^{2+}-ATPase; VGHC, voltage-gated H^+ channel; VGHC, voltage-gated K^+ channel; VDCC, voltage-dependent Ca^{2+} channel; SLC, solute carrier; TF, transcription factor. **(b and c)** Comparison of expression levels for each effector category between the trochophore and adult stages **(b)**, or between the later D-shaped larva and adult stages **(c)**. Boxplots include a median with quartiles and outliers above the top whisker. Statistical significance was assessed using a two-sided Wilcoxon rank-sum test (*$P < 0.05$; **$P < 0.01$; ***$P < 0.001$; ****$P < 0.0001$; n.s., no significance).

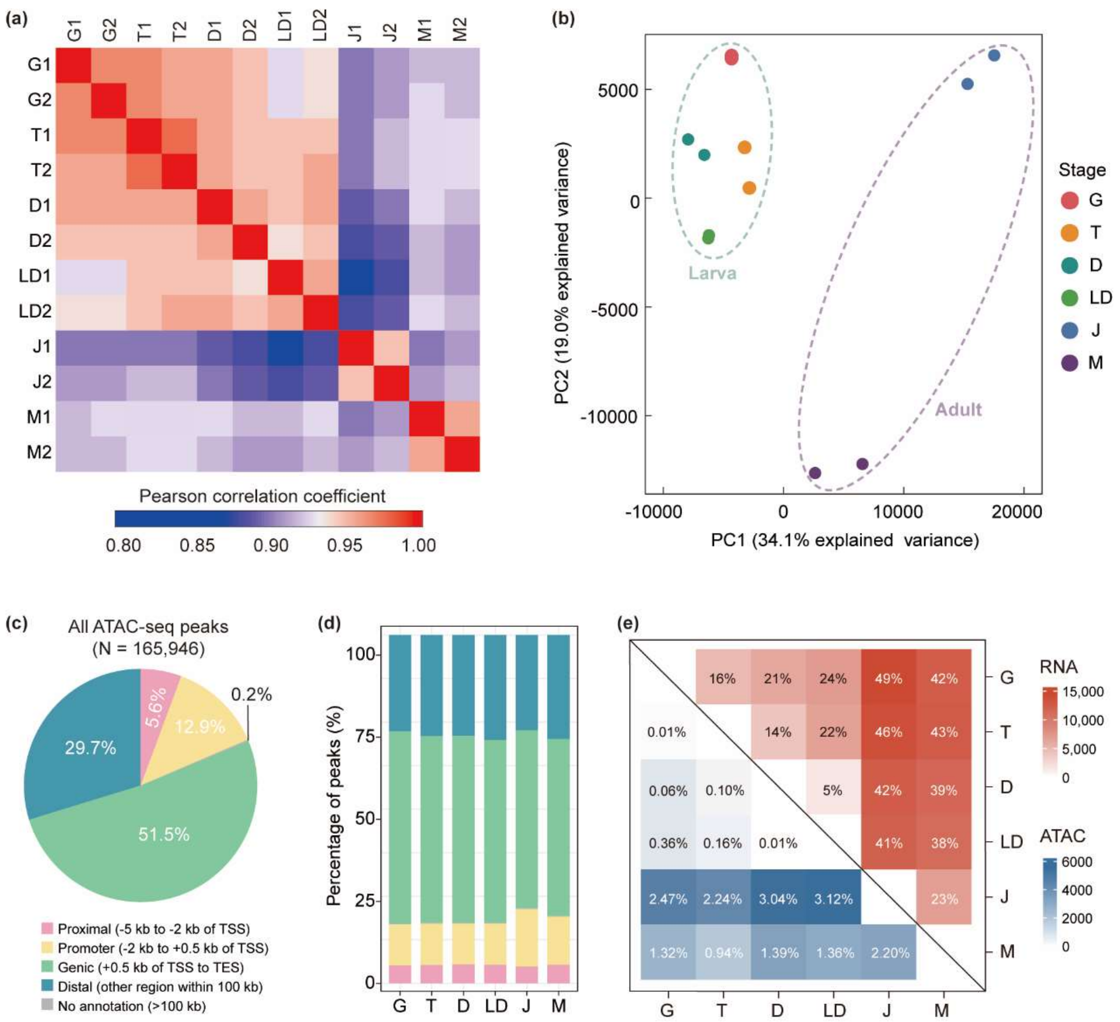

Fig. S8. Chromatin dynamics across six developmental stages or tissues in *C. nippona*. **(a)** Correlation matrix based on peak accessibility of the consensus ATAC-seq peak. **(b)** PCA of ATAC-seq samples, showing stage-wise separation along PC1 (34.1% variance explained) and PC2 (19.0% variance explained). The green and purple dashed circles outline the distribution of larval and adult samples, respectively. **(c)** Genomic feature annotation of the consensus ATAC-seq peaks. **(d)** Stacked bar plots showing the proportion of called peaks per developmental stage classified by genomic feature. **(e)** Number and proportion of differentially expressed genes (DEGs) and differentially accessible peaks.

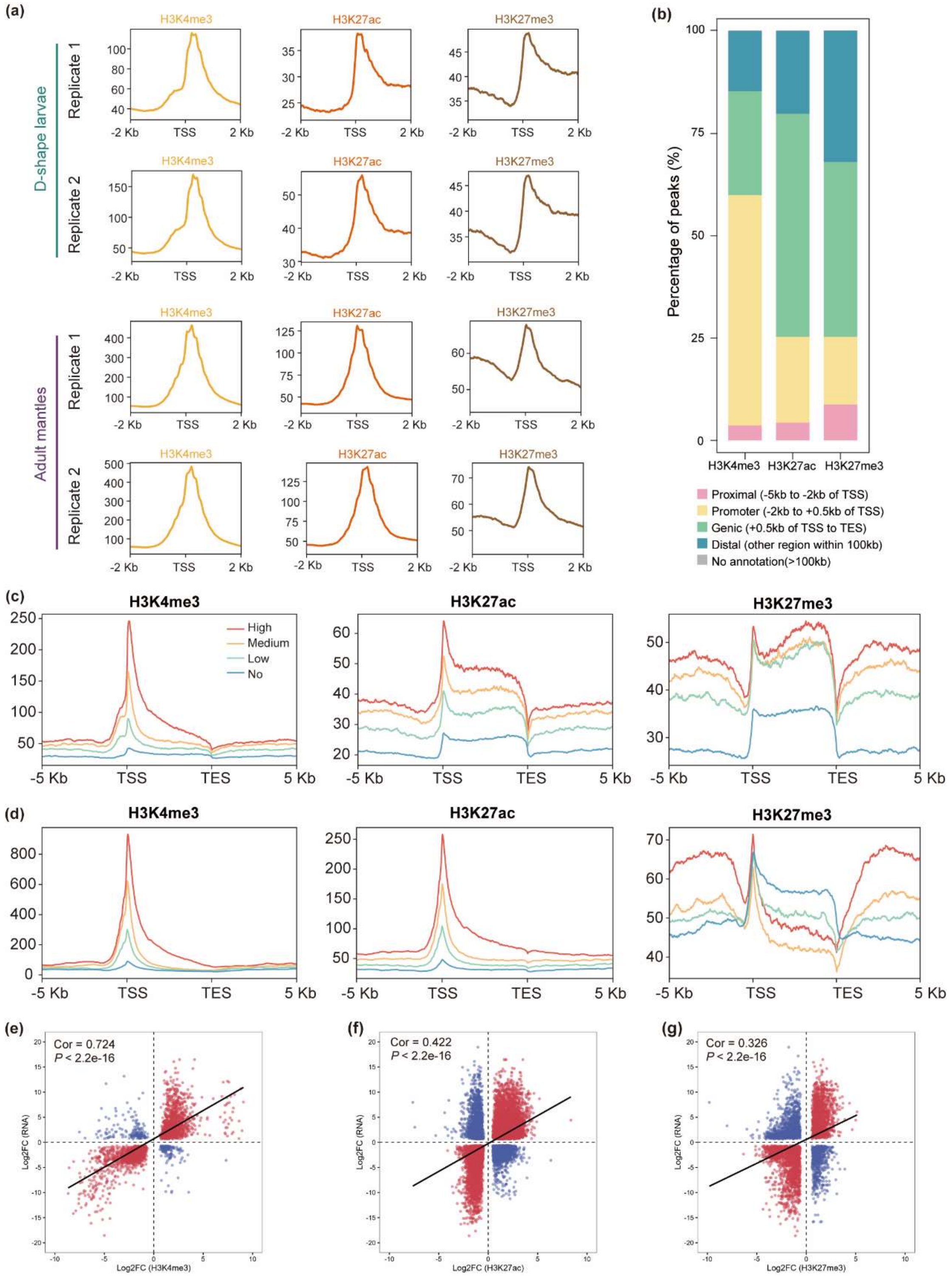

Fig. S9. Genome-wide mapping of three histone marks (H3K4me3, H3K27ac, and H3K27me3) in D-shape larvae and adult mantles of *C. nippona* and their correlation with gene expression patterns. **(a)** Summary plots of three histone mark enrichment around transcription start sites (TSS; ± 2 Kb). **(b)** Genome-wide distribution of peaks

of histone marks. **(c and d)** The levels of three histone mark in genes with different transcription levels in D-shape larvae **(c)** and adult mantles **(d)**. In the analysis, genes were grouped into four categories based on their expression levels: "no" indicates genes with low or no expression (TPM < 1); "low", "medium", and "high" represent the bottom, middle, and top one-third of expressed genes (TPM ≥ 1), respectively. The plots show the distribution of CUT&Tag signals for three histone modifications across these gene expression categories. **(e-g)** The correlation between variation fold changes in gene expression and H3K4me3 **(e)**, H3K27ac **(f)** or H3K27me3 **(g)** levels of genes in D-shape larvae and adult mantles. Pearson correlation coefficients were calculated to assess the linear relationship between the level changes of gene expression and histone modification. Statistical significance was determined using a two-sided Student's t-distribution.

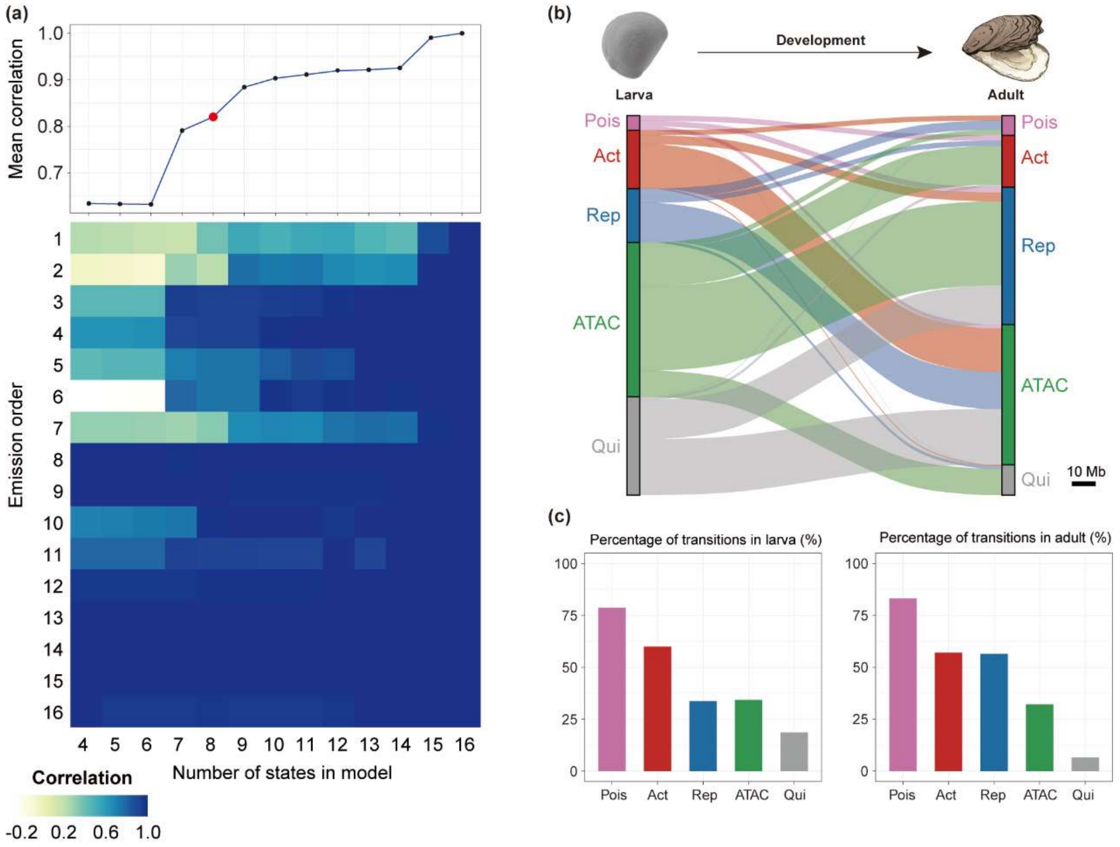

Fig. S10. ChromHMM-inferred genome-wide chromatin states and their transitions from larva to adult stages in *C. nippona*. **(a)** Correlation heatmap of different ChromHMM-trained chromatin state models. The color represents the level of correlation. **(b)** Chromatin state transitions of genes from larva (top) to adult (bottom) stages. **(c)** Top: percentage of chromatin states linked to genes in larvae that undergo transitions to other chromatin state in adults. Bottom: percentage of chromatin states linked to genes in adult that undergo transitions from other chromatin state in larvae.

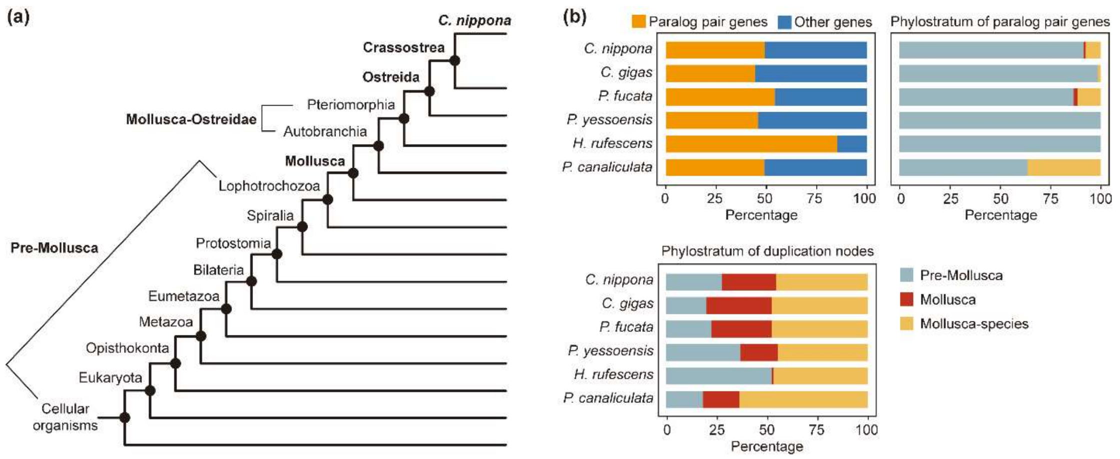

Fig. S11. Gene age of paralogous genes and duplication dating of biomineralization effectors in *C. nippona*. **(a)** Phylogenetically hierarchical classification of gene age in *C. nippona*. **(b)** The proportion of paralogous genes among biomineralization effectors in five molluscs, along with their phylostrata and duplication timing of paralog pairs.

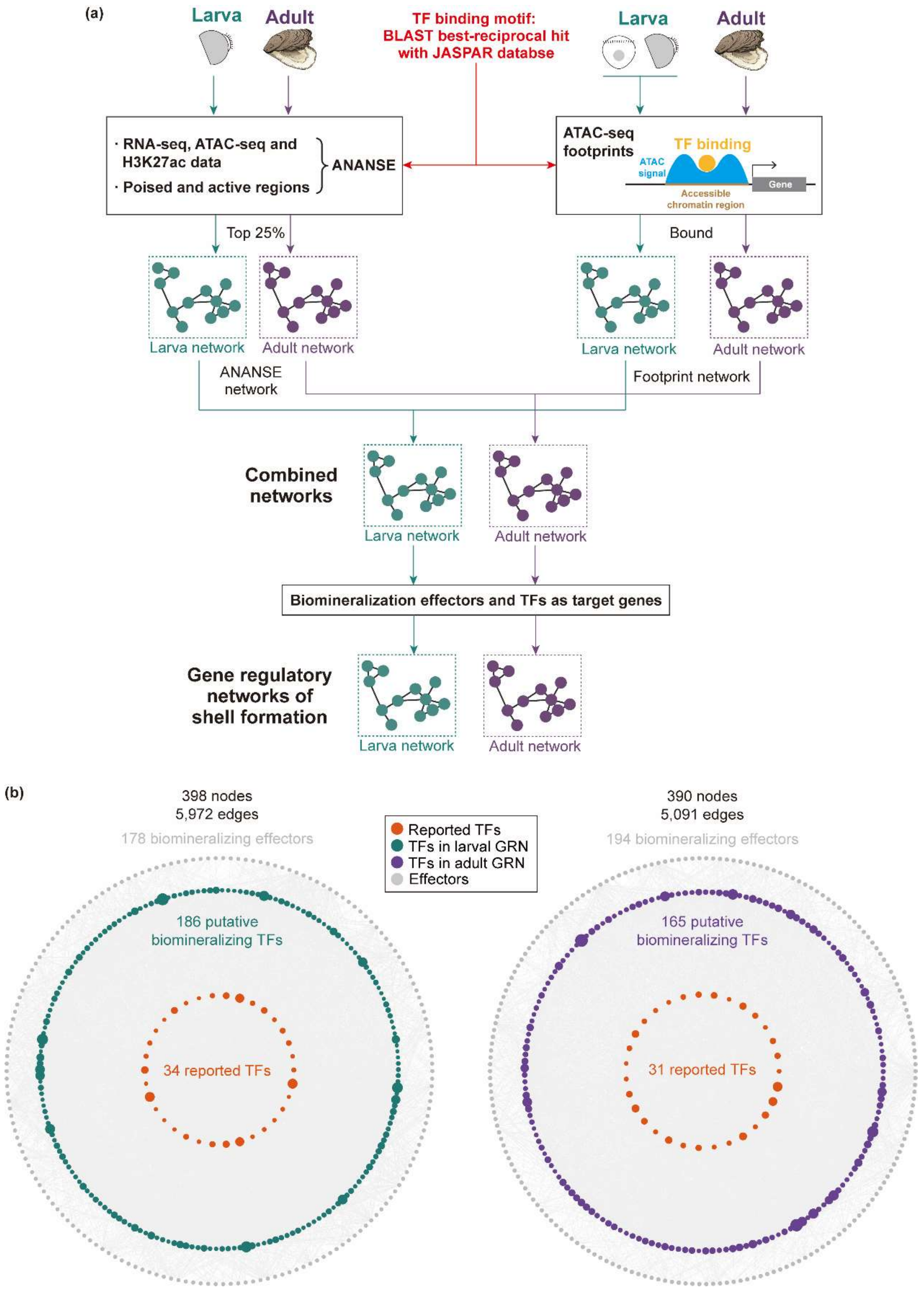

Fig. S12. Construction of gene regulatory networks (GRNs) for larval and adult shell formation in *C. nippona*. **(a)** Schematic pipeline illustrating networks of *cis*-regulatory. **(b)** Overview of GRNs of larval (left) and adult (right) shell formation. Orange dots

represent previously reported TFs, while green (left) and purple (right) dots represent TFs identified in larval and adult GRNs, respectively. Outer grey dots represent biomineralization effector genes. Larval and adult GRNs comprise 398 and 390 nodes, and 5,972 and 5,091 edges, respectively. A total of 220 and 196 putative biomineralization TFs were identified in the larval and adult networks, respectively, including 34 and 31 TFs previously reported in biomineralization. Dot size reflects the outdegree of each node, indicating the number of target genes regulated by a given TF.

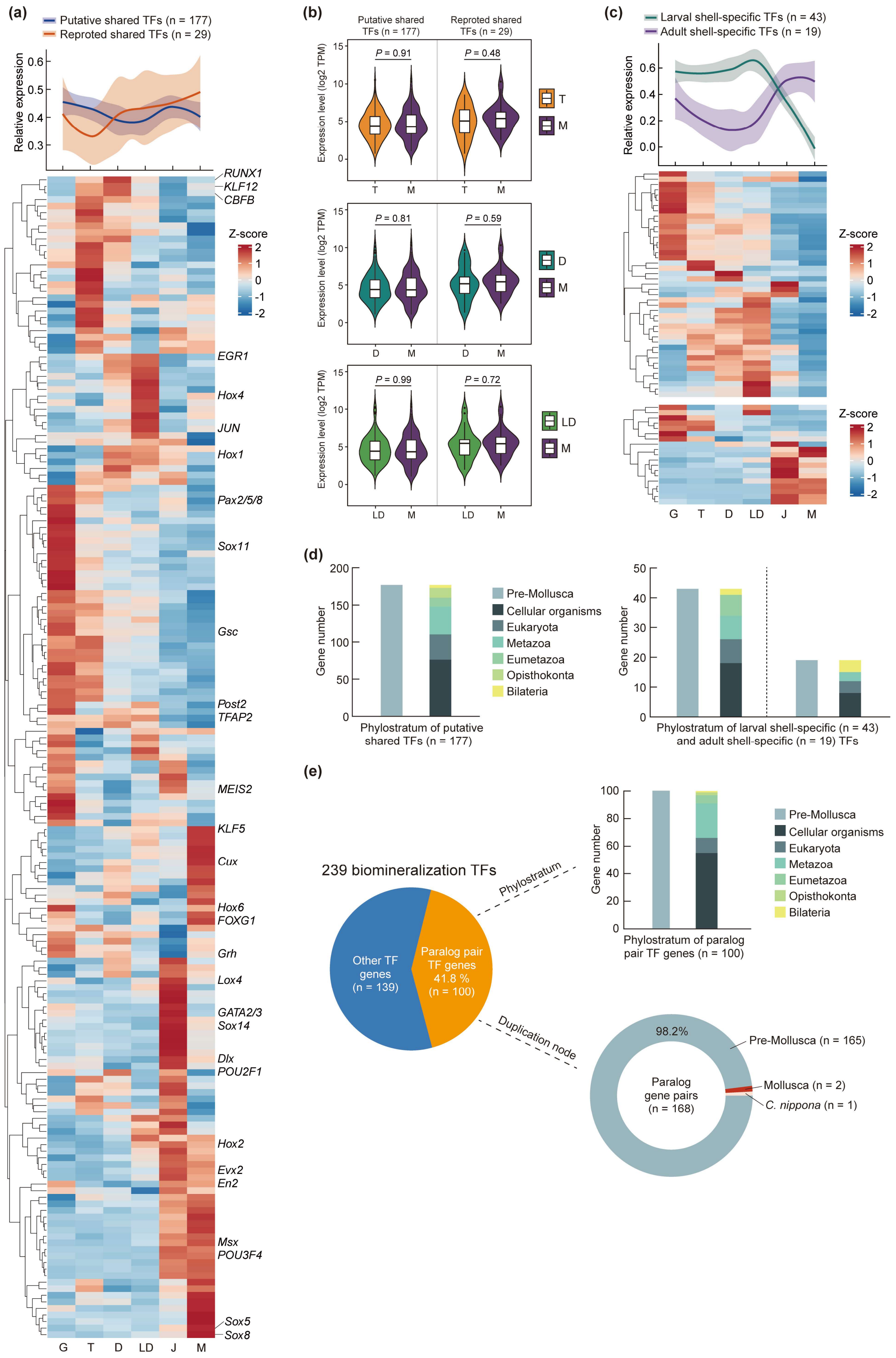

Fig. S13. Gene expression profiles of putative biomineralization TFs in *C. nippona* and their phylostrata. **(a)** Gene expression dynamics (top) of 177 shared TFs (blue)

predicted in both larval and adult GRNs, including 29 previously reported TFs (orange), across six developmental stages in *C. nippona*. Curves are locally estimated scatterplot smoothing (LOESS), colored shaded areas represent standard error of the mean. Gene names of 29 previously reported TFs are labeled in the right of heatmap (bottom). **(b)** Comparison of expression levels for TFs between larva and adult stages. Boxplots include a median with quartiles and outliers above the top whisker. The two-sided Wilcoxon rank-sum test was used to assess significance. **(c)** Gene expression dynamics of larval (green) or adult (purple) shell-specific TFs predicted in larval or adult GRNs, respectively, across six developmental stages in *C. nippona*. Curves are LOESS, colored shaded areas represent standard error of the mean. **(d)** Phylostratum of 177 shared TFs (left) and stage-specific TFs (right). All TFs are of pre-mollusca origin, and further classified as origin from Cellular organisms, Eukaryota, Metazoan, Eumetazoa, Opisthokonta and Bilateria. **(e)** Percentage of paralogous genes among biomineralization TFs, as well as the distribution of their phylostrata and duplication nodes. Abbreviations are the same as supplementary fig. S3.

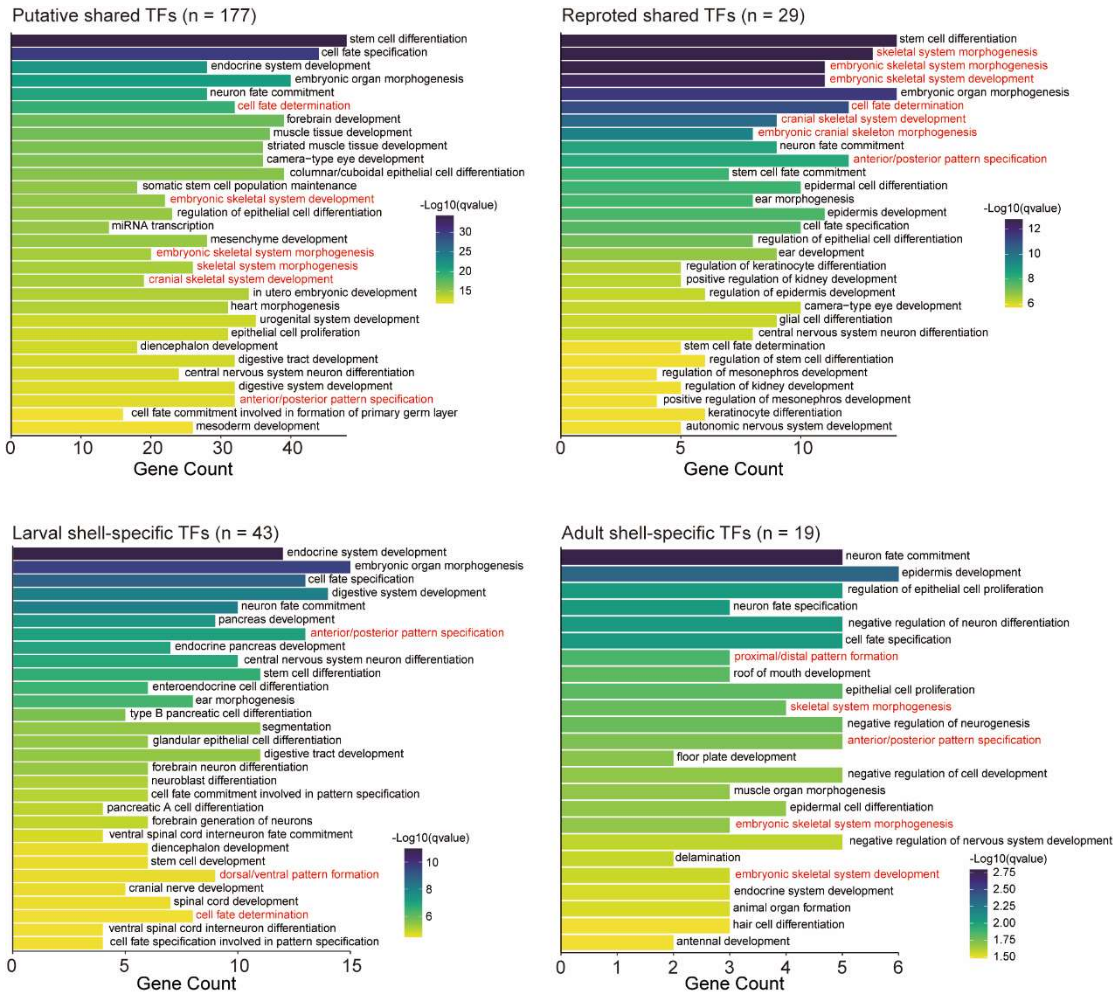

Fig. S14. GO terms enrichment of biomineralization TFs in shell formation GRNs of *C. nippona*. Bar plots depicting gene counts, while colors indicate adjusted *P*-values (qvalue) of the top 30 GO terms for biological process. Terms mentioned in the main text are colored in red.

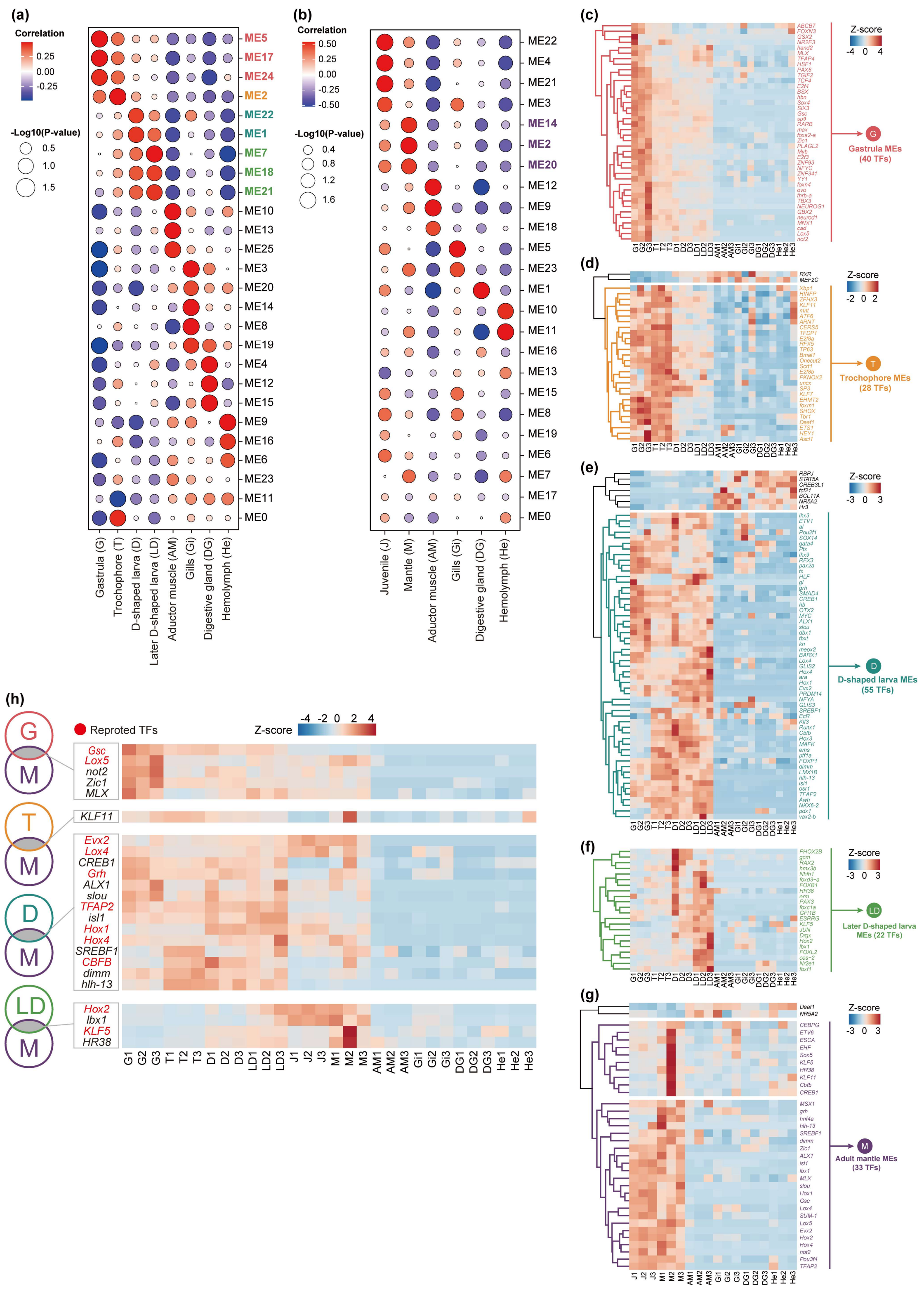

Fig. S15. WGCNA analysis across developmental stages and adult tissues to identify of co-expressed TF genes in both larval and adult shell formation of *C. nippona*. **(a)** Gene modules (MEs) associated with larval shell formation identified by WGCNA

clustering of gene expression profiles of larval stages and non-shell-forming tissues: gastrula (salmon: ME5, ME17 and ME24), trochophore (orange: ME2), D-shape larva (green: ME1 and ME22), and later D-shape larva (medium green: ME7, ME18 and ME21). Dot size indicates the significance (-log10 *P*-value) of the correlation, and the color represents the correlation coefficient. **(b)** MEs associated with adult shell formation in the mantle tissue (purple: ME2, ME14 and ME20), identified through WGCNA clustering of gene expression profiles of juvenile and adult tissues. Correlation and *P*-value are also indicated by dot size and color, respectively. **(c-g)** Heatmaps showing gene expression patterns in MEs associated with larval stages and adult mantle: gastrula **(c)**, trochophore **(d)**, D-shape larva **(e)**, later D-shape larva **(f)**, and adult mantle **(g)**. TFs within MEs were hierarchically clustered to ensure stage- or tissue-specific expression. Genes labeled in black were excluded from the stage- or tissue-specific MEs. **(h)** Co-expressed TFs shared each larval stage and adult mantle, along with their expression profiles across developmental stages and adult tissues. Putative biomineralization TFs reported in previous studies (supplementary table S4) are highlighted in red.

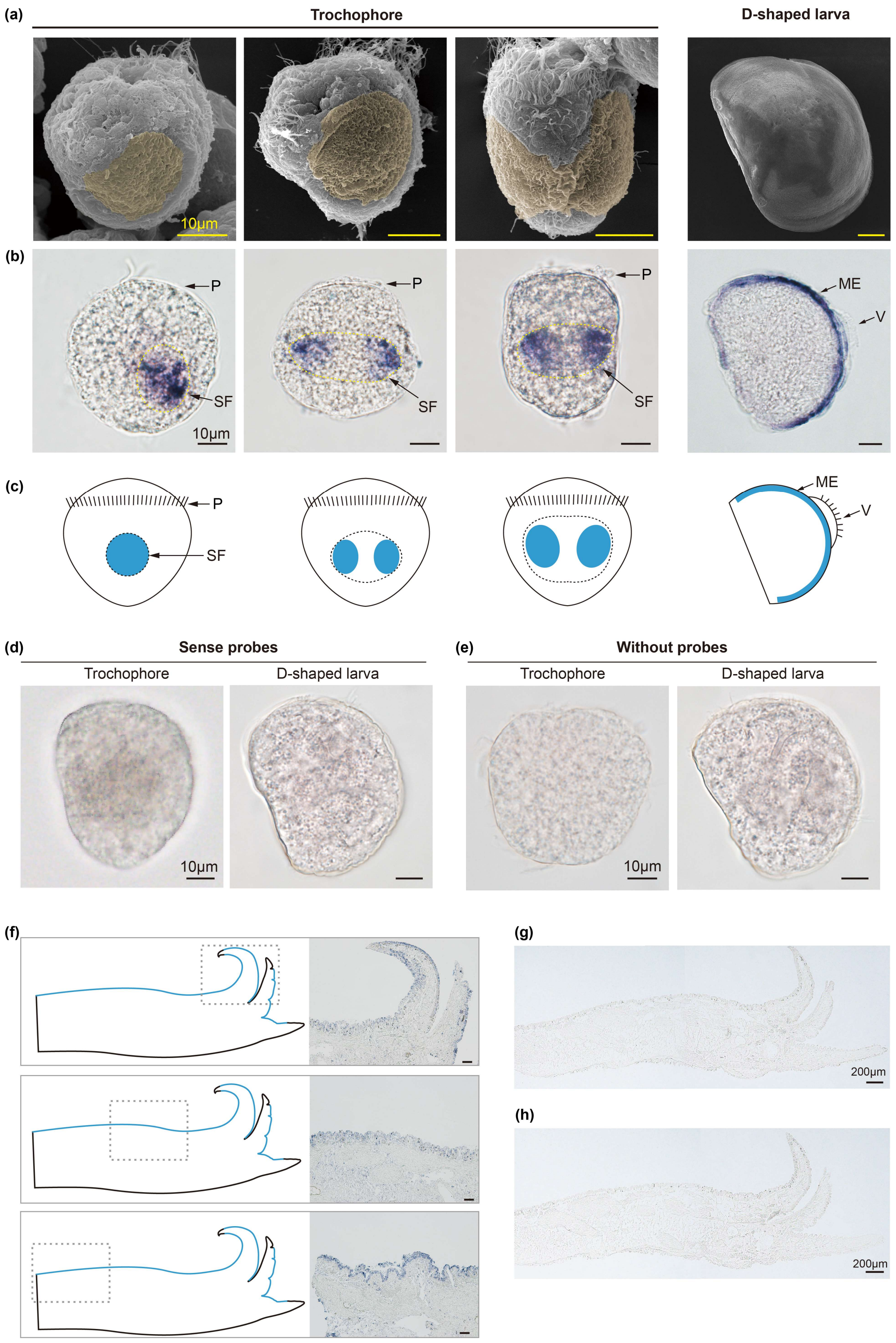

Fig. S16. Spatial expression pattern of the *Hox4* gene in *C. nippona* larvae and adult mantle. **(a)** Scanning electron micrographs (SEMs) of *C. nippona* larvae. Shell field

(SF) in trochophore larvae is highlighted in yellow. Scale bar = 10 μm. **(b and c)** In situ hybridization (ISH) results **(b)** and corresponding schematic illustration **(c)** showing *Hox4* expression (blue) in the SF regions of trochophore larvae and mantle edge (ME) of D-shape larvae. Scale bar = 10 μm. P: prototroch, SF: shell filed, ME: mantle edge, V: veliger. **(d and e)** Negative control experiments for the ISH of D-shape larvae shown in Fig. 5e, using sense probe **(d)** and all hybridization reagents except the probe **(e)**. No detectable signal was observed in the control groups, confirming the reliability of the antisense-probe ISH results. **(f)** Schematic diagram (left) showing the *Hox4* expression (blue) in the adult mantle. The gray dashed boxes indicate the region magnified in the ISH images on the right. Scale bar = 200 μm. **(g and h)** Negative control experiments for the ISH of adult mantle shown in Fig. 5e, using sense probe **(g)** and all hybridization reagents except the probe **(h)**.

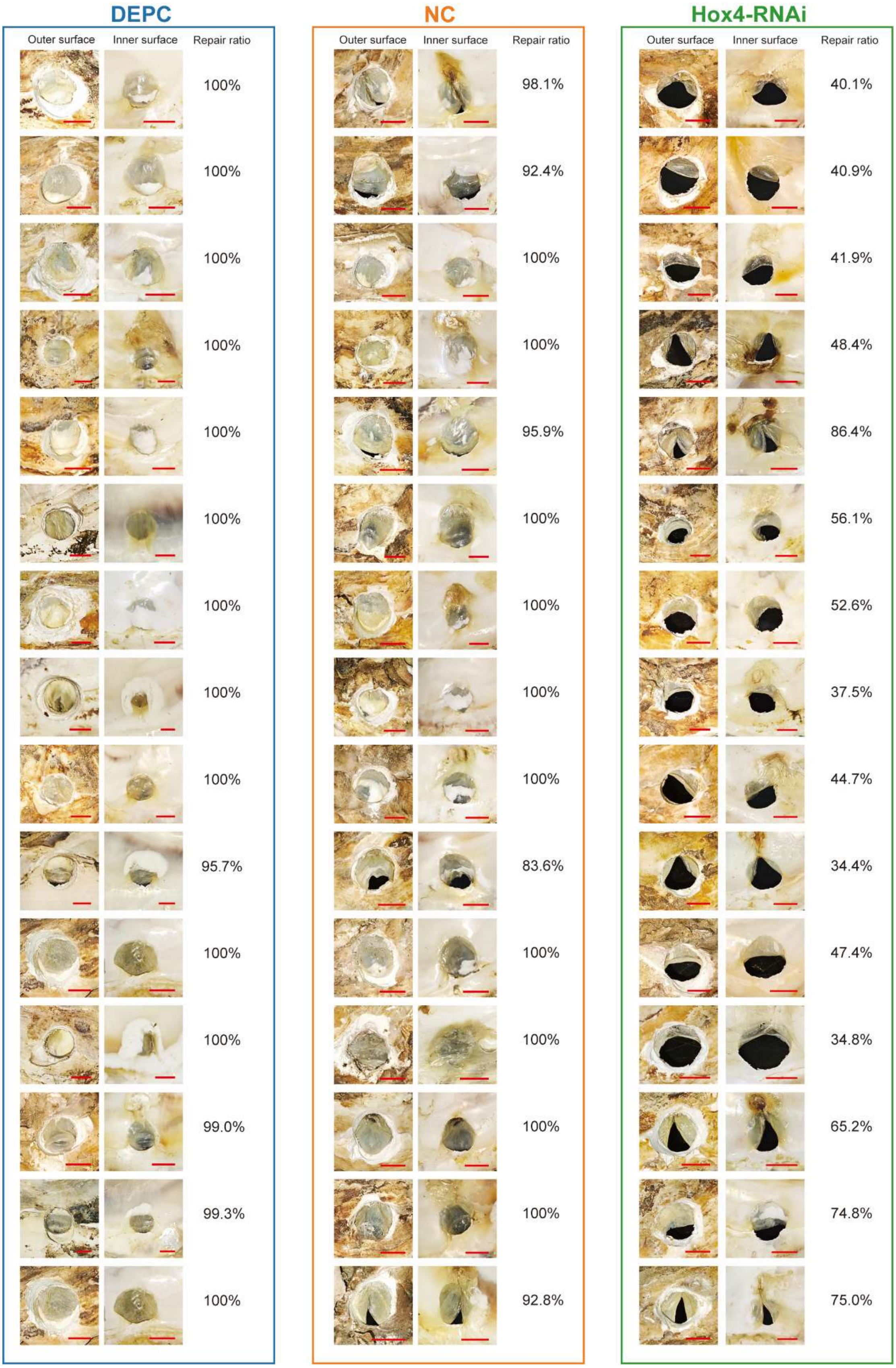

Fig. S17. Bright-field photographs of repaired shells six days after drilling, showing that the drilled holes are completely covered by newly repaired shell in most control

individuals, whereas the repaired area is smaller in *Hox4*-RNAi oysters. Samples are arranged from left to right as DEPC (bule), NC (orange), and *Hox4*-RNAi (green) groups (n = 15 per group). The semi-translucent “new repair membrane” corresponds to newly repaired prismatic and foliated layers, whereas the opaque, milky-white material represents the chalky layer; all of these structures are fully mineralized. Shell repair ratio was assessed as the repaired area divided by the area of the original hole. Scale bar = 2 mm.

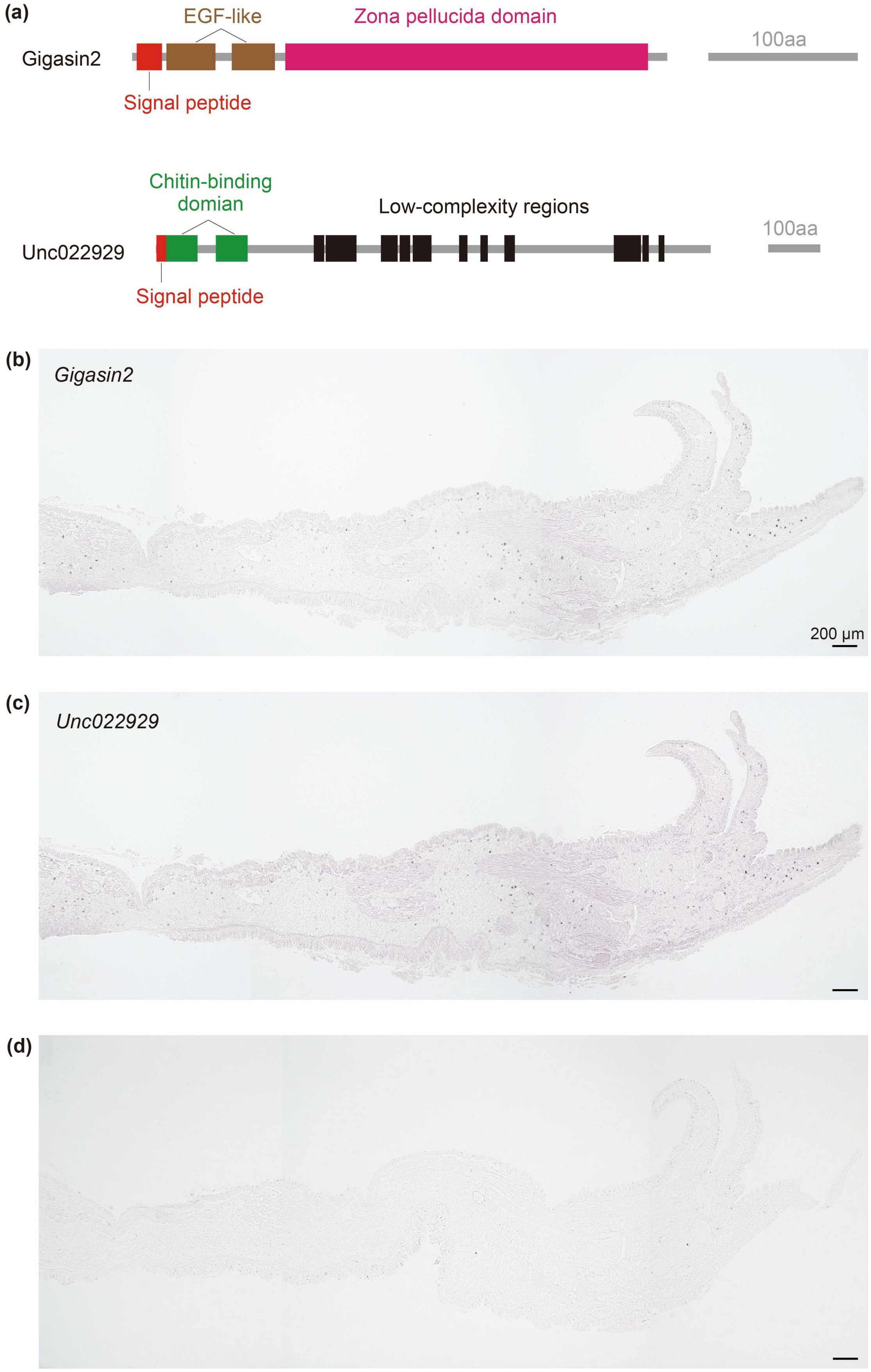

Fig. S18. Negative controls of *Gigasin2* and *Unc022929* ISH signals. (a) Protein

domain architectures of *Gigasin2* and *Unc022929*. Red boxes represent signal peptide domains; brown: EGF-like domains (IPR000742); magenta: zona pellucida domains (IPR001507); green: chitin-binding domains (IPR002557); black: low-complexity regions. **(b-d)** Negative control experiments for the ISH shown in Fig. 5k, using sense probes for **(b)** *Gigasin2* and **(c)** *Unc022929*, and **(d)** all hybridization reagents without probes. Scale bars = 200 μm. No detectable signals were observed in the control groups, confirming the specificity and reliability of the antisense probe signals.

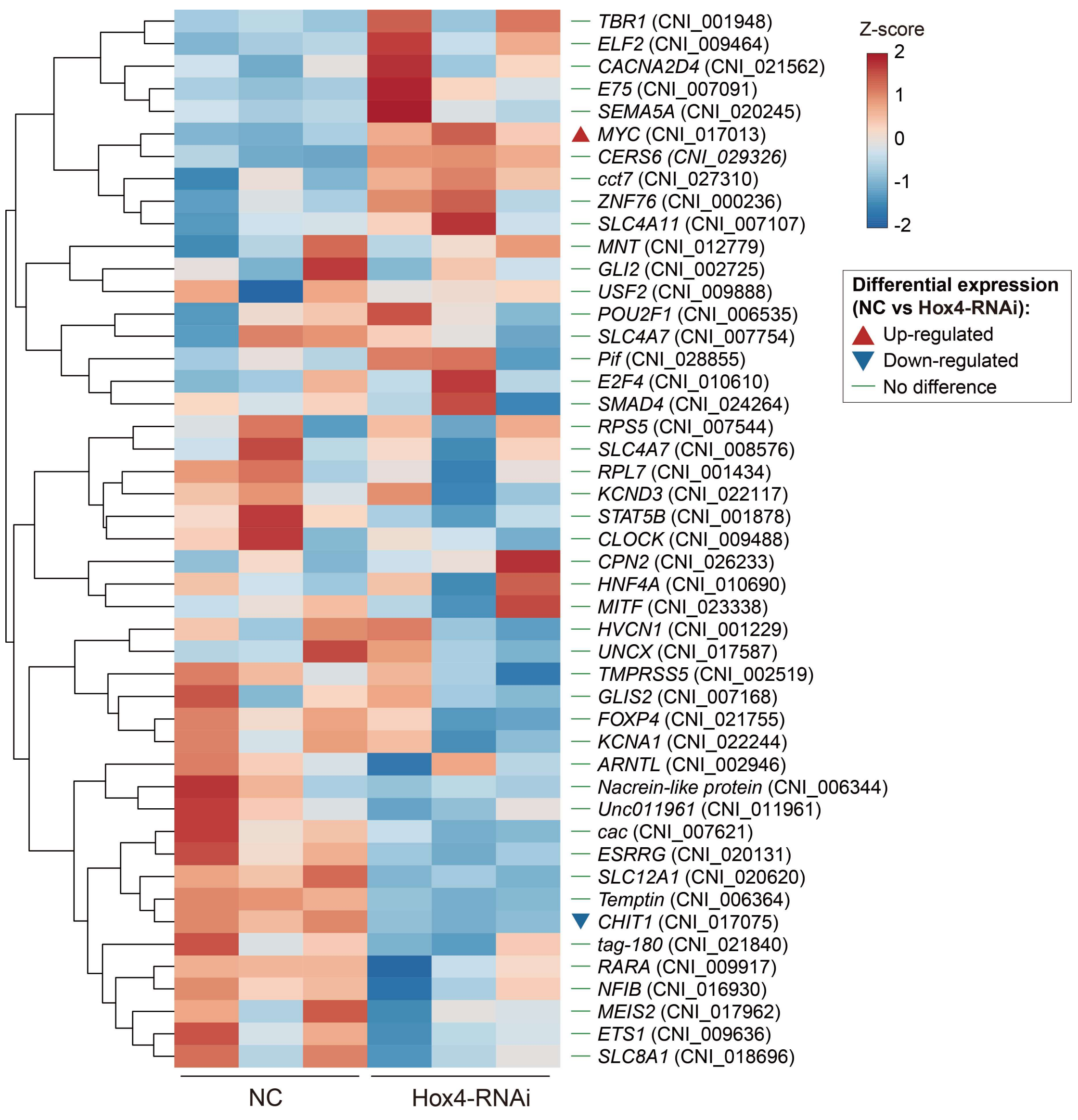

Fig. S19. Heatmap showing expression of *Hox4*-regulated downstream biomineralization genes in the larval GRN after RNAi (n = 3). Differential expression is indicated beside each gene name: upward red triangles denote significantly up-regulated genes after RNAi ($\log_2$foldchange > 1 and FDR < 0.05); downward blue triangles denote significantly down-regulated genes ($\log_2$foldchange < -1 and FDR < 0.05); and green horizontal bars indicate genes without significant changes ($|\log_2$foldchange$|$ < 1 or FDR > 0.05). Five genes (*Pax6*, *BARX1*, *Pif*, *NKX2-1*, and *Unc025400*) were not expressed (TPM < 1) in the adult mantle, and are not shown in

the heatmap. Detailed data are shown in supplementary table S14.

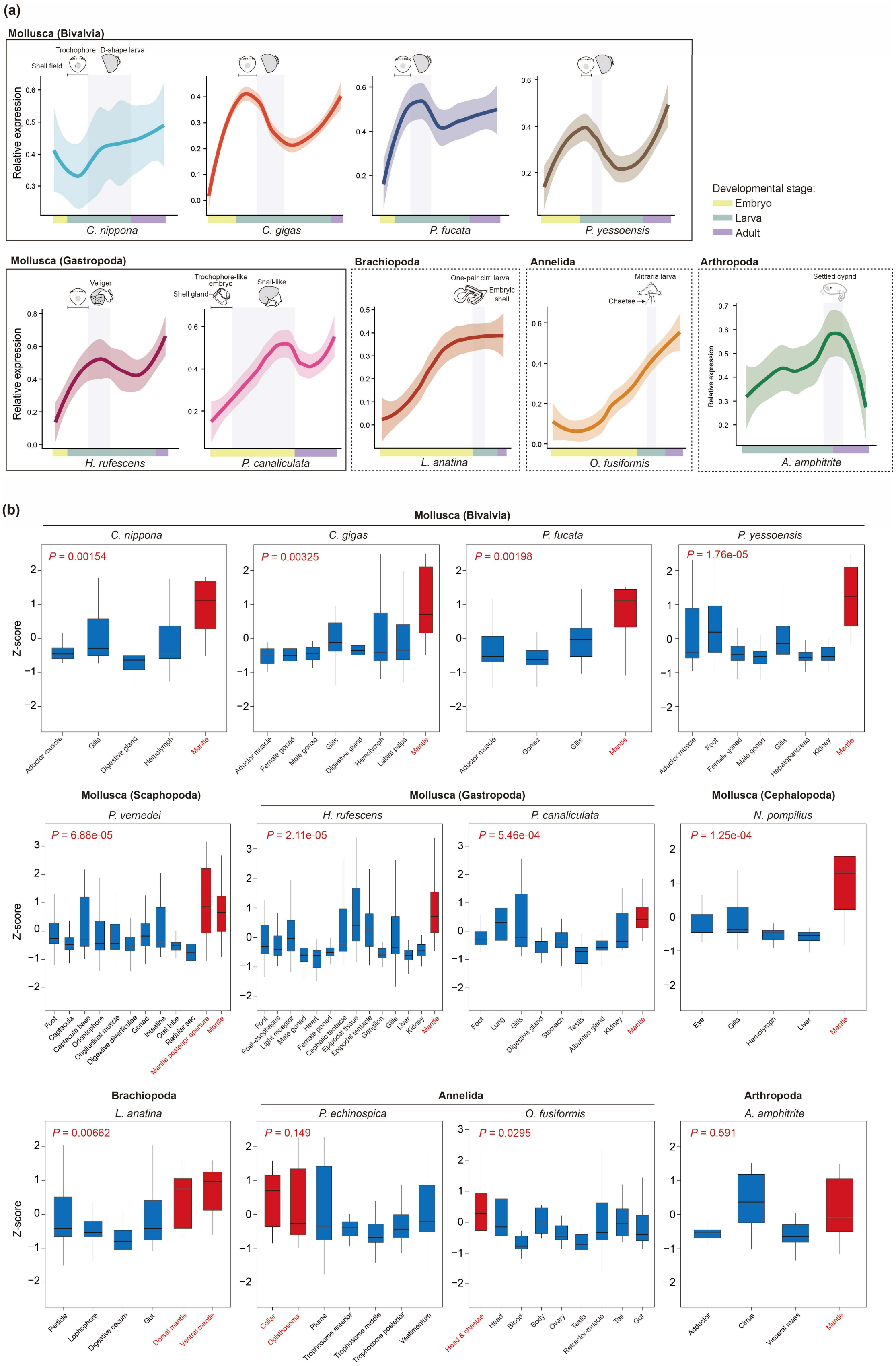

Fig. S20. Gene expression patterns of 29 putative biomineralization TFs shared by the larval and adult shell formation GRNs across developmental stages **(a)** or adult tissues

(b) in bilaterians. Expression curves are fitted using LOESS, with shaded areas indicating the standard error of the mean. In molluscs, the peak expression of these TFs occurs during the trochophore stage, which is a critical period for shell field development. In other lophotrochozoans, the peak expression typically coincides with early exoskeleton-forming larval stages. In contrast, the arthropod *Amphibalanus amphitrite* exhibit peak expression during the late larval stage prior to metamorphosis (settled cyprid). Moreover, in molluscs, as well as in *Lingula anatina* and *Owenia fusiformis*, these TF genes exhibit significantly higher expression levels (two-sided Wilcoxon rank-sum test: $P < 0.05$) in exoskeleton-forming organs. However, no significant tissue-specific expression differences were observed in *Paraescarpia echinospica* and *A. amphitrite*.

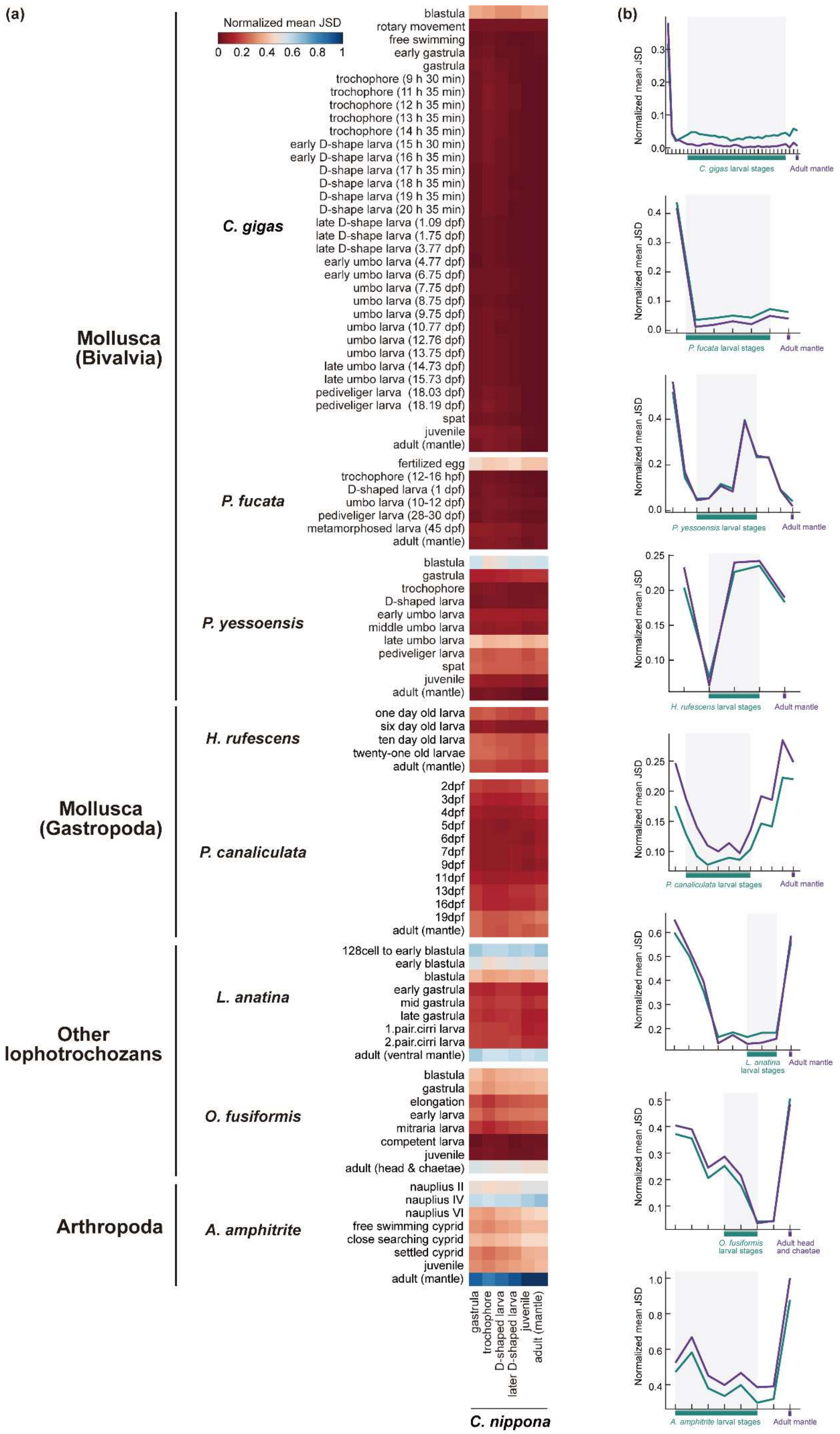

(a)
Normalized mean JSD
0 0.2 0.4 0.6 0.8 1
Mollusca (Bivalvia)
C. gigas
blastula
rotary movement
free swimming
early gastrula
gastrula
trochophore (9 h 30 min)
trochophore (11 h 35 min)
trochophore (12 h 35 min)
trochophore (13 h 35 min)
trochophore (14 h 35 min)
early D-shape larva (15 h 30 min)
early D-shape larva (16 h 35 min)
D-shape larva (17 h 35 min)
D-shape larva (18 h 35 min)
D-shape larva (19 h 35 min)
D-shape larva (20 h 35 min)
late D-shape larva (1.09 dpf)
late D-shape larva (1.75 dpf)
late D-shape larva (3.77 dpf)
early umbo larva (4.77 dpf)
early umbo larva (6.75 dpf)
umbo larva (7.75 dpf)
umbo larva (8.75 dpf)
umbo larva (9.75 dpf)
umbo larva (10.77 dpf)
umbo larva (12.76 dpf)
umbo larva (13.75 dpf)
late umbo larva (14.73 dpf)
late umbo larva (15.73 dpf)
pediveliger larva (18.03 dpf)
pediveliger larva (18.19 dpf)
spat
juvenile
adult (mantle)
P. fucata
fertilized egg
trochophore (12-16 hpf)
D-shaped larva (1 dpf)
umbo larva (10-12 dpf)
pediveliger larva (28-30 dpf)
metamorphosed larva (45 dpf)
adult (mantle)
P. yessoensis
blastula
gastrula
trochophore
D-shaped larva
early umbo larva
middle umbo larva
late umbo larva
pediveliger larva
spat
juvenile
adult (mantle)
Mollusca (Gastropoda)
H. rufescens
one day old larva
six day old larva
ten day old larva
twenty-one old larvae
adult (mantle)
P. canaliculata
2dpf
3dpf
4dpf
5dpf
6dpf
7dpf
9dpf
11dpf
13dpf
16dpf
19dpf
adult (mantle)
Other lophotrochozans
L. anatina
128cell to early blastula
early blastula
blastula
early gastrula
mid gastrula
late gastrula
1.pair.cirri larva
2.pair.cirri larva
adult (ventral mantle)
O. fusiformis
blastula
gastrula
elongation
early larva
mitraria larva
competent larva
juvenile
adult (head & chaetae)
Arthropoda
A. amphitrite
nauplius II
nauplius IV
nauplius VI
free swimming cyprid
close searching cyprid
settled cyprid
juvenile
adult (mantle)
gastrula
trochophore
D-shaped larva
later D-shaped larva
juvenile
adult (mantle)
C. nippona
(b)
Normalized mean JSD
C. gigas larval stages
Adult mantle
P. fucata larval stages
P. yessoensis larval stages
H. rufescens larval stages
P. canaliculata larval stages
L. anatina larval stages
O. fusiformis larval stages
Adult head and chaetae
A. amphitrite larval stages

Fig. S21. Lophotrochozoan larvae share maximal transcriptional similarity of putative biomineralization TFs at early larval stages. **(a)** Heatmaps of normalized Jensen-Shannon divergence (JSD) from pairwise comparisons of 29 single copy one-to-one TF orthologs between *C. nippona* and eight bilaterians. **(b)** Average relative JSD for the stages of minimal divergence to the D-shape larva stage (green) and adult mantle tissues (purple) of *C. nippona* in **(a)**.

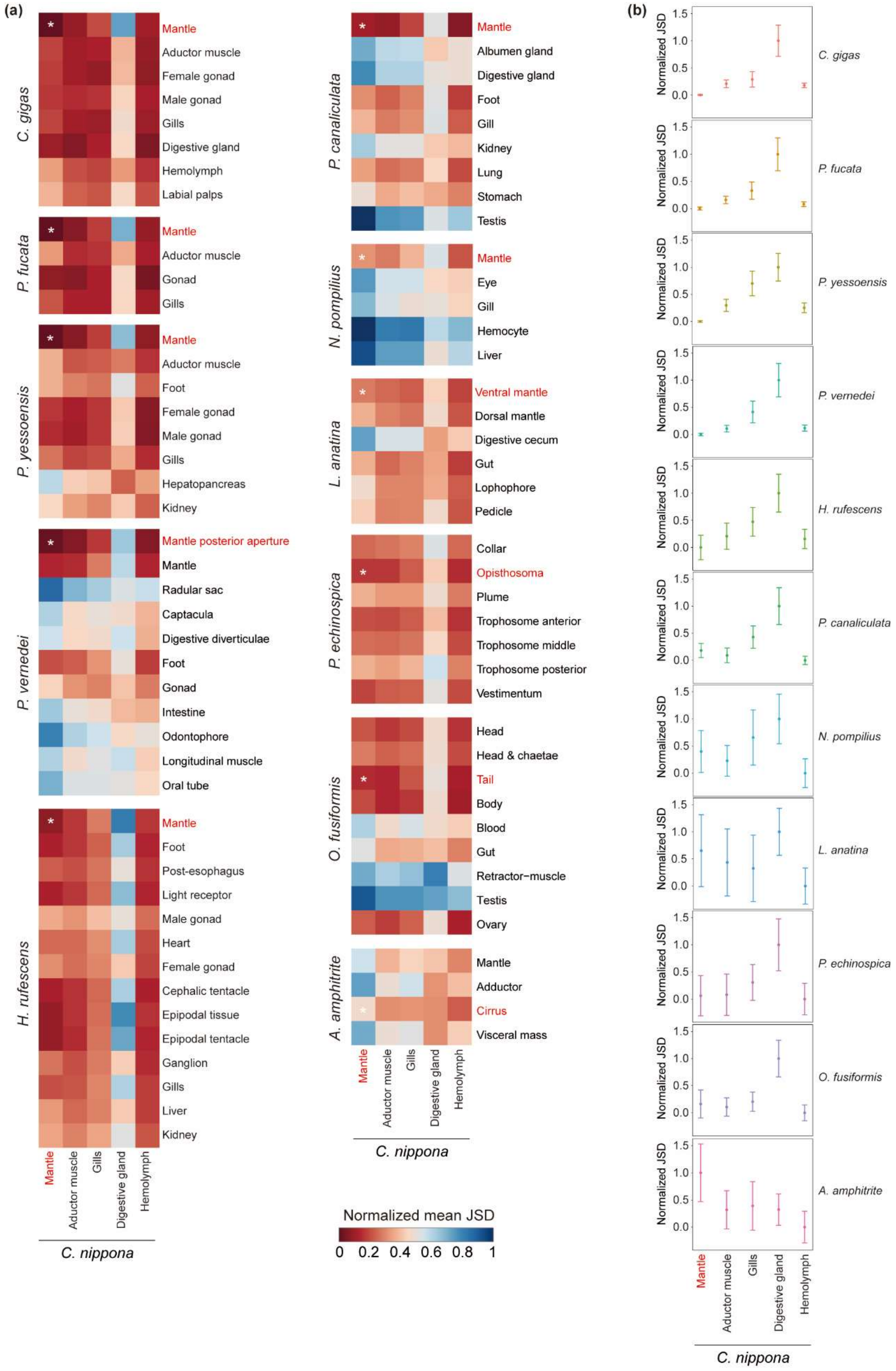

Fig. S22. Molluscan mantles share maximal transcriptional similarity of putative biomineralization TFs. **(a)** Heatmaps of normalized JSD from pairwise comparisons of

29 single copy one-to-one TF orthologs between *C. nippona* and 11 bilaterians. Asterisk indicates the tissue of minimal JSD of each species to the mantle of *C. nippona*. **(b)** Average relative JSD for the datasets shown in Fig. S21a from tissues of minimal JSD to each *C. nippona* tissue. Confidence intervals represent the standard deviation from 1,000 bootstrap replicates of the ortholog sets.

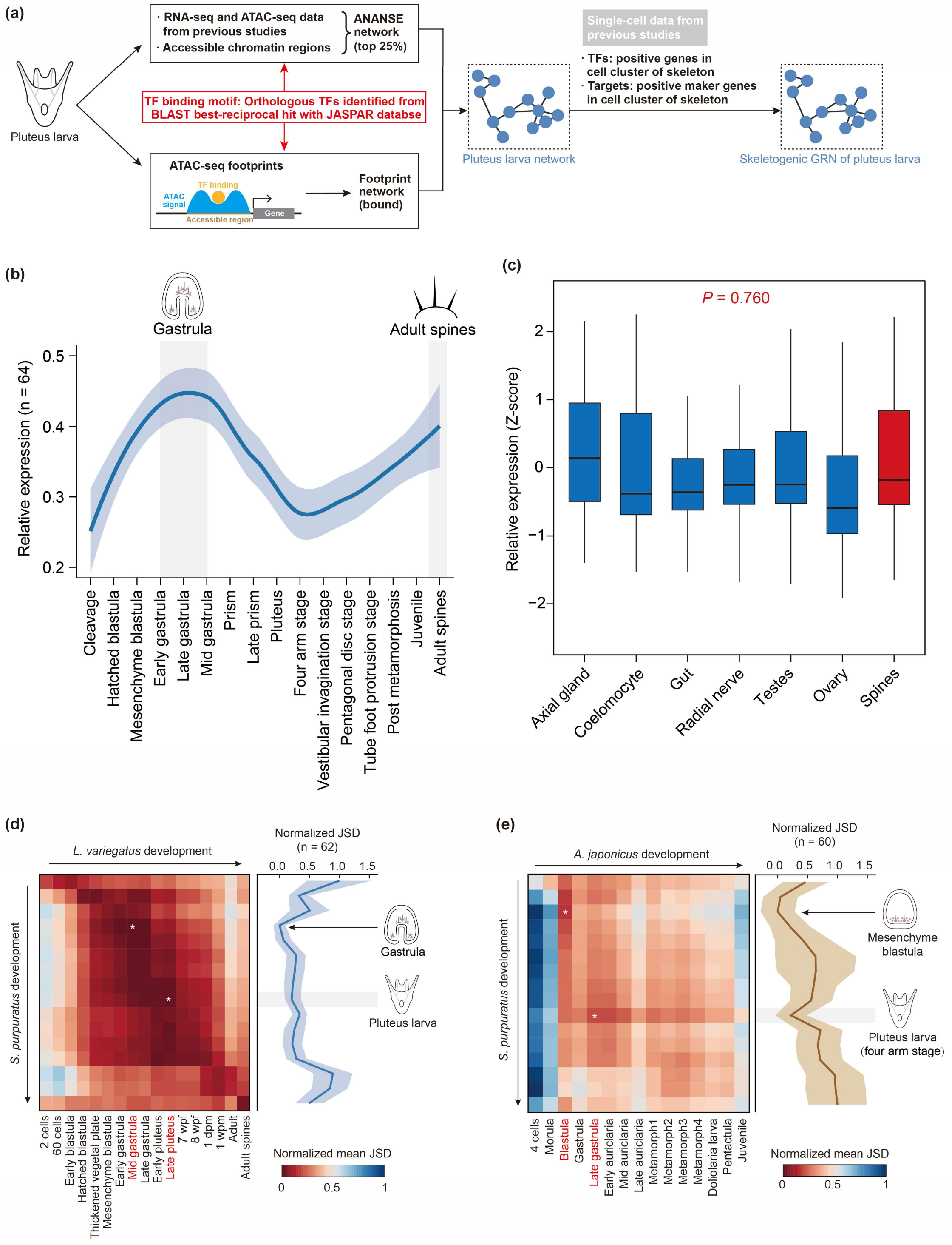

Fig. S23. The sea urchin *Strongylocentrotus purpuratus* shares high transcriptional similarity of putative biomineralization TFs with two other echinoderms during early embryonic and larval development. **(a)** Schematic overview of the pipeline used to reconstruct of skeletogenic GRN in *S. purpuratus* larvae. **(b and c)** Gene expression patterns of 64 putative biomineralization TFs during *S. purpuratus* development **(b)** and

across adult tissues **(c)**. A two-sided Wilcoxon rank-sum test was used to assess significance between expression levels in spines and other tissues. **(d and e)** JSD-based transcriptional similarity of putative biomineralization TFs from pairwise comparisons of *S. purpuratus* with *Lytechinus variegatus* and *Apostichopus japonicus* across developmental stages, respectively. The highest transcriptomic similarity between the two sea urchins occurs at the gastrulation, whereas between *S. purpuratus* and *A. japonicus*, it peaks at the mesenchyme blastula stage. The second-highest similarity stage is indicated with a grey background.

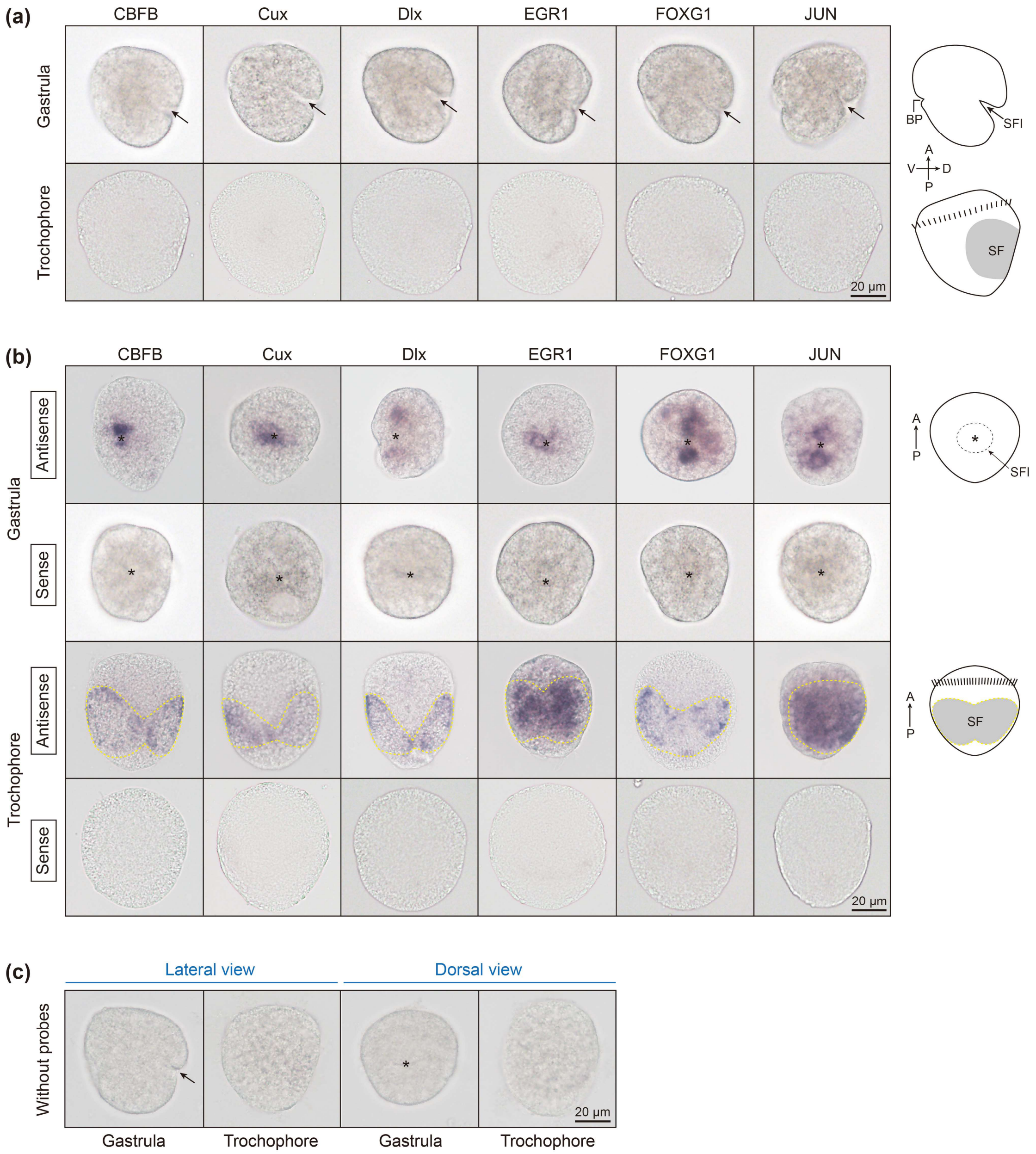

Fig. S24. Representative whole-mount ISH results of six conserved biomineralization-related TFs in the gastrula and trochophore of *C. nippona*. **(a)** Lateral-view sense-probe controls corresponding to Fig. 6d. Schematic diagrams of the gastrula (top) and trochophore (bottom) in lateral view are shown on the right. Arrows indicate the shell field invagination (SFI). A: anterior, P: posterior, V: ventral, D: dorsal, BP: blastopore; SF: shell field. **(b)** Dorsal-view ISH results corresponding to Fig. 6d. During gastrulation, the most part of the shell field (SF) is invaginated and the asterisks indicate

the opening. Yellow dashed lines indicate the shell field region in trochophore. Schematic diagrams of gastrula (top) and trochophore (bottom) in dorsal view are shown on the right. A: anterior, P: posterior, SFI: shell field invagination. **(c)** No-probe control containing all hybridization reagents except the probe. Arrows or asterisks indicate the SFI. No detectable signal was observed in either the sense-probe controls or the no-probe control, supporting the specificity and reliability of the antisense-probe ISH results.

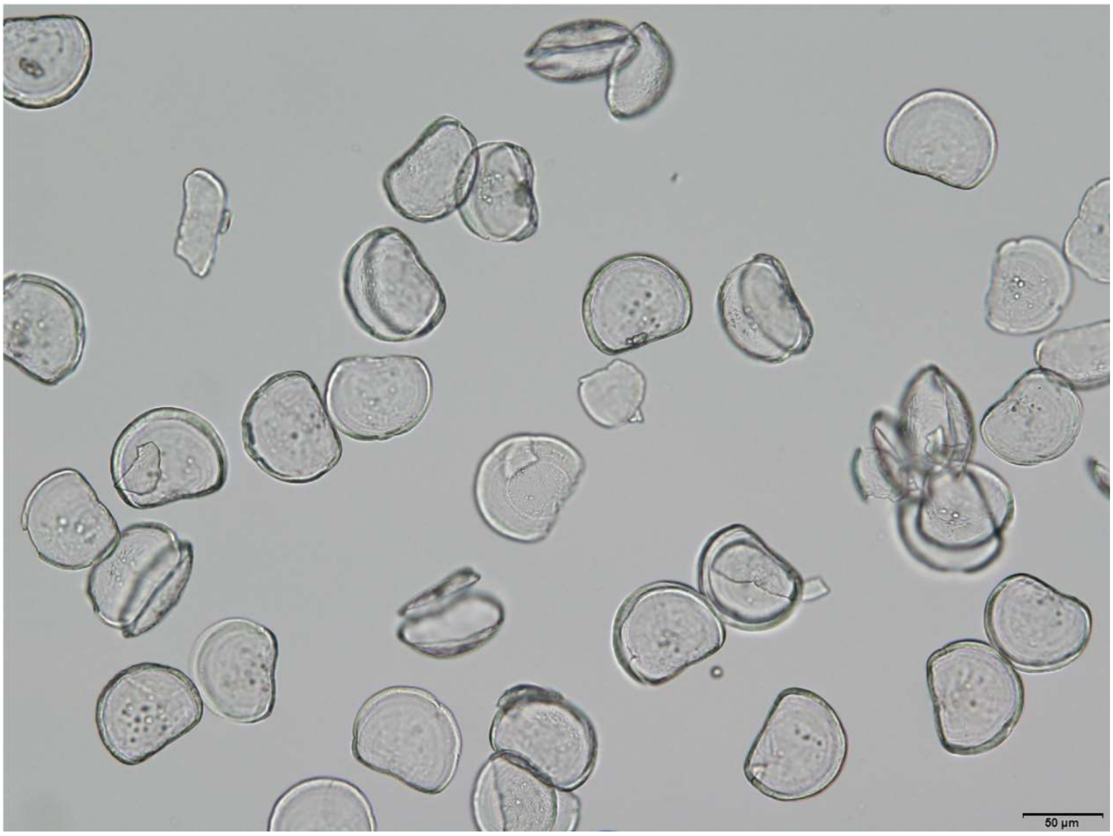

Fig. S25. Larval shells after cleaning.

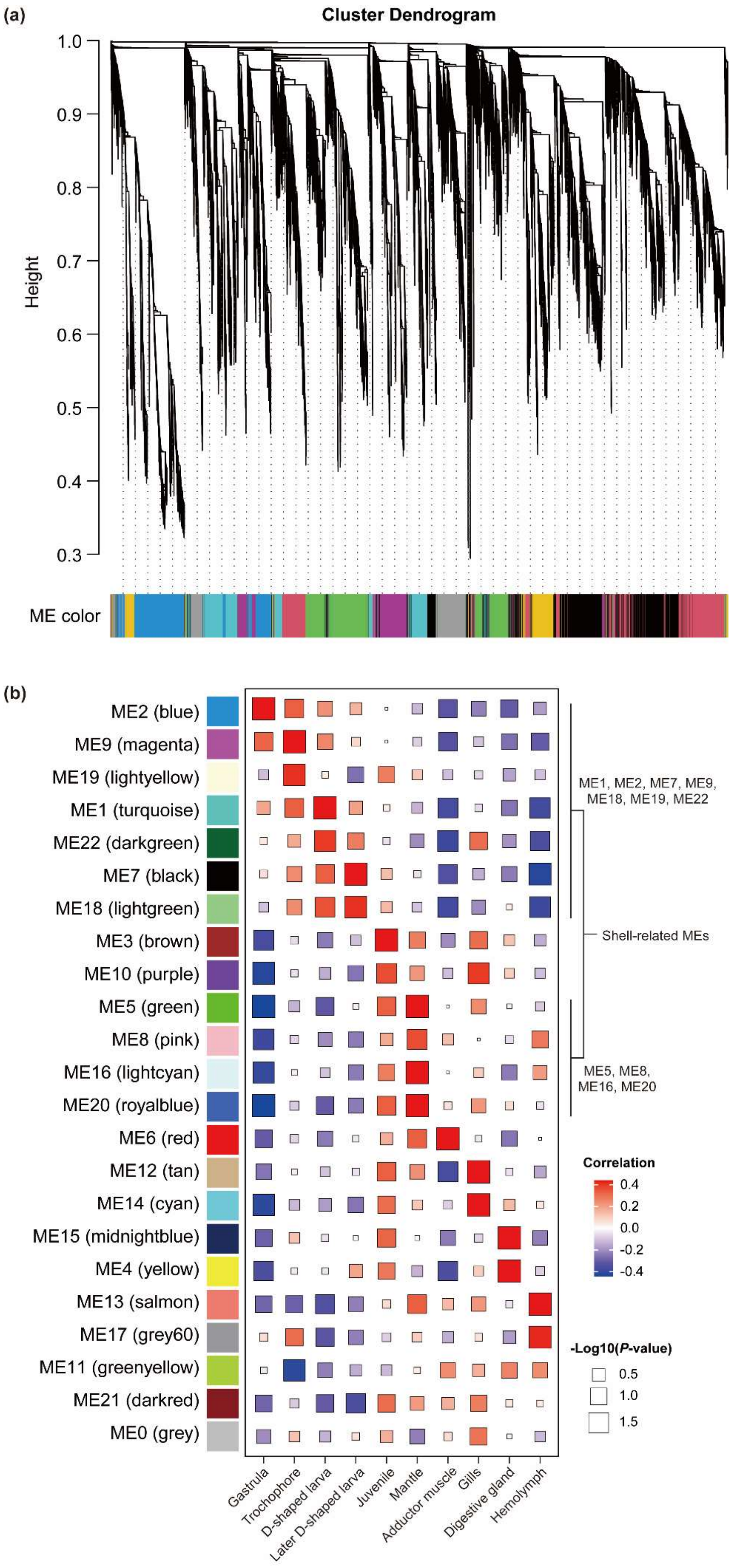
(a)
Cluster Dendrogram
Height
1.0
0.9
0.8
0.7
0.6
0.5
0.4
0.3
ME color
(b)
ME2 (blue)
ME9 (magenta)
ME19 (lightyellow)
ME1 (turquoise)
ME22 (darkgreen)
ME7 (black)
ME18 (lightgreen)
ME3 (brown)
ME10 (purple)
ME5 (green)
ME8 (pink)
ME16 (lightcyan)
ME20 (royalblue)
ME6 (red)
ME12 (tan)
ME14 (cyan)
ME15 (midnightblue)
ME4 (yellow)
ME13 (salmon)
ME17 (grey60)
ME11 (greenyellow)
ME21 (darkred)
ME0 (grey)
ME1, ME2, ME7, ME9, ME18, ME19, ME22
Shell-related MEs
ME5, ME8, ME16, ME20
Correlation
0.4
0.2
0.0
-0.2
-0.4
-Log10(P-value)
0.5
1.0
1.5
Gastrula
Trochophore
D-shaped larva
Later D-shaped larva
Juvenile
Mantle
Adductor muscle
Gills
Digestive gland
Hemolymph

Fig. S26. WGCNA analysis of expressed genes (TPM > 1) across developmental stages and adult tissues in *C. nippona*. **(a)** Module construction of gene-expression network for 30 samples. **(b)** Correlation matrix between MEs and developmental stages or adult organs. MEs associated with larval stages (ME1, ME2, ME7, ME9, ME18, ME19, and ME22) and the adult mantle (ME5, ME8, ME16, and ME20) contain biomineralization effector genes that are considered to be involved in shell formation (correlation valve > 0 and *P* value < 0.05).

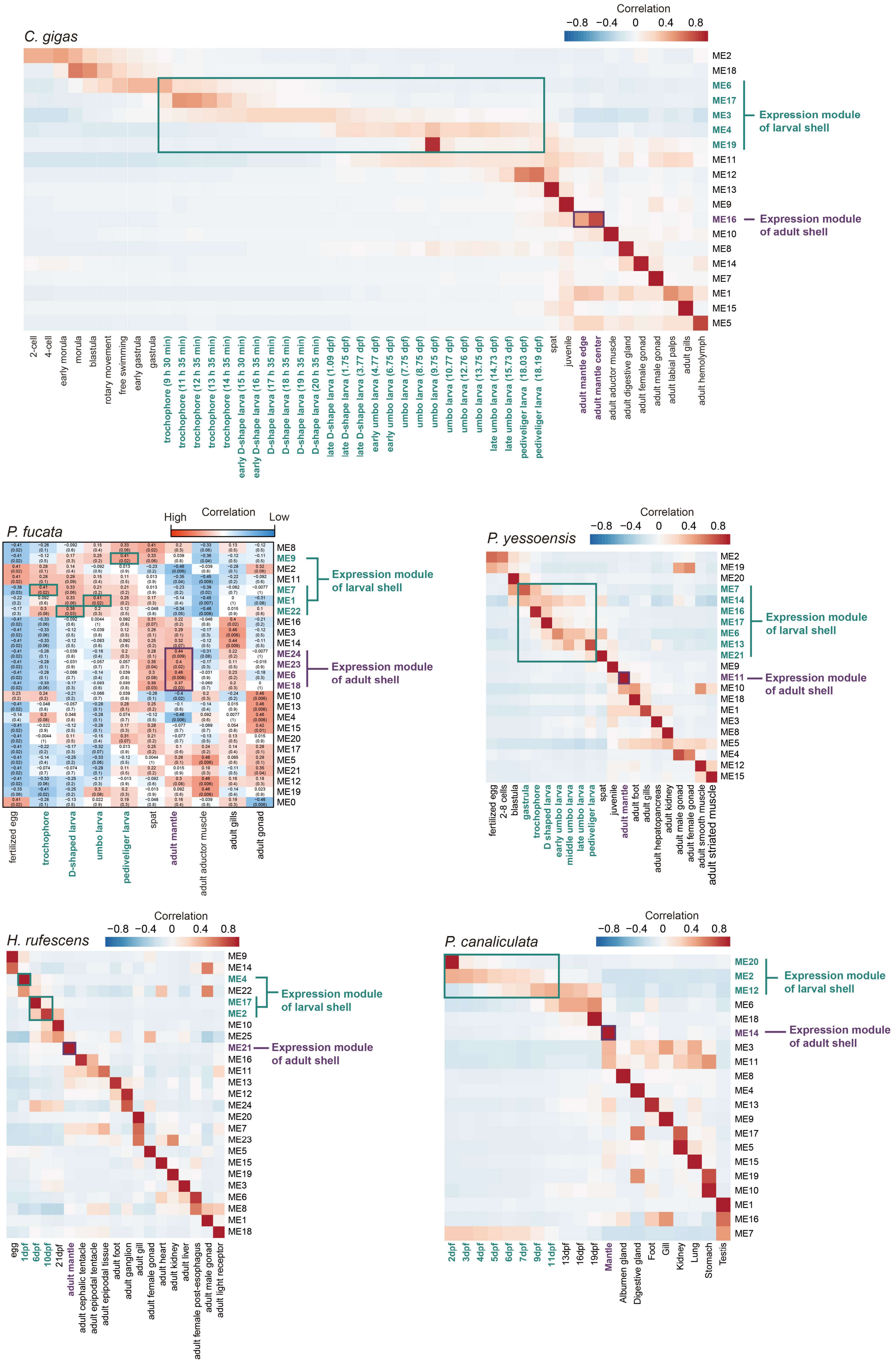

Fig. S27. WGCNA analysis of expressed genes (TPM > 1) across developmental stages and adult tissues in five other molluscs. MEs associated with larval stages (green) and

the adult mantle (purple) contain biomineralization effector genes that are considered to be involved in shell formation.

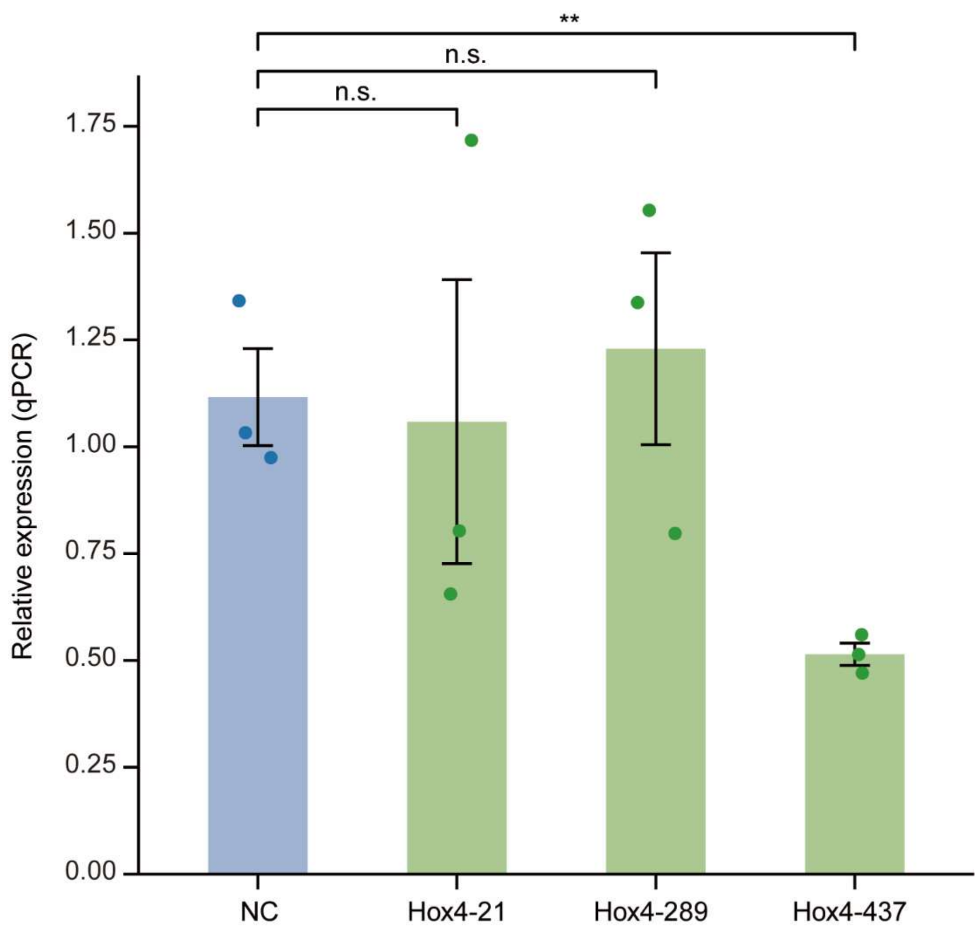

Fig. S28. Results of the RNA interference (RNAi) pilot experiment. Three small interfering RNA (siRNA) strands targeting *Hox4* were tested by measuring the expression level of *Hox4* following the RNAi procedure described in the Methods section of the main text. The results indicated the siRNA strand Hox4-437 was the most effective one. Real-time quantitative polymerase chain reaction (RT-qPCR) was performed using cDNA from mantle tissues of *C. nippona* individuals injected with siRNA three times over a six-day period following shell-drilling (n = 3; mean ± SE; two-sided Student's t-test: $**P < 0.01$; n.s., no significance).

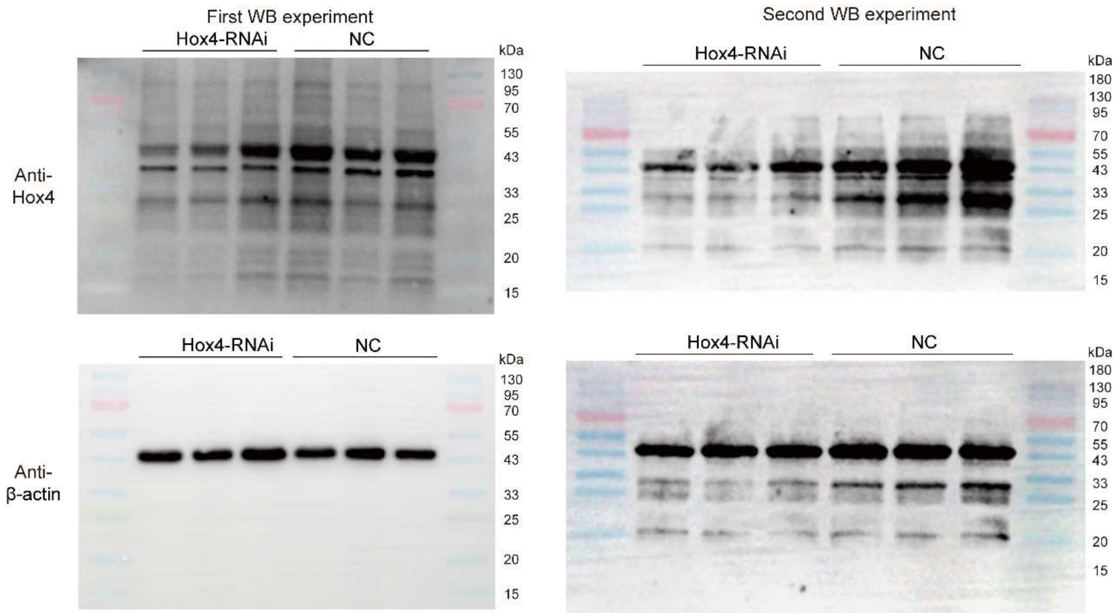

Fig. S29. Western blot (WB) analysis of Hox4 protein abundance in the mantle tissues (n = 3) after RNAi experiment. The experiment was repeated twice and yielded consistent results (supplementary table S27). For each replicate, the same membrane was sequentially incubated with anti-Hox4 and anti-β-actin antibodies after washing.
