# Supplementary material for: Evolutionary innovation within conserved gene regulatory networks underlying biomineralized skeletons in Bilateria
Source: Mol Biol Evol. 2026 Jan 20;43(2):msag019. doi: 10.1093/molbev/msag019 (PMC12862220; doi:10.1093/molbev/msag019)
Supplement: msag019_Supplementary_Data [file msag019_supplementary_data.zip › Supplementary notes.pdf]

SUPPLEMENTARY NOTES

Evolutionary innovation within conserved gene regulatory networks underlying biomineralized skeletons in Bilateria

Yitian Bai[1 †], Yue Min[1 †], Shikai Liu[1 †], Yiming Hu[1], Shulei Jin[1], Hong Yu[1], Lingfeng Kong[1], Daniel J. Macqueen[3], Shaojun Du[4] and Qi Li[1,2*]

[1]Key Laboratory of Mariculture, Ministry of Education, Ocean University of China, Qingdao 266003, China

[2]Laboratory for Marine Fisheries Science and Food Production Processes, Qingdao Marine Science and Technology Center, Qingdao 266237, China

[3]The Roslin Institute and Royal (Dick) School of Veterinary Studies, The University of Edinburgh, Midlothian, UK

[4]Institute of Marine and Environmental Technology, Department of Biochemistry and Molecular Biology, University of Maryland School of Medicine, Baltimore, MD, USA

[†] These authors contributed equally.

*Correspondence: qili66@ouc.edu.cn

Supplementary note 1: Improvement of gene annotation

A new full-length transcriptome (mixed stages, mRNA) was generated from six shell-forming stages/tissues (gastrula, trochophore, D-shaped larva, later D-shaped larva, juvenile, and adult mantle) using Iso-seq on the PacBio Sequel II platform. From 28,238,173 raw subreads, a total of 458,171 circular consensus sequence (CCS) reads were obtained (supplementary fig. S2). After filtering, 348,493 high-quality full-length reads were retained. Of these, 346,373 reads were mapped to the *C. nippona* genome. Transcript models were subsequently reconstructed, collapsed to remove redundancy, and merged with our previously published Iso-seq dataset (Bai, et al. 2023). Low-support isoforms were filtered out, and the resulting transcript models were further refined using short-read RNA-seq data and rule-based filtering to enhance annotation accuracy and confidence. In total, 89,768 high-confidence isoforms were identified, of which 85,575 were predicted to encode proteins. BUSCO analysis (Manni, et al. 2021) of these protein-coding isoforms using the metazoan dataset (n=954) revealed 81.2% completeness (28.0% single-copy, 53.2% duplicated), with 1.4% fragmented and 17.4% missing BUSCOs, indicating a high level of transcript completeness. These isoforms were then merged with gene models from the MAKER annotation using AGAT (v.1.3.0) (https://github.com/NBISweden/AGAT), resulting in a final non-redundant gene annotation containing 29,577 gene models (supplementary table S1).

Among the 29,577 predicted gene models, 28,663 were identified as protein-coding genes and 914 as non-coding genes, with an average gene length of 10,641 bp

(supplementary table S1). The total number of predicted genes is comparable to that of our previous annotation, while both the average gene length (10,641 bp) and CDS length (1,521 bp) in this study are longer than those of the previous annotation (9,005 bp and 1,476 bp, respectively) (Bai, et al. 2023). In addition, we annotated a total of 117,732 isoforms, strongly supported by full-length transcript evidence (supplementary table S1). Importantly, the completeness of the predicted protein-coding gene set reached 96.4% and 96.6% for metazoan and molluscan orthologs, respectively (supplementary fig. 3a), and 99.66% of protein-coding genes annotated by at least one public database (supplementary fig. 3b). These results collectively highlight the notable improvement and high quality of the gene annotation achieved in this study.

Supplementary note 2: GO enrichment of each gene cluster

K-means clustering was performed on the full transcriptome dataset to identify potential gene sets and biological processes related to specific developmental stages, yielding ten gene clusters that broadly corresponded to the major developmental stages (Fig. 2a). Gene ontology (GO) enrichment analyses of these clusters revealed distinct biological programs underpinning each cluster and developmental stage (supplementary fig. S5 and supplementary table S3).

Clusters C1 and C2 were predominantly associated with the gastrula stage, encompassing the maternal-to-zygotic transition and rapid cell proliferation. Cluster C1 was enriched for GO terms related to mRNA processing, RNA splicing, and translational initiation, as well as histone modification and chromatin remodeling, consistent with a role in maternal mRNA clearance and zygotic genome activation. Cluster C2 showed enrichment in DNA replication, chromosome segregation, and double-strand break repair, reflecting the mitotic activity during embryonic development. These patterns are consistent with transcriptomic dynamics previously reported in *Mytilus galloprovincialis* (Miglioli, et al. 2024).

Cluster C3, highly expressed during the trochophore stage, was enriched for glycoprotein biosynthesis, endoplasmic reticulum stress response, and vesicle transport, reflecting the larval organogenesis, cuticle formation, and epithelial morphogenesis. In contrast, Cluster C4, marking the transition from trochophore to D-larva, was

dominated by genes involved in cilium assembly, intraciliary transport, and axoneme organization, consistent with the development of motile and sensory cilia essential for larval movement and environmental sensing. Clusters C5 and C6, specific to the D-shaped larval stages, exhibited functional signatures linked to both metabolism and shell formation. These included GO terms related to lipid oxidation, peroxisomal transport, and motile cilium assembly, suggesting a coupling between metabolic energy production and larval motility. In addition, genes within cluster C5 and C6 were associated with ribosomal structure, extracellular matrix and calcium ion transport, supporting roles in protein biosynthesis (e.g., for shell matrix proteins) and ion regulation during larval shell formation. These transcriptional signatures closely parallel those identified in *Patinopecten yessoensis* (Wang, et al. 2020), in which gene modules upregulated during the trochophore and D-larval stages were enriched for processes related to ciliary motilityand shell formation, including chitin metabolism and calcium ion binding. These observations suggest the existence of a conserved developmental framework among bivalves, wherein the coordinated activation of gene regulatory programs governing larval motility and biomineralization facilitates the construction of larval shell during early ontogeny.

At the juvenile stage, clusters C7 and C8 were associated with benthic life and immune activation. C7 was characterized by GO terms related to chitin metabolism and extracellular matrix organization, likely supporting adult shell formation. C8 showed enrichment for antiviral defense responses, such as leukocyte differentiation,

lymphocyte activation, and response to virus, indicating an activation of innate immunity following settlement. Clusters C9 and C10, predominantly expressed in the adult mantle, were enriched in actin cytoskeleton organization, Rho GTPase signaling, actomyosin structure formation, and striated muscle development. These functions are likely associated with tissue homeostasis and functions involved in mantle activity and adult shell formation.

In summary, these findings support the delineation of six major developmental stages in the oyster ontogeny, each characterized by a distinct combination of anatomical features and transcriptional programs. The stage-specific enrichment of GO terms highlights both evolutionarily conserved processes, such as ciliogenesis and innate immunity, and Mollusca-specific innovations, such as peroxisomal energy metabolism during larval shell formation and cytoskeletal remodeling in the adult mantle. These features likely contribute to ecological resilience and morphological diversification in molluscan shell formation.

Supplementary note 3: Classification of regulatory divergence among functionally diverged paralogous genes

To define the regulatory divergence of biomineralization gene paralogs, we compared their expression levels and enrichment of active histone modifications (H3K4me3 and H3K27ac) between D-shaped larvae and adult mantle tissue. A gene was considered regulatory specialized for larval shell formation if it showed significantly higher expression and stronger enrichment of associated histone marks in larvae. In contrast, a gene was considered regulatory specialized for adult shell formation if it exhibited higher expression and histone signals in the adult mantle. Gene pairs with no significant differences in both expression and regulatory marks between stages were defined as shared regulators for both larval and adult shell formation. Following this framework, we defined three categories of regulatory divergence among functionally specialized paralogous gene pairs:

a) No divergence (no): both genes exhibit similar regulatory profiles across stages, with no significant differences in H3K4me3 or H3K27ac levels between larval and adult stages. This suggests a lack of regulatory asymmetry, despite functional divergence.
b) One-sided divergence (one): only one gene in the pair shows stage-specific regulatory enrichment, indicated by a marked increase in active histone modifications in either larvae or adults. This suggests that divergence in only one paralog may drive functional specialization.
c) Both-sided divergence (both): both genes exhibit distinct regulatory profiles

between stages, with one gene marked in larvae and the other in adults. This indicates coordinated, reciprocal regulatory specialization, where both paralogs have acquired distinct cis-regulatory landscapes adapted to stage-specific functions.

These classifications allowed us to systematically link expression specialization with regulatory evolution and support the role of epigenetic remodeling in the divergence of biomineralization gene function across oyster life stages.

Supplementary references

Bai Y, *et al*. Multi-omic insights into the formation and evolution of a novel shell microstructure in oysters. *BMC Biol*. 2023:21:204. https://doi.org/10.1186/s12915-023-01706-y.

Manni M, Berkeley MR, Seppey M, Simão FA, Zdobnov EM. BUSCO update: Novel and streamlined workflows along with broader and deeper phylogenetic coverage for scoring of eukaryotic, prokaryotic, and viral genomes. *Mol Biol Evol*. 2021:38:4647–4654. https://doi.org/10.1093/molbev/msab199.

Miglioli A, *et al*. The Mediterranean mussel *Mytilus galloprovincialis*: a novel model for developmental studies in mollusks. *Development*. 2024:151:dev202326. https://doi.org/10.1242/dev.202256.

Wang J, *et al*. Evolutionary transcriptomics of metazoan biphasic life cycle supports a single intercalation origin of metazoan larvae. *Nat Ecol Evol*. 2020:4:725–736. https://doi.org/10.1038/s41559-020-1138-1.
